# Supplementary material for: Pharmacology of Sedating and Anesthetic Agents: A Case-Based Flipped Classroom Exercise for Preclinical Medical Students
Source: MedEdPORTAL. 2024 Nov 8;20:11462. doi: 10.15766/mep_2374-8265.11462 (PMC11543632; doi:10.15766/mep_2374-8265.11462)
Supplement: Supplementary file 1 — Study Guide.docxPresession Readiness Quiz.docxIn-Class Student Worksheet.docxClinical Case Slides.pptxFacilitator Guide.docxPostsession Consolidation Quiz.docxPostsession Satisfaction Survey.docx [file mep_2374-8265.11462-s001.zip › A. Study Guide.docx]

**Pharmacology of Sedating and Anesthetic Agents – Study Guide**

**Instructions:** This study guide should be reviewed in the 2 weeks prior to the in-class flipped classroom session. It is intended to provide an outline of the foundational knowledge that should be acquired before the start of the session as well as to guide self-study of the topics.

**Anxiolytic & Hypnotic Drugs**

# Goals of sedation and anesthesia: anxiety reduction, lack of awareness, anterograde amnesia, and analgesia

- **Review of physiology: neurotransmission**
  - Inhibitory and excitatory neurotransmitters regulate behavioral process including consciousness, sleep, memory, mood, and sensation
    - **γ-aminobutyric acid (GABA)** is the major inhibitory neurotransmitter
    - **Glutamate** is the primary excitatory neurotransmitter
  - Neurotransmitters elicit inhibitory or excitatory responses by altering the conductance of ion-selective channels
    - Inhibitory: Hyperpolarize membrane potential further from the action potential threshold
    - Excitatory: Depolarize membrane closer to action potential threshold
  - Physiology of GABAergic neurotransmission
    - Most CNS neurons express GABA receptors
    - Drugs modulating GABA receptors influence arousal, attention, memory formation, anxiety, sleep, and muscle tone
    - Two types of GABA receptors:
      - GABA_A_: *Ionotropic receptor* with a central chloride-selective ion pore that opens when two GABA agonists bind on the extracellular portion of the receptor
        - GABA_A_ are the most common GABA receptors in the CNS and the target of drugs acting at GABA receptors
      - GABA_B:_ *G protein-coupled receptors* that activate potassium channels through second messengers mostly in the spinal cord
- **GABA_A_ Receptor Modulators**
  - **Benzodiazepines** and **barbiturates** are modulators of the GABA_A_ receptor
  - Benzodiazepines have sedative, muscle relaxant, amnestic, anxiolytic effects and at high doses can cause hypnosis and coma
  - Barbiturates are more commonly used for control of epilepsy and induction of general anesthesia
- **Benzodiazepines**
  - Positive allosteric GABA_A_ receptor modulators by enhancing receptor channel action in the presence of GABA
    - Low GABA concentrations: increase the frequency of channel opening
    - High GABA concentrations: receptor deactivation is slowed
  - Net increase in Cl^-^ influx causing neuron hyperpolarization
  - Do not activate GABA_A_ in the absence of GABA
  - Clinical uses: sleep enhancers, anxiolytics, sedation, anti-epileptics, muscle relaxants, and prevention/treatment of alcohol withdrawal
  - Achieves anxiolytic effect via inhibitory action in limbic system, CNS region controlling emotional behavior, due to high density of GABA_A_ receptors
  - Routes: oral, transmucosal, intravenous, intramuscular
  - Metabolized by hepatic P450 isozyme CYP3A4

| **Benzodiazepine** | **Clinical Uses** | **Duration** |
| --- | --- | --- |
| Midazolam | Procedural/ICU sedation,  anterograde amnesia | Short (minutes) |
| Alprazolam | Anxiety disorders, panic disorder | Intermediate (hours) |
| Lorazepam | Anxiety disorders, seizures,  alcohol withdrawal | Intermediate (hours) |
| Temazepam | Insomnia | Intermediate (hours) |
| Chlordiazepoxide | Alcohol withdrawal | Long (day+) |
| Clonazepam | Anxiety disorders, seizures | Long (day+) |
| Diazepam | Anxiety disorders, seizures,  muscle relaxant,  alcohol withdrawal | Long (day+) |

- - Caution for respiratory depression must be taken when combining with other sedatives and/or analgesics (e.g. opioids, antidepressants, alcohol, etc.) as well as in patients with respiratory, cardiovascular, liver, or brain dysfunction, or old age
  - Adverse reactions: Rebound insomnia, daytime drowsiness, confusion, unsteady gait, impaired motor skills, prolonged sedation, particularly in the elderly and with long-acting agents
  - Use should be intermittent and short-term due to high potential for tolerance, dependence, and addiction
  - Signs of withdrawal include severe anxiety, insomnia, tremor, delusions, and seizures
  - Withdrawal occurs sooner with abrupt discontinuation of short-acting agents
  - All benzodiazepines cross the placenta, can cause respiratory depression in a newborn, and are not recommended in pregnant women
  - **Flumazenil**
    - Competitive antagonist at benzodiazepine binding site on GABA_A_ receptor
    - Antidote to benzodiazepine overdose
    - May precipitate severe withdrawal symptoms including seizures in patients with benzodiazepine dependence, alcohol dependence, or prior seizure history
    - Short duration of action, may necessitate multiple doses to prevent recurrence of effects
- **Non-benzodiazepines or “Z-drugs”**

| **Non-benzodiazepines** |
| --- |
| Zolpidem |
| Zaleplon |
| Zopiclone |
| Eszopiclone |

- - Class of “non-benzodiazepine hypnotics” acting at the benzodiazepine receptor, selective for receptors containing α1 subunits
    - α1 subunits 🡪 less muscle relaxant and anxiolytic effects
  - Clinical use: short-term treatment of **insomnia**
  - Route: oral only
  - Adverse reactions: tolerance, dependence, and amnesia. When combined with benzodiazepines, may cause sleepwalking, sleep-driving, and sleep-eating
  - Unlike benzodiazepines and barbiturates, there is no alteration of sleep stages and therefore induce a more physiologic sleep
- **Barbiturates**

| **Anesthetic barbiturates** | |
| --- | --- |
| Methohexital | Very short (minutes) |
| Pentobarbital | Intermediate (hours) |

- - Enhance the efficacy of GABA by allosterically binding to the GABA receptor on the β subunit increasing the duration the Cl^-^ channel stays open, permitting a greater influx of Cl^-^ ions
    - **Anesthetic barbiturates:** act as both agonists at GABA_A_ receptors and as enhancer of receptor response to GABA
    - **Anticonvulsant barbiturates:** less direct agonism
  - Barbiturates also affect receptors involved in excitatory neurotransmission such as decreased activation of glutamate-sensitive AMPA receptors

| **Anticonvulsant barbiturates** | |
| --- | --- |
| Phenobarbital | Long-acting (days) |

- - Widespread CNS action including spinal cord, brainstem (reticular activating system), and brain (cortex, thalamus, cerebellum)
  - Cause sedation, amnesia, loss of spinal cord reflexes and loss of consciousness with anesthetic barbiturates
  - Clinical uses: induction of general anesthesia and seizure control. No analgesic effects
  - Duration of action depends on rapidity with which it is redistributed from the brain to other compartments
    - **Anesthetic barbiturates:** lipid-soluble, therefore enters brain rapidly then quickly redistribute away from the brain to other tissues
    - **Anticonvulsant barbiturates:** less lipid-soluble, therefore dissociate more slowly from brain tissue
  - Routes: oral, intravenous, intramuscular
  - Hepatic metabolism and renal excretion
    - Cytochrome P450 enzymes CYP3A4, CYP3A5, CYP3A7
    - Auto-induction of these enzymes 🡪 tolerance and diminished action of other drugs metabolized by the same isozymes
  - Adverse reactions: tolerance, dependence, profound hypnosis, coma, respiratory depression, and death
  - Caution must be taken when combining with other sedatives and/or analgesics (e.g., benzodiazepines, opioids, antidepressants, alcohol, etc.) as well as in patients with respiratory, cardiovascular, liver, or brain dysfunction, or old age
  - Signs of withdrawal include severe anxiety, insomnia, tremor, delusions, seizures, and cardiac arrest
- **Propofol**
  - GABA_A_ receptor positive allosteric modulator and at high doses may behave as a GABA_A_ receptor agonist, activating the GABA receptor in the absence of GABA
  - Prepared in an emulsion of soybean, glycerol and lecithin (egg yolk) giving it a characteristic milky white color
    - Supports bacterial growth if not kept sterile
    - Source of calories due to high lipid content, particularly if infused for many days
  - Most widely used anesthetic induction agent but provides no analgesic effect
  - Can also be used for maintenance of general anesthesia, procedural sedation, and ICU sedation as a prolonged infusion
  - Route: Intravenous
  - Rapidly redistributes away from the brain and is metabolized by the liver, with a short duration of action (minutes) if given as a single dose
  - Adverse reactions: Pain at injection site, hypotension from vasodilation and cardiac depression, **respiratory depression/arrest!**
  - Rarely **green urine** with prolonged infusions
  - Propofol infusion syndrome is rare and occurs with long-term high dose sedation, more commonly in children: Muscle breakdown (rhabdomyolysis), metabolic acidosis, kidney failure, cardiac failure
- **Etomidate**
  - Modulator at the GABA_A_ β subunit and at higher doses may function as a GABA agonist in the absence of GABA
  - Short acting agent (minutes) used for induction of general anesthesia, commonly in unstable patients due to its relatively minimal cardiopulmonary depressive effects
  - Adverse reactions:
    - Inhibits enzyme *11β-hydroxylase*, temporarily suppressing adrenal steroid production potentially resulting in refractory hypotension
    - Burns on injection
    - Increases likelihood of post-operative nausea and vomiting
- **NMDA Receptor Physiology**
  - Main subtypes of glutamate-gated ion channels: AMPA and **NMDA**
  - NMDA receptors 🡪 Expressed in hippocampus, cerebral cortex, and spinal cord
  - NMDA receptor channel pores are blocked by Mg2+ at rest. Activation requires simultaneous binding of glutamate and glycine as well as nearby depolarization of AMPA receptors
    - Opening causes K+ efflux along with Na+ and Ca2+ influx
- **Ketamine**
  - **NMDA receptor antagonist** 🡪 thought to block the effects of glutamate at the NMDA receptor but likely binds at several allosteric sites
  - Causes ***dissociative anesthesia*** 🡪 patients may appear awake but feel dissociated from surroundings and experience analgesia and amnesia
  - Cardiac and respiratory function largely remain intact
  - Clinical uses: Sedation, analgesia, and induction of general anesthesia
  - Routes: Intravenous, intramuscular, sublingual, and topical
  - Metabolism: CYP3A4 and CYP2B6
  - Adverse reactions: agitation, delirium, confusion, hallucinations, hypertension/tachycardia, salivation
    - Nystagmus 🡪 Eyes make repetitive uncontrolled movements
  - May be indicated for treatment-resistant depression in select circumstances

**Depolarizing & Non-Depolarizing Muscle Relaxants**

| **MUSCLE RELAXANT DRUGS** | |
| --- | --- |
| **Depolarizing** | **Non-Depolarizing** |
| **Short-acting** | **Short-acting** |
| Succinylcholine | Mivacurium |
|  | **Intermediate-acting** |
|  | Atracurium |
|  | Cisatracurium |
|  | Vecuronium |
|  | Rocuronium |
|  | **Long-acting** |
|  | Pancuronium |

## **Physiology review: Neuromuscular Junction**

- - Neuromuscular junction is where neurons associate with muscle fibers and form synaptic clefts
  - When the nerve depolarizes, influx of Ca2+ ions occurs through voltage-gated calcium channels into the nerve cytoplasm allowing storage vesicles to fuse with the terminal plasma membrane and release **acetylcholine** (ACh) into the synaptic cleft
  - The ACh binds **nicotinic ligand-gated cholinergic receptors** on the muscle motor end-plate
  - ACh receptors in the neuromuscular junction consist of five protein subunits: two α and three non-α subunits
  - The two identical α subunits can bind ACh
  - Both α subunit binding sites must be occupied simultaneously to induce a conformational change resulting in opening of the ion channel core (Na+ and Ca2+ flow in and K+ flows out) generating the end-plate potential
  - Once the end-plate potential becomes sufficiently strong, an action potential spreads over the surface of skeletal muscle with inflow of Na+, release of Ca2+ from the sarcoplasmic reticulum, and contractile proteins actin and myosin interact, causing muscle contraction
  - ACh is quickly degraded by the enzyme **acetylcholinesterase**, which is present in the motor end-plate near the ACh receptors, and therefore rapidly restores membrane repolarization

## **Neuromuscular Relaxation Medications: Common Clinical Uses**

- - Facilitate endotracheal intubation
  - Adjunct during general anesthesia to produce optimal surgical conditions (relaxation of skeletal muscles and inhibition of spontaneous breathing)
  - In patients in critical condition to facilitate mechanical ventilation in select patients
- Depolarizing and Non-depolarizing neuromuscular blocking agents are the two classes of drugs. Each drug exerts its effects at the nicotinic ACh receptor with the medications in each of the two categories having distinct mechanisms of action, clinical characteristics, and adverse reactions

## **Depolarizing Muscle Relaxant (Succinylcholine)**

- - Succinylcholine is the only depolarizing muscle relaxant in clinical use
  - Structurally resembles the ACh molecule and acts as a nicotinic ACh receptor agonist, generating a muscle action potential
  - Unlike ACh, succinylcholine is not metabolized by acetylcholinesterase, therefore the concentration remains high enough to cause prolonged depolarization, resulting in muscle relaxation as Na+ channels rapidly inactivate despite continued depolarization
  - Depolarizing neuromuscular blockade is referred to as a phase I block
  - If the post-junctional membrane becomes repolarized but still does not respond normally to acetylcholine, a phase II block has occurred (although phase II blocks do not always occur)
  - Rapid onset (seconds) and rapid offset (minutes)
  - Rapidly metabolized by plasma cholinesterases (not acetylcholinesterase!)
    - Plasma cholinesterases are alternatively termed pseudocholinesterases or butyrylcholinesterases
  - Can be administered via intravenous or intramuscular routes
  - Visible motor contractions called fasciculations can be seen before onset of clinical muscle relaxation
  - Associated with postoperative myalgias
  - May cause masseter muscle spasm making it difficult to open the patient’s mouth
  - Triggering agent for the condition malignant hyperthermia!
  - Normal muscle releases potassium during succinylcholine-induced depolarization, which may lead to hyperkalemia
  - No drug exists to reverse a depolarizing blockade
  - Contraindications:
    - Conditions associated with a chronic decrease in ACh release cause an increase in the number of ACh receptors, including receptors outside of the neuromuscular junction, termed “extrajunctional ACh receptors”, which have longer open-channel time when activated
    - Succinylcholine is contraindicated in patients with these conditions because of potentially exaggerated hyperkalemia and consequentially potential for cardiac arrest. These causative conditions include burn injuries, massive trauma/crush injuries, spinal cord injury, stroke, Guillain-barre syndrome, prolonged immobilization, myopathies [e.g. Duchenne’s muscular dystrophy]), among others.

## **Non-Depolarizing Muscle Relaxants (NDMR)**

- - NDMRs function as competitive antagonists at the nicotinic ACh receptor
  - These agents bind and prevent ACh from binding to its receptor. No end-plate potential develops, even if only one of the two α subunits is blocked
  - Small muscles of the face are most sensitive, followed by fingers, limbs, neck, trunk muscles, followed by intercostal muscles and lastly the diaphragm
  - Muscle groups recover in reverse order with function returning first at the diaphragm
  - Differences in onset, duration, rate of recovery, metabolism, and clearance influence the clinical decision to select one NDMR versus another
  - Two classes of NDMRs exist based on chemical structure:
    - Benzylisoquinolines (end with the suffix -curium): mivacurium, atracurium, cisatracurium
    - Aminosteroids (end with the suffix -curonium): vecuronium, rocuronium, pancuronium

## **Mivacurium**

- - Metabolized by pseudocholinesterase (note different name than acetylcholinesterase!)
  - Triggers histamine release from mast cells, which can result in bronchospasm, skin flushing, and hypotension from vasodilation
  - Fast onset (minutes) and brief duration of action (minutes)

## **Atracurium**

- - Undergoes degradation in plasma by organ-independent Hofmann elimination
  - Triggers histamine release from mast cells, which can result in bronchospasm, skin flushing, and hypotension from vasodilation

## **Cisatracurium**

- - Undergoes degradation in plasma by organ-independent Hofmann elimination
  - Not associated with significant histamine release

## **Vecuronium & Rocuronium**

- - Hepatically metabolized and metabolites undergo biliary and renal excretion
  - **Rocuronium** has an onset time that approaches succinylcholine (seconds), making it an alternative when the patient has a contraindication for succinylcholine, although duration of action of an intubating dose is long and can exceed an hour

## **Pancuronium**

- - Hepatically metabolized and metabolites undergo biliary and renal excretion
  - Vagolytic effect because of blockade at vagal muscarinic receptors causing tachycardia

## **Monitoring of Neuromuscular Blockade**

- - Peripheral nerve stimulation is used to monitor depth of blockade by neuromuscular blocking agents
  - Several patterns of electrical stimulation exist. Train-of-four is one common example of a stimulation pattern.
    - Train-of-four: a series of four stimulations over 2 seconds
  - Phase I neuromuscular blockade by a depolarizing agent does not exhibit fade with stimulation
  - The occurrence of fade with repeated nerve stimulation is characteristic of a non-depolarizing block or a phase II block by a depolarizing agent

**
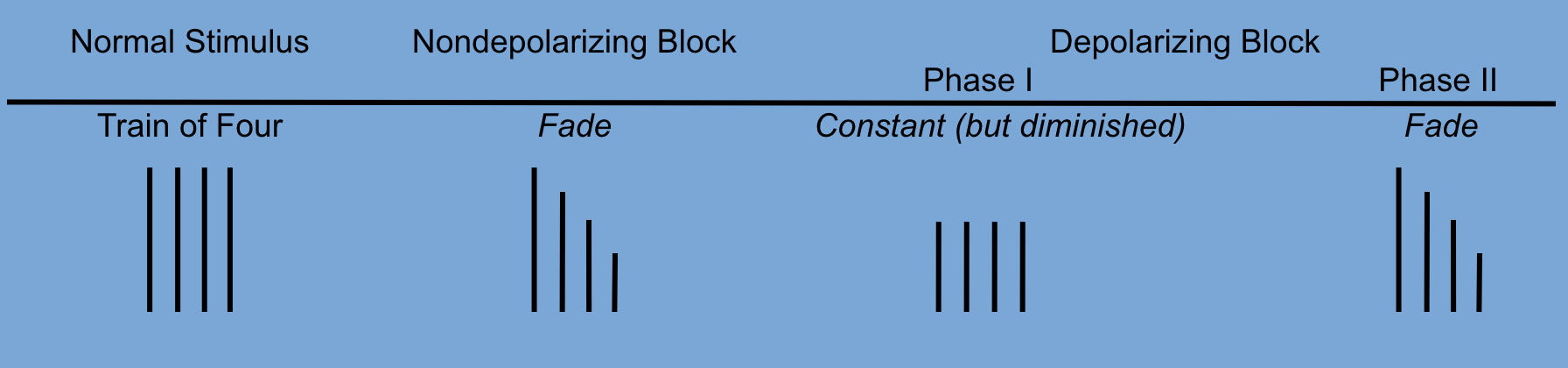
**

## **Reversal of Non-Depolarizing Muscle Relaxants**

- - Incomplete recovery from muscle relaxation, referred to as “residual neuromuscular blockade” or “post-procedural paralysis”, is associated with complications potentially in the form of respiratory distress, reintubation, and psychological distress
  - Reversal of the effects of NDMR blockade depends on the unbinding of the medications from the nicotinic ACh receptors, redistribution away from the neuromuscular junction, metabolism of the drug, and eventual excretion from the body
  - A reversal agent should always be administered to patients who have received a NDMR unless full reversal has been demonstrated or the postoperative plan includes continued intubation and continuous sedation

### **Cholinesterase inhibitors**

| **Cholinesterase inhibitors** |
| --- |
| **Neostigmine** |
| **Pyridostigmine** |
| **Physostigmine** |
| **Edrophonium** |

- - - Cholinesterase inhibitors inactivate acetylcholinesterase by reversibly binding to the enzyme
    - These agents indirectly increase the concentration of ACh available to compete with the NDMR agent, speeding recovery to normal neuromuscular transmission
    - The increase in acetylcholine caused by cholinesterase inhibitors affects more than just the nicotinic receptors in skeletal muscle, including muscarinic ACh receptors
      - Side effects:
        - Cardiovascular: Bradycardia and potentially cardiac arrest
        - Pulmonary: Bronchospasm and increased secretions
        - Gastrointestinal: Increased peristaltic activity (i.e. increased nausea, vomiting, and fecal incontinence)
      - Unwanted muscarinic side effects are counteracted by simultaneously administering an anticholinergic agent such as **atropine** or **glycopyrrolate**
    - Cholinesterase inhibitors prolong rather than reverse a phase I block by a depolarizing agent (succinylcholine), therefore cannot used to reverse succinylcholine
    - Cholinesterase inhibitors are also used in the diagnosis and management of the disease myasthenia gravis and the treatment of some dementias
  - **Sugammadex:** selective relaxant binding agent for reversal of rocuronium and vecuronium
    - Cyclodextrin molecule that binds rocuronium or vecuronium forming a complex, causing the NDMR to become incapable of binding at the acetylcholine receptor and the complex is transported away from the neuromuscular junction
    - The Sugammadex-NDMR complex is cleared rapidly and unchanged by renal excretion, therefore contraindicated in patients with renal failure
    - Sugammadex may decrease the efficacy of oral contraceptives (about seven days)
    - Small risk of bradycardia or anaphylaxis

**Inhaled Anesthetics**

## Stages of Anesthesia – Proceeds in order with increasing anesthetic depth and in reverse order upon awakening

- - Stage I: Analgesia and amnesia
  - Stage II: Excitation – delirium, combative behavior, irregular respiration
  - Stage III: Surgical anesthesia – loss of consciousness, regular respiration
  - Stage IV: Medullary depression – cardiovascular and respiratory depression/collapse

| **Volatile Anesthetics** | **Anesthetic Gases** |
| --- | --- |
| **Halothane** | **Nitrous oxide** |
| **Enflurane** | **Xenon** |
| **Isoflurane** |  |
| **Desflurane** |  |
| **Sevoflurane** |  |

## **Inhalational Anesthetics**

- - Exist as either **volatile anesthetics** or **anesthetic gases**
  - Inhalational anesthetics are non-flammable and non-explosive
  - Narrow therapeutic index: small concentration difference between concentration indicated for anesthesia and that of cardiopulmonary depression/collapse
  - Inhaled anesthetics absorbed via the lungs into the systemic circulation cause a dose-dependent CNS depression

## **Delivery of inhalational anesthetics**

- - Inhaled anesthetics can be used for induction or maintenance of surgical anesthesia
  - Administered in combination with other gases, one of which must be oxygen!
  - Unlike anesthetic gases, volatile agents exist in liquid state at room temperature and must be vaporized

| **Inhaled Anesthetic** | **MAC (%)** | **Blood—Gas Partition Coefficient (λ)** |
| --- | --- | --- |
| **Volatile agents** |  |  |
| Halothane | 0.75 | 2.3 |
| Isoflurane | 1 | 1.5 |
| Enflurane | 1.7 | 1.8 |
| Sevoflurane | 2 | 0.65 |
| Desflurane | 6 | 0.42 |
| **Anesthetic gases** |  |  |
| Nitrous oxide | 104 | 0.47 |
| Xenon | 70 | 0.14 |

## **Minimum Alveolar Concentration (MAC)**

- - Defined as the end-tidal concentration (i.e. concentration measured upon exhalation) of inhaled anesthetic agent required to prevent movement in 50% of patients upon surgical stimulation
  - It is a measure of the anesthetic potency of the agent
  - MAC is small for potent anesthetics and large for less potent agents
  - MAC required to achieve a desired depth of anesthesia is different for each individual based on unique patient-specific characteristics
  - Characteristics that increase MAC require delivery of a higher percentage of inhalational agent to achieve the same desired effect
    - Increases MAC: acute stimulant intoxication (cocaine, amphetamines, etc.), chronic alcohol use, hyperthermia, red hair color
    - Decreases MAC: increasing age, pregnancy, hypothermia, hypotension/sepsis, acute alcohol intoxication, co-administered anesthetic/sedating agents (i.e. benzodiazepines, opioids, hypnotics, etc.), chronic stimulant use (cocaine, amphetamines, etc.)
  - MAC is independent of gender or weight
  - MAC is additive if multiple agents are administered

## **Blood-gas partition coefficient (λ)**

- - Time to onset and offset is determined by **solubility** in blood, measured by the blood-gas partition coefficient (λ)
    - Lower solubility (low blood:gas partition coefficient) means fewer anesthetic molecules are required in blood to achieve equilibrium in CNS 🡪 rapid onset
    - Lower solubility (low blood:gas partition coefficient) results in faster exhalation 🡪 rapid offset
  - Peak effect occurs and abates when the concentration (partial pressure) of anesthetic in the alveoli equilibrates with that in the CNS
    - Induction: P_alveoli_ > P_blood_ > P_brain_
    - Equilibrium: P_alveoli_ = P_blood_ = P_brain_
    - Recovery: P_alveoli_ < P_blood_ < P_brain_
- **Nitrous Oxide** – “Laughing gas”
  - Known NMDA antagonist but all mechanisms are not yet understood
  - Cannot produce general anesthesia alone – impossible to reach the theoretical MAC value (104%) and even approaching the MAC value would require a low oxygen percentage incompatible with life
  - Often combined with other agents for additive effect
  - Inhibits enzyme *methionine synthase*, the enzyme regulating vitamin B12 and folate metabolism, which could cause megaloblastic anemia, vitamin B12 deficiency, and/or birth defects with chronic or prolonged exposure
  - Increases likelihood of post-operative nausea and vomiting

## **Volatile Anesthetics:**

- - Mechanism not yet well understood and likely multifactorial
  - Associated effects:
    - Hypotension: Combination of cardiac depression and vasodilation
    - Bronchodilation
    - Decreased respiratory drive with rapid shallow breathing until respiration stops at higher doses
  - Triggering agents for **malignant hyperthermia**
  - Steep dose-response curves with narrow therapeutic range and small window between general anesthesia and cardiopulmonary depression/collapse
  - No antagonists exist for inhalational anesthetics
  - **Halothane**
    - No longer in clinical use
    - Hepatotoxicity – “Halothane hepatitis”
      - Severe acute liver failure can occur
      - Attributed to damage by “free radicals” and an immune-mediated response
      - 1 in 20,000-35,000 halothane-based anesthetics
    - Sensitizes the myocardium to epinephrine and other circulating catecholamines, which can lead to arrhythmias
    - Bronchodilation – not irritating to the airway
  - **Isoflurane:** Pungent odor – irritating to the airway
  - **Sevoflurane:** Bronchodilation – not irritating to the airway
  - **Desflurane**
    - Very rapid onset and offset
    - Pungent odor – irritating to the airway
    - Low volatility requires special heated vaporizer
  - **Enflurane**
    - No longer in clinical use
    - Causes CNS stimulation and tonic-clonic movements
    - Pungent odor – irritating to the airway

## **Malignant Hyperthermia**

- - Inherited disorder of increased skeletal muscle metabolism when exposed to volatile anesthetics and/or succinylcholine. Patients are otherwise asymptomatic!
  - Incidence of ~1 in every 100,000 anesthetics in adults and ~1 in every 30,000 anesthetics in children
  - Mortality greater than 80% if untreated and less than 5% if treated
  - People with this genetic susceptibility are otherwise normal when not exposed to triggering agents
  - Caused by genetic mutations in genes coding for the ryanodine receptor (often RYR1 but more than 25 mutations are known to exist)
  - Usually autosomal dominant inheritance with variable penetrance and a male predominance (mutation dependent)
  - Mutated ryanodine receptor channel opens more easily and stays open longer flooding the cytosol with Ca^2+^ resulting in sustained muscle contraction and catabolism
  - Symptoms: tachycardia, increased exhaled CO2, muscle rigidity, hyperthermia, muscle breakdown, kidney failure, and eventual cardiovascular collapse if untreated
  - **Dantrolene**
    - Only approved treatment for malignant hyperthermia
    - Ryanodine receptor antagonist: Depresses excitation-contracting coupling in skeletal muscle by decreasing intracellular Ca^2+^ concentration
    - Oral formulation available to treat muscle spasticity associated with chronic neurologic conditions such as multiple sclerosis, cerebral palsy, strokes, and paraplegia

## **Environmental Considerations**

- - All inhaled anesthetics are greenhouse gases
  - Inhalational agents are minimally metabolized with most of the administered anesthetic released back into the atmosphere on exhalation
  - Atmospheric longevities differ between agents
    - Nitrous oxide: 114 years
    - Desflurane: 10 years
    - Sevoflurane: 3.6 years
    - Isoflurane: 1.2 years
  - Warming potential of nitrous oxide is 300 times stronger than CO2
  - Total contribution by anesthetic agents to greenhouse effect is small, with nitrous oxide having the greatest effect at less than 0.05%

# **Local Anesthetics**

## Physiology review: generation of action potentials

- - In response to a painful stimulus (depolarizing stimulus), Na+ conductance into the neuron increases rapidly because of rapid opening of voltage-gated Na+ channels
  - Na+ conductance then decreases because of Na+ channel inactivation
  - K+ outward conductance then increases concurrently until the membrane repolarizes

## Local Anesthetics (LAs) – Mechanism of Action

- - Exert effects on the **cytoplasmic side** of the voltage-gated Na+ channel
  - LAs must enter into, diffuse across, and dissociate from the cell membrane into the cytoplasm to exert their effects
  - LAs **block voltage-gated Na+ channels**, preventing a transient increase in permeability of the nerve membrane to Na+ required for action potential generation and propagation

## Modulated Receptor Hypothesis:

- - Voltage-gated Na+ channels exist in 3 main conformations: open, inactivated, and resting
  - The different conformational states bind LAs with different affinities
  - LAs have a higher affinity for open and inactivated states than for the resting state
  - Binding of LAs to the Na+ channel causes physical occlusion of the pore and also prevents the conformational change that allows for channel activation
  - For a Na+ channel to reopen, the LA must dissociate from the channel and allow the channel to return to the resting state

## Local Anesthetics – Routes of Administration

- - Topical or transdermal: Applied to skin without mechanical penetration (e.g. patches for chronic localized pain or creams for burns or small cuts)
  - Subcutaneous infiltration: Injection in subcutaneous space (e.g. skin procedures such as suturing a cut)
  - Perineural: Injection targeting specific nerves to block desired nerve distribution (e.g. dental and regional anesthetic procedures such as “nerve blocks”)
  - Neuraxial: often used for labor analgesia, obstetric surgery and select other surgical procedures
    - Epidural: injection of LA into epidural space to anesthetize spinal nerve roots
    - Spinal/intrathecal: injection of LA into subarachnoid space (into cerebral spinal fluid) to block spinal nerves
  - Intravenous: primarily for antiarrhythmic properties with some potential analgesic effects

## Local Anesthetics – Classifications by Chemical Structure

- - LAs have three structural components: an aromatic group, an amine group, and an ester or amide linkage connecting the two
  - The **ester** or **amide** linkage is the differentiating factor dividing LAs into two groups with distinct clinically relevant properties
  - LA naming classification
    - All names of LAs end with the suffix **-caine**
    - **Esters** have one “i” in the name (e.g. benzocaine, chloroprocaine, cocaine, procaine)
    - **Amides** have two “i"s in the name (e.g. bupivacaine, lidocaine, ropivacaine)

## Amine group properties

- - - The amine group exists in either a protonated form or a deprotonated form (neutral or basic)
    - LAs are weak bases**.** At a physiologic pH of 7.4, LAs with a higher pKa have a larger fraction of molecules existing in the protonated form
    - The neutral form diffuses across membranes more easily than positively charged forms, however, positively charged drugs bind with higher affinity to the target binding site
    - Moderately weak bases are the most effective at physiologic pH because they rapidly diffuse across membranes, can easily gain a proton to become positively charged, and bind with high affinity to the cytoplasmic side of the Na+ channel
    - Clinical relevance: Alterations in local physiologic pH may alter effects of the drugs (e.g. injection of LA near an infected wound, which may be more acidic in nature, may be less likely to achieve the desired effect)

## Local Anesthetics – Onset and Duration

- - **Onset** is heavily determined by pKa with a pKa closer to physiologic pH resulting in more rapid onset
  - **Duration** of action is limited by distribution of the LA away from the site of action
    - Co-administration of vasoconstrictors (i.e. epinephrine) increases duration of effect
    - Vasoconstrictors can cause tissue hypoxia in areas of poor oxygen delivery

## Local Anesthetics – Metabolism

- - Ester-linked LAs are metabolized by tissue and plasma esterases (alternatively termed pseudocholinesterases or butyrylcholinesterases), which is rapid
  - Amide-linked LAs are metabolized in the liver by cytochrome P450 enzymes
    - Caution with amide-linked LAs should be taken in patients with liver disease/cirrhosis as prolonged effects and toxicity at lower doses can occur

## Local Anesthetics – Allergic Reactions

- - Hypersensitivity is rare and if it occurs is almost always associated with ester-linked LAs
  - **Para-aminobenzoic acid (PABA)** is a metabolite of ester-linked LAs and is a known allergen

## Local Anesthetics are also Antiarrhythmic Drugs

- - LAs are not selective for the voltage-gated Na+ channels on pain fibers and therefore also block conduction in sensory, motor, autonomic fibers, skeletal muscle and cardiac muscle
  - Action of LAs on cardiac Na+ channels can serve therapeutic effects to treat arrhythmias or cause adverse effects if unintentionally blocked
  - LAs function as Vaughan-Williams Class 1 antiarrhythmic agents

## Cocaine

- - First LA noted to have numbing properties around the 1860’s
  - Cocaine is the only LA that inhibits reuptake of monoamine neurotransmitters (dopamine, norepinephrine, epinephrine, serotonin), resulting in vasoconstriction and tachycardia
  - Inhibition of reuptake of dopamine activates the reward pathway and causes a euphoric feeling leading to high addiction potential

## Lidocaine

- - Rapid onset with medium duration of action (1-2 hours)
  - Some analgesic uses include local infiltration, peripheral nerve blocks (perineural infiltration), epidural, spinal, and topical anesthesia
  - Used clinically as Class 1B antiarrhythmic agent

## Bupivacaine

- - Long duration of action (2-3+ hours)
  - High potency causes higher degree of cardiotoxicity and is very slow to dissociate from Na+ channels on cardiac myocytes

## Local Anesthetic Systemic Toxicity (LAST)

- - The amount of LA that enters the systemic circulation and the potency of the drug together determine the systemic toxicity
  - Vascularity of the injection site, drug concentration, and the co-administration of a vasoconstrictor (i.e. epinephrine) all influence the rate and degree of systemic absorption
    - Common sites of injection from most to least vascular: intercostal, caudal epidural space, lumbar epidural space, brachial plexus, femoral nerve, and subcutaneous infiltration
  - Symptoms of LAST in order of presentation: perioral numbness (lips and tongue), lightheadedness, visual and/or auditory disturbances, muscular twitching, loss of consciousness, seizures, respiratory arrest, cardiac arrest

## Lipid emulsion

- - - “Lipid rescue therapy” is indicated in LAST in the form of a 20% lipid emulsion for IV use
    - Emulsion of soybean oil, egg phospholipids, and glycerin
    - Mechanism of action is not completely understood but is believed to form a “lipid sink” facilitating removal of lipophilic toxins
    - Treatment should also include seizure suppression, airway management, and cardiopulmonary support as needed

**Study Guide References:**

1. Miller RD, Pardo M, Stoelting RK. *Basics of anesthesia*. 6th ed. Elsevier/Saunders; 2011:xii, 817 p.
2. Butterworth JF, Mackey DC, Wasnick JD. *Morgan and Mikhail's clinical anesthesiology*. Seventh edition. ed. Lange medical book. McGraw Hill; 2022:1 online resource (1458 pages).
3. Clark MA. *Lippincott's illustrated reviews. Pharmacology*. 5th ed. Lippincott's illustrated reviews. Wolters Kluwer Health/Lippincott Williams & Wilkins; 2012:xii, 612 p.
