# Supplementary material for: Pharmacology of Sedating and Anesthetic Agents: A Case-Based Flipped Classroom Exercise for Preclinical Medical Students
Source: MedEdPORTAL. 2024 Nov 8;20:11462. doi: 10.15766/mep_2374-8265.11462 (PMC11543632; doi:10.15766/mep_2374-8265.11462)
Supplement: Supplementary file 1 — Study Guide.docxPresession Readiness Quiz.docxIn-Class Student Worksheet.docxClinical Case Slides.pptxFacilitator Guide.docxPostsession Consolidation Quiz.docxPostsession Satisfaction Survey.docx [file mep_2374-8265.11462-s001.zip › F. Postsession Consolidation Quiz.docx]

**Pharmacology of Sedating and Anesthetic Agents – Post-Session Consolidation Quiz Questions**

**Instructions:** These quiz questions should be administered at the end of the in-person session. Students should be allotted 10 minutes for completion of the quiz questions. After submission, students may review the correct answers with the corresponding explanations.

1. A 38-year-old woman presents for a needle biopsy of a thyroid nodule. The interventional radiologist administers midazolam for moderate sedation. Which of the following effects should be anticipated with use of this agent?
   1. Tachypnea
   2. Tachycardia
   3. Seizures
   4. Anterograde Amnesia
2. A 21-year-old man presents to the operating room for a tonsillectomy. The anesthesiologist measures the patient’s train-of-four at baseline, represented as (A) in the image below. After induction of general anesthesia, the train-of-four is measured again, represented as (B). Which of the following agents was likely administered during induction?


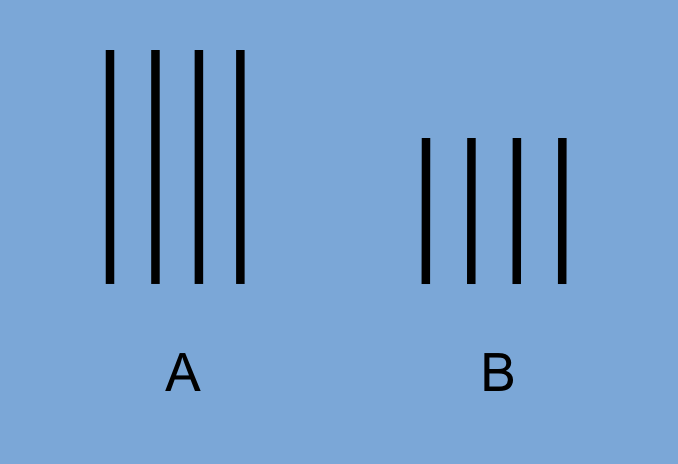


- 1. Sugammadex
  2. Cisatracurium
  3. Succinylcholine
  4. Rocuronium

1. A 22-year-old man presents to the trauma center in respiratory distress immediately following a motorcycle collision and requires endotracheal intubation. The anesthesiologist administers an intubating dose of succinylcholine. Which of the following potential adverse reactions is NOT directly associated with succinylcholine?
   1. Hallucinations
   2. Myalgias
   3. Hyperkalemia
   4. Malignant Hyperthermia
2. A 28-year-old woman presents to the labor and delivery unit in labor and requests an epidural for labor analgesia. She reports having had an allergic reaction to the local anesthetic tetracaine causing throat swelling and difficulty breathing. Which of the following local anesthetics would be best avoided by the anesthesiologist when placing the epidural?
   1. Ropivacaine
   2. Chloroprocaine
   3. Bupivacaine
   4. Lidocaine
3. A 24-year-old man presents to the operating room for repair of a femur fracture sustained after falling off his bicycle. He reports a family history of complications from anesthesia including multiple family members who have experienced malignant hyperthermia. Which of the following agents would be best avoided by the anesthesiologist?
   1. Nitrous oxide
   2. Propofol
   3. Succinylcholine
   4. Cisatracurium

**Pharmacology of Sedating and Anesthetic Agents – Consolidation Quiz Questions with Answers and Explanations**

1. A 38-year-old woman presents for a needle biopsy of a thyroid nodule. The interventional radiologist administers midazolam for moderate sedation. Which of the following effects should be anticipated with use of this agent?
2. Tachypnea
3. Tachycardia
4. Seizures
5. Anterograde Amnesia

**Answer: D**

**Rationale**: Midazolam is a common agent belonging to the benzodiazepine class of drugs used for procedural and ICU sedation. While it shares common effects with other benzodiazepines, it specifically can cause anterograde amnesia. Tachypnea and tachycardia are unlikely as the patient’s respiratory rate and heart rate would typically be expected to slow given the anxiolytic effect of Midazolam. Midazolam is occasionally used to treat seizures and thus would not be expected to cause a seizure.

2. A 21-year-old man presents to the operating room for a tonsillectomy. The anesthesiologist measures the patient’s train-of-four at baseline, represented as (A) in the image below. After induction of general anesthesia, the train-of-four is measured again, represented as (B). Which of the following agents was likely administered during induction?


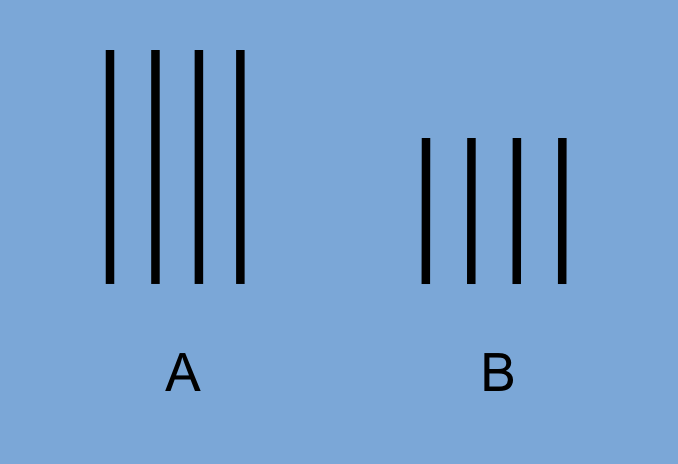


1. Sugammadex
2. Cisatracurium
3. Succinylcholine
4. Rocuronium

**Answer:** C

**Rationale**: The change in train-of-four represented by (A) and (B) demonstrates a diminished signal after induction of anesthesia, which is consistent with administration of a depolarizing neuromuscular block agent (e.g. succinylcholine). If the drug administered were a non-depolarizing neuromuscular blocking agent (e.g. cisatracurium or rocuronium), one would have expected fade to be present in (B). Sugammadex is a selective relaxant binding agent, which reverses the effect of some non-depolarizing neuromuscular blocking agents and therefore would not be administered with induction of anesthesia and would not cause the change in train-of-four depicted in this figure.

3. A 22-year-old man presents to the trauma center in respiratory distress immediately following a motorcycle collision and requires endotracheal intubation. The anesthesiologist administers an intubating dose of succinylcholine. Which of the following potential adverse reactions is NOT directly associated with succinylcholine?

1. Hallucinations
2. Myalgias
3. Hyperkalemia
4. Malignant Hyperthermia

**Answer:** A

**Rationale**: Myalgias, hyperkalemia, and malignant hyperthermia are known adverse reactions of succinylcholine, whereas hallucinations are not.

4. A 28-year-old woman presents to the labor and delivery unit in labor and requests an epidural for labor analgesia. She reports having had an allergic reaction to the local anesthetic tetracaine causing throat swelling and difficulty breathing. Which of the following local anesthetics would be best avoided by the anesthesiologist when placing the epidural?

1. Ropivacaine
2. Chloroprocaine
3. Bupivacaine
4. Lidocaine

**Answer:** B

**Rationale**: Ester-linked local anesthetics are far more likely to cause allergic reactions when compared to amide-linked local anesthetics. Thus, when a hypersensitivity reaction occurs after exposure to a local anesthetic, an ester-linked local anesthetic is more commonly suspected. Ester-linked local anesthetics have a metabolite known as para-aminobenzoic acid (PABA), which is a known allergen. This patient’s previous allergic reaction was to tetracaine, which is an ester-linked local anesthetic and is therefore consistent with this rationale. All other options listed are amide-linked local anesthetics, which do not produce this metabolite and would be considered safe for administration in this patient.

5. A 24-year-old man presents to the operating room for repair of a femur fracture sustained after falling off his bicycle. He reports a family history of complications from anesthesia including multiple family members who have experienced malignant hyperthermia. Which of the following agents would be best avoided by the anesthesiologist?

1. Nitrous oxide
2. Propofol
3. Succinylcholine
4. Cisatracurium

**Answer:** C

**Rationale**: Of the agents listed, succinylcholine is the only one known to be a triggering agent for malignant hyperthermia. The only known triggering agents for malignant hyperthermia are succinylcholine and volatile anesthetics (e.g. sevoflurane, isoflurane, desflurane, etc.), which does not include the anesthetic gas nitrous oxide.
