# Supplementary material for: Pharmacology of Sedating and Anesthetic Agents: A Case-Based Flipped Classroom Exercise for Preclinical Medical Students
Source: MedEdPORTAL. 2024 Nov 8;20:11462. doi: 10.15766/mep_2374-8265.11462 (PMC11543632; doi:10.15766/mep_2374-8265.11462)
Supplement: Supplementary file 1 — Study Guide.docxPresession Readiness Quiz.docxIn-Class Student Worksheet.docxClinical Case Slides.pptxFacilitator Guide.docxPostsession Consolidation Quiz.docxPostsession Satisfaction Survey.docx [file mep_2374-8265.11462-s001.zip › G. Postsession Satisfaction Survey.docx]

**Pharmacology of Sedating and Anesthetic Agents – Post-Session Satisfaction Survey**

**Instructions:** This survey should be administered at the conclusion of the in-person session without a time limit to gauge learner satisfaction.

|  | 1  Strongly Disagree | 2  Disagree | 3  Neither Agree nor Disagree | 4  Agree | 5  Strongly Agree |
| --- | --- | --- | --- | --- | --- |
| - - - 1. The assigned reading material adequately prepared me for the flipped classroom session. |  |  |  |  |  |
| 1. The flipped classroom session facilitated my learning of the basic pharmacology relevant to sedating and anesthetic drugs. |  |  |  |  |  |
| 1. The flipped classroom session facilitated my understanding of the clinical application of sedating and anesthetic drugs. |  |  |  |  |  |
| 1. The time spent discussing the case with my peers contributed to my understanding of the subject matter. |  |  |  |  |  |
| 1. Please provide additional comments. | | | | | |
