# Supplementary material for: Pharmacology of Sedating and Anesthetic Agents: A Case-Based Flipped Classroom Exercise for Preclinical Medical Students
Source: MedEdPORTAL. 2024 Nov 8;20:11462. doi: 10.15766/mep_2374-8265.11462 (PMC11543632; doi:10.15766/mep_2374-8265.11462)
Supplement: Supplementary file 1 — Study Guide.docxPresession Readiness Quiz.docxIn-Class Student Worksheet.docxClinical Case Slides.pptxFacilitator Guide.docxPostsession Consolidation Quiz.docxPostsession Satisfaction Survey.docx [file mep_2374-8265.11462-s001.zip › B. Presession Readiness Quiz.docx]

**Pharmacology of Sedating and Anesthetic Agents – Pre-Session Readiness Quiz Questions**

**Instructions:** These quiz questions should be administered prior to the start of the in-person session. Students should be allotted 10 minutes for completion of the quiz questions. After submission, students may review the correct answers with the corresponding explanations.

1. A 55-year-old woman arrives to the hospital prior to undergoing spine surgery for chronic lower back pain. She states she is very nervous about the upcoming procedure. The anesthesiologist administers midazolam for pre-procedural anxiolysis. Which of the following describes the receptor and mechanism of this agent?
   1. GABA_A_ receptor: increases the duration of Cl channel opening
   2. GABA_A_ receptor: increases the frequency of Cl channel opening
   3. GABA_B_ receptor: increases the duration of Cl channel opening
   4. GABA_B_ receptor: increases the frequency of Cl channel opening
2. A 25-year-old man presents to the operating room for an appendectomy for acute appendicitis. The anesthesiologist measures the patient’s train-of-four at baseline, represented as (A) in the image below. After induction of general anesthesia, the train-of-four is measured again, represented as (B) in the image. Following completion of surgery, drug X is administered to the patient. A few minutes after administration of this drug, the train-of-four is measured again, represented as (C) in the image. Which medication of the options listed below likely represents drug X?


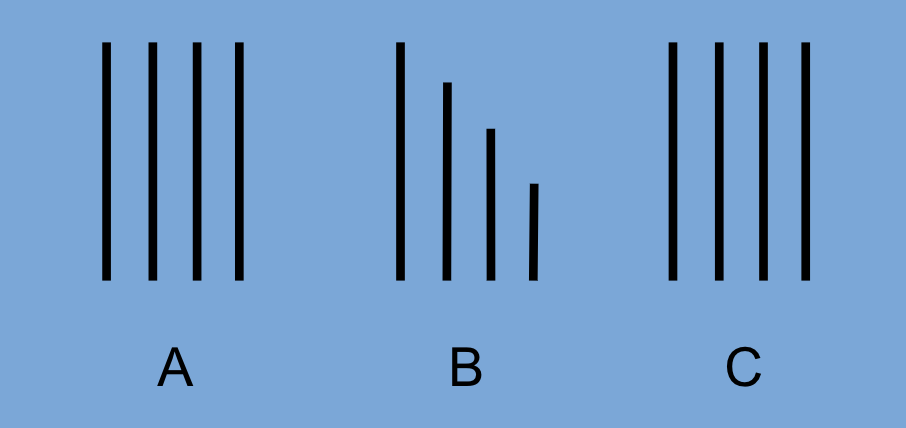


- 1. Desflurane
  2. Rocuronium
  3. Flumazenil
  4. Neostigmine

1. Prolonged exposure to which of the following anesthetics is associated with the potential development of vitamin B12 deficiency and megaloblastic anemia?
   1. Ketamine
   2. Isoflurane
   3. Nitrous Oxide
   4. Etomidate
2. Which of the following local anesthetics is metabolized by plasma cholinesterases?
   1. Procaine
   2. Bupivacaine
   3. Lidocaine
   4. Mepivacaine
3. Four volatile anesthetics are listed below with their respective minimum alveolar concentration (MAC). Which of the following agents has the lowest potency?
   1. Desflurane (MAC 6%)
   2. Sevoflurane (MAC 2%)
   3. Isoflurane (MAC 1%)
   4. Halothane (0.75%)

**Pharmacology of Sedating and Anesthetic Agents – Readiness Quiz Questions with Answers and Explanations**

1. A 55-year-old woman arrives to the hospital prior to undergoing spine surgery for chronic lower back pain. She states she is very nervous about the upcoming procedure. The anesthesiologist administers midazolam for pre-procedural anxiolysis. Which of the following describes the receptor and mechanism of this agent?

1. GABA_A_ receptor: increases the duration of Cl channel opening
2. **GABA_A_ receptor: increases the frequency of Cl channel opening**
3. GABA_B_ receptor: increases the duration of Cl channel opening
4. GABA_B_ receptor: increases the frequency of Cl channel opening

**Answer:** B

**Rationale**: Midazolam belongs to the benzodiazepine class of drugs, which bind to GABA_A_ receptors. Midazolam binds to an allosteric site of the receptor and modulates the endogenous signaling by increasing the frequency of channel opening.

2. A 25-year-old man presents to the operating room for an appendectomy for acute appendicitis. The anesthesiologist measures the patient’s train-of-four at baseline, represented as (A) in the image below. After induction of general anesthesia, the train-of-four is measured again, represented as (B) in the image. Following completion of surgery, drug X is administered to the patient. A few minutes after administration of this drug, the train-of-four is measured again, represented as (C) in the image. Which medication of the options listed below likely represents drug X?


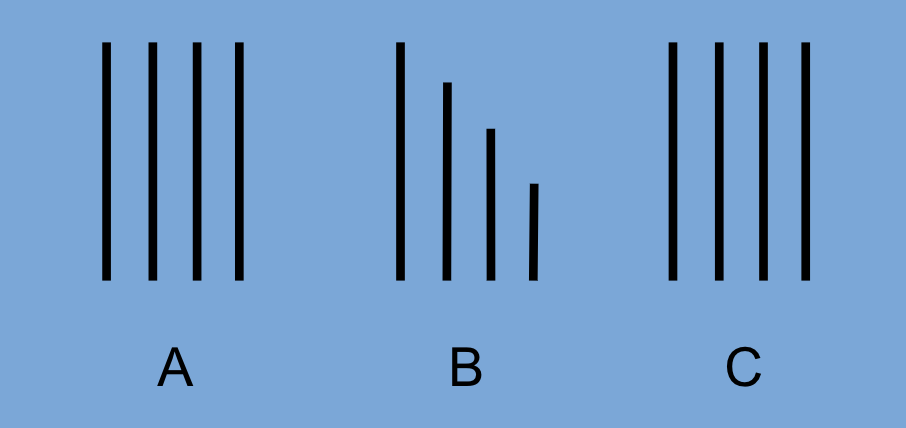


1. Desflurane
2. Rocuronium
3. Flumazenil
4. Neostigmine

**Answer:** D

**Rationale**: The change observed between figure (A) to (B) is consistent with administration of a non-depolarizing neuromuscular blocking agent (e.g. rocuronium) as demonstrated by the observed fade. Moreover, only non-depolarizing agents can be reversed, further supporting that a non-depolarizing agent was administered with induction of anesthesia. To reverse these agents, cholinesterase inhibitors (e.g. neostigmine) are administered as they inactivate acetylcholinesterase indirectly increasing the amount of acetylcholine available to compete with the non-depolarizing muscle relaxant. Alternatively, the selective relaxant binding agent sugammadex could have also been considered a correct answer to this question but was not provided in the list of available options.

3. Prolonged exposure to which of the following anesthetics is associated with the potential development of vitamin B12 deficiency and megaloblastic anemia?

1. Ketamine
2. Isoflurane
3. Nitrous Oxide
4. Etomidate

**Answer:** C

**Rationale**: Nitrous oxide inhibits the enzyme methionine synthase, which regulates vitamin B12 and folate metabolism and could cause megaloblastic anemia, vitamin B12 deficiency, and/or birth defects with chronic or prolonged exposure.

4. Which of the following local anesthetics is metabolized by plasma cholinesterases?

1. Procaine
2. Bupivacaine
3. Lidocaine
4. Mepivacaine

**Answer:** A

**Rationale**: Procaine is one of the ester-linked local anesthetics, which undergo metabolism by plasma cholinesterases. All other listed options are amide-linked local anesthetics, which undergo hepatic metabolism.

5. Four volatile anesthetics are listed below with their respective minimum alveolar concentration (MAC). Which of the following agents has the lowest potency?

1. Desflurane (MAC 6%)
2. Sevoflurane (MAC 2%)
3. Isoflurane (MAC 1%)
4. Halothane (0.75%)

**Answer:** A

**Rationale**: Minimum alveolar concentration (MAC) is defined as the end-tidal concentration (i.e. concentration measured upon exhalation) of inhaled anesthetic agent required to prevent movement in 50% of patients upon surgical stimulation. It is a measure of the anesthetic potency of the agent. MAC is small for potent anesthetics and larger for less potent agents. In this question, desflurane has a MAC of 6%, which is the largest MAC of the four agents listed indicating that it is the least potent.
