# Supplementary material for: Pharmacology of Sedating and Anesthetic Agents: A Case-Based Flipped Classroom Exercise for Preclinical Medical Students
Source: MedEdPORTAL. 2024 Nov 8;20:11462. doi: 10.15766/mep_2374-8265.11462 (PMC11543632; doi:10.15766/mep_2374-8265.11462)
Supplement: Supplementary file 1 — Study Guide.docxPresession Readiness Quiz.docxIn-Class Student Worksheet.docxClinical Case Slides.pptxFacilitator Guide.docxPostsession Consolidation Quiz.docxPostsession Satisfaction Survey.docx [file mep_2374-8265.11462-s001.zip › C. In-Class Student Worksheet.docx]

# **Pharmacology of Sedating and Anesthetic Agents – In-Class Student Worksheet**

**Instructions:** One copy of this worksheet should be distributed to each student group at the start of the in-class session. The students in each group should work collectively to answer the questions contained in this worksheet as the clinical case unfolds over the course of the 2-hour session.

**Date:**

**Group #:**

**Clinical Case Part 1**

A 66-year-old woman with new-onset headaches was recently found to have a large 12mm anterior communicating artery cerebral aneurysm. She now presents to the operating room for endovascular cerebral aneurysm coiling, in which the neurosurgeon will insert a thin catheter into a groin artery, advance it endovascularly under x-ray guidance into the cerebral vascular circulation, and a coil will be deployed to block blood flow into the aneurysm.

The patient reports no other past medical history and has never previously had surgery. She does not take any medications at home, but reports having had an allergic reaction to procaine including tongue and throat swelling during a dental procedure. She does not smoke cigarettes and denies use of alcohol or recreational drugs. She reports having been adopted as a child and is unaware of any family history of anesthetic complications.

Pre-procedure vitals:

BP: 133/85 mmHg

HR: 96 bpm

RR: 18 bpm

SpO2: 100% on room air

Physical exam is otherwise unremarkable. She states her blood pressure is usually lower at home and thinks it may be higher today because she is feeling “very nervous.”

After positioning on the procedure table and connecting to standard monitors, the neurosurgeon injects lidocaine subcutaneously in the right groin and the anesthesiologist administer midazolam intravenously. Upon initial skin puncture, the patient reports sharp discomfort at the groin surgical site and additional lidocaine is injected with relief of pain. Fifteen minutes after starting the procedure, the patient complains of tingling of her lips (perioral numbness), ringing in her ears (tinnitus), and shortly thereafter loses consciousness and experiences tonic-clonic convulsions.

You are the medical student assigned to the neurosurgeon and anesthesiologist that day and to answer any questions they may have for you.

**Application Exercise #1 (6 minutes)**

**Q1:**

1. **Why was midazolam used? (What was the anesthesiologist trying to achieve by using this agent?)**
2. **What is the mechanism of action of midazolam? (Please fill in the blank activity to explain your answer.)**

*The answer should explain the following characteristics once completed:*

*Class of drug, target receptor, site of receptor binding, agonism/antagonism, type of ion(s) involved, and physiologic process affected by midazolam.*

Midazolam belongs to the _____________ class of drugs. It binds to _______ receptors.

Endogenous ligand ______, the major (inhibitory/excitatory) neurotransmitter in the CNS binds to the same receptor. Binding of endogenous ligand to this receptor (opens/closes) the ion channel allowing _______ ions to pass (into/out of) the nerve cell. This causes (hyperpolarization/depolarization) of the neuron and (increase/decrease) neurotransmission by (inhibiting/promoting) the formation of an action potential.

Midazolam binds to an (allosteric/active) site of the same receptor. Midazolam modulates the endogenous signaling by (increasing/decreasing) the (frequency/duration) of channel opening. Midazolam is only able to work in the (presence/absence) of the endogenous ligand.

**Q2:**

1. **Why was lidocaine used? (What was the surgeon trying to achieve by using this agent?)**
2. **What is the mechanism of action of lidocaine?**

*The answer should explain the following characteristics once completed:*

*Class and subclass of drug, target receptor, how it enters the cell and where it binds, agonism/antagonism, type of ion(s) involved, and physiologic process affected by lidocaine.*

Lidocaine is a local anesthetic belonging to the sub class of (Esther/Amide) linked local anesthetics. Amide linked have (one/two) “i” in their name, while Esther linked local anesthetics have (one/two) ‘i’ in their name 😊!.

(Ionized/Non ionized) lidocaine diffuses through the cell membrane. (Ionized/Non ionized) lidocaine binds to the cytoplasmic side of the ________ - gated _____ ion channel and (activates/blocks) it. This prevents a transient (increase/decrease) in permeability of the nerve membrane to ___ ions, which is required for generation and propagation of an action potential. When propagation of action potentials is prevented, sensory impulses cannot be transmitted from the __________ to the ________.

1. **Complete the table for local anesthetics:**

|  | **Esters-linked** | **Amide-linked** |
| --- | --- | --- |
| **Drug names** |  |  |
| **Systemic toxicity** | (more/less) likely | (more/less) likely |
| **Metabolism** | (rapid/slow) by ____________ | (rapid/slow) by ____________ |
| **Allergic reaction** | (more/less) likely | (more/less) likely |

1. **What properties of this agent make it a specifically good choice for this patient?**
2. **Why do you think the patient experienced perioral numbness, tinnitus, and a seizure?**
3. **What can be done to treat this condition? How could this have been prevented?**

**Clinical Case Part 2**

**Application Exercise #2 (7 minutes)**

**Q3:**

1. **Why are each of the following medications used when inducing general anesthesia? (What was the anesthesiologist trying to achieve by using these agents?)**
   1. **Fentanyl**
   2. **Propofol**
   3. **Succinylcholine**

**Q4:**

1. **What are the potential adverse reactions of propofol that the anesthesiologist must consider in this short-term setting?**
2. **What are the potential adverse reactions if propofol were to be used for sedation over several days?**

**Q5:**

1. **How does the mechanism of action differ between the muscle relaxants succinylcholine and rocuronium?**

Neuro Muscular Junctions (NMJs) have nicotinic/muscarinic ACh receptors. These receptors are ______-gated ion channels that open in response to binding of ACh (endogenous ligand) to mediate influx of ____ ions into the muscle cell. ___ ion influx dominates the exchange, and the membrane _________, a response known as a motor endplate potential. Therefore, ACh binding to these receptors initiates muscle __________. Endogenous ACh is quickly degraded by ______________ in the synaptic cleft resulting in termination of its action and depolarization/repolarization of the membrane.

Succinylcholine structurally resembles the ____ molecule and acts as a nicotinic ACh receptor agonist/antagonist. Succinylcholine is ________to degradation by _____ in the synaptic cleft, which allows it to persist and continue stimulating the receptor. This first causes opening of the ______ channel associated with nicotinic receptors, which results in depolarization/repolarization (phase I). This leads to a transient twitching of the muscle known as _____________.

Continued binding of succinylcholine to the receptor causes the receptor to become sensitized/desensitized and renders the receptor incapable/capable of transmitting further impulses. Succinylcholine belongs to the group of drugs called Depolarizing/Non–depolarization muscle relaxants and is often used to facilitate endotracheal tube insertion. Its effects are reasonably short lived because of degradation by __________________.

Rocuronium is a competitive agonist/antagonist at the nicotinic ACh receptors. It competes with endogenous ligand ____ at the receptor and promotes/prevents its binding to its receptor. This prevents depolarization/repolarization of the muscle cell membrane and inhibits development of an end plate potential therefore preventing muscular contraction. Its competitive action can be overcome by administration of ____________, such as neostigmine, which increase the concentration of ACh in the NMJ. Clinicians employ this strategy to shorten/prolong or “reverse” the duration of neuromuscular blockade. Agents such as rocuronium are Depolarizing/Non-depolarizing muscle relaxants.

1. **What are the potential adverse reactions of succinylcholine that the anesthesiologist must consider? What patient characteristics (not present in this patient) would have contraindicated its use?**

**Clinical Case Part 3**

**Application Exercise #3 (7 minutes)**

**Q6:**

1. **How is a train-of-four response evaluated? Why did the anesthesiologist wish to evaluate it?**
2. **How would the train-of-four response have differed if rocuronium had been given instead of succinylcholine?**

**Q7:**

1. **Will this patient require reversal of neuromuscular blockade? Why or why not?**
2. **Would your answer change if the patient had received rocuronium instead of succinylcholine?**
3. **Complete the table below for the two classes of medications used to reverse neuromuscular blockage by non-depolarizing neuromuscular blocking agent.**

|  | **Cholinesterase Inhibitors** | **Selective Relaxant Binding Agent** |
| --- | --- | --- |
| **Drug names (generic)** |  |  |
| **Muscle relaxants reversed by this class of drugs** |  |  |
| **Mechanism** |  |  |
| **Adverse reactions** |  |  |

**Clinical Case Part 4**

**Application Exercise #4 (7 minutes)**

**Q8:**

1. **What is sevoflurane and what are the properties that characterize inhaled anesthetics? (Please complete the fill in the blank activity to explain your answer.)**

Sevoflurane belongs to the (volatile/gaseous) class of inhaled anesthetics. The potency of an inhaled anesthetic is characterized by the unit referred to as the _______________, which is the concentration of anesthetic agent exhaled by the patient required to prevent movement upon surgical stimulation in 50% of patients. According to this principle, sevoflurane is (more/less) potent than nitrous oxide. The time to onset and offset of an inhaled anesthetic is determined by its _________ in blood, measured by the _________________. According to this principle, sevoflurane would be expected to have a (slower/faster) onset than isoflurane.

**Q9:**

1. **Why do you think the patient experienced tachycardia, increased CO2 exhalation, and muscle rigidity?**
2. **What agent(s) may have triggered these symptoms? Why?**
3. **How should this condition be treated? What is the mechanism of this intervention?**
