# Supplementary material for: Pharmacology of Sedating and Anesthetic Agents: A Case-Based Flipped Classroom Exercise for Preclinical Medical Students
Source: MedEdPORTAL. 2024 Nov 8;20:11462. doi: 10.15766/mep_2374-8265.11462 (PMC11543632; doi:10.15766/mep_2374-8265.11462)
Supplement: Supplementary file 1 — Study Guide.docxPresession Readiness Quiz.docxIn-Class Student Worksheet.docxClinical Case Slides.pptxFacilitator Guide.docxPostsession Consolidation Quiz.docxPostsession Satisfaction Survey.docx [file mep_2374-8265.11462-s001.zip › D. Clinical Case Slides.pptx]

## Slide 1
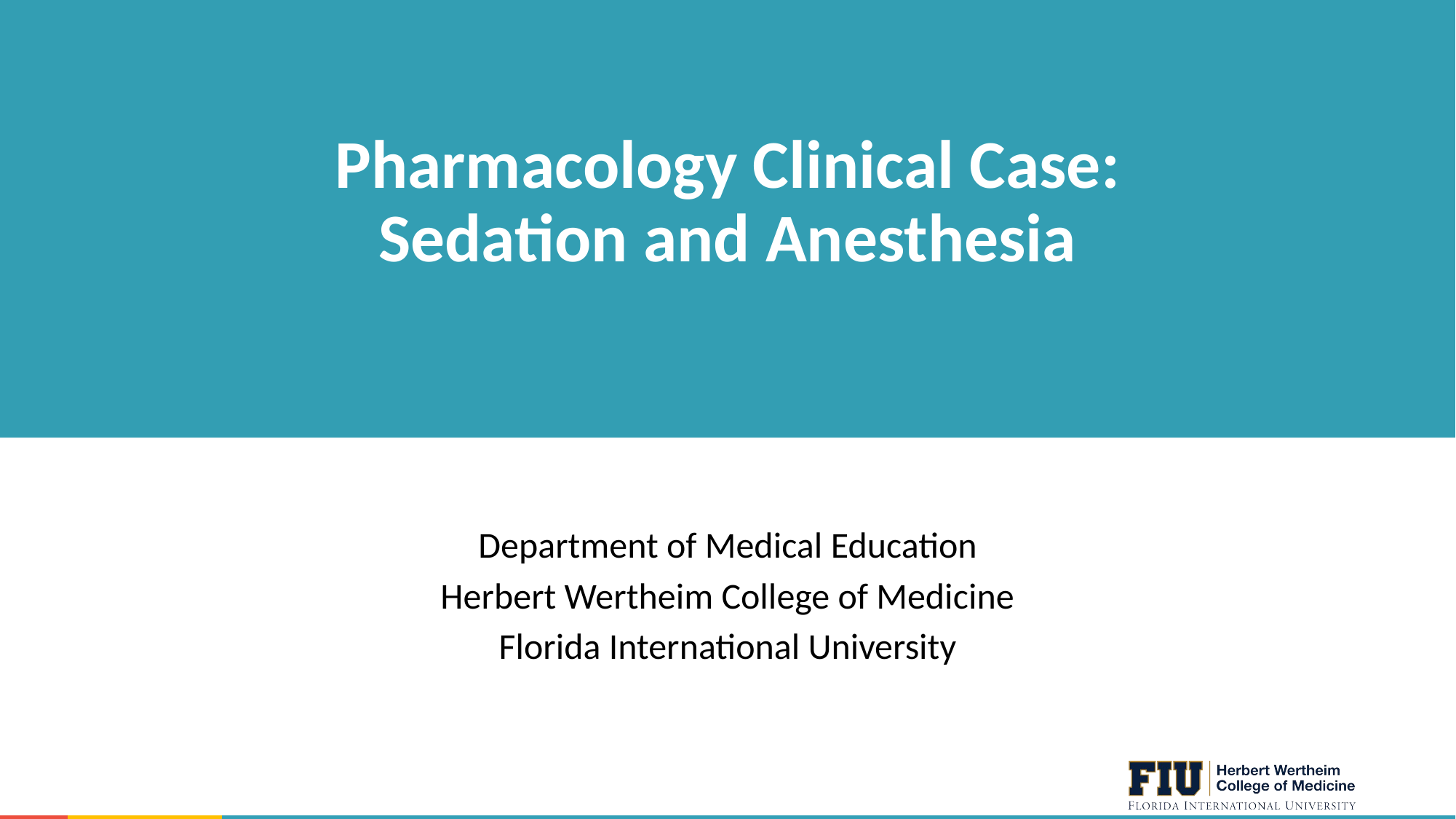

# Pharmacology Clinical Case: Sedation and Anesthesia
Department of Medical Education
Herbert Wertheim College of Medicine
Florida International University

## Slide 2
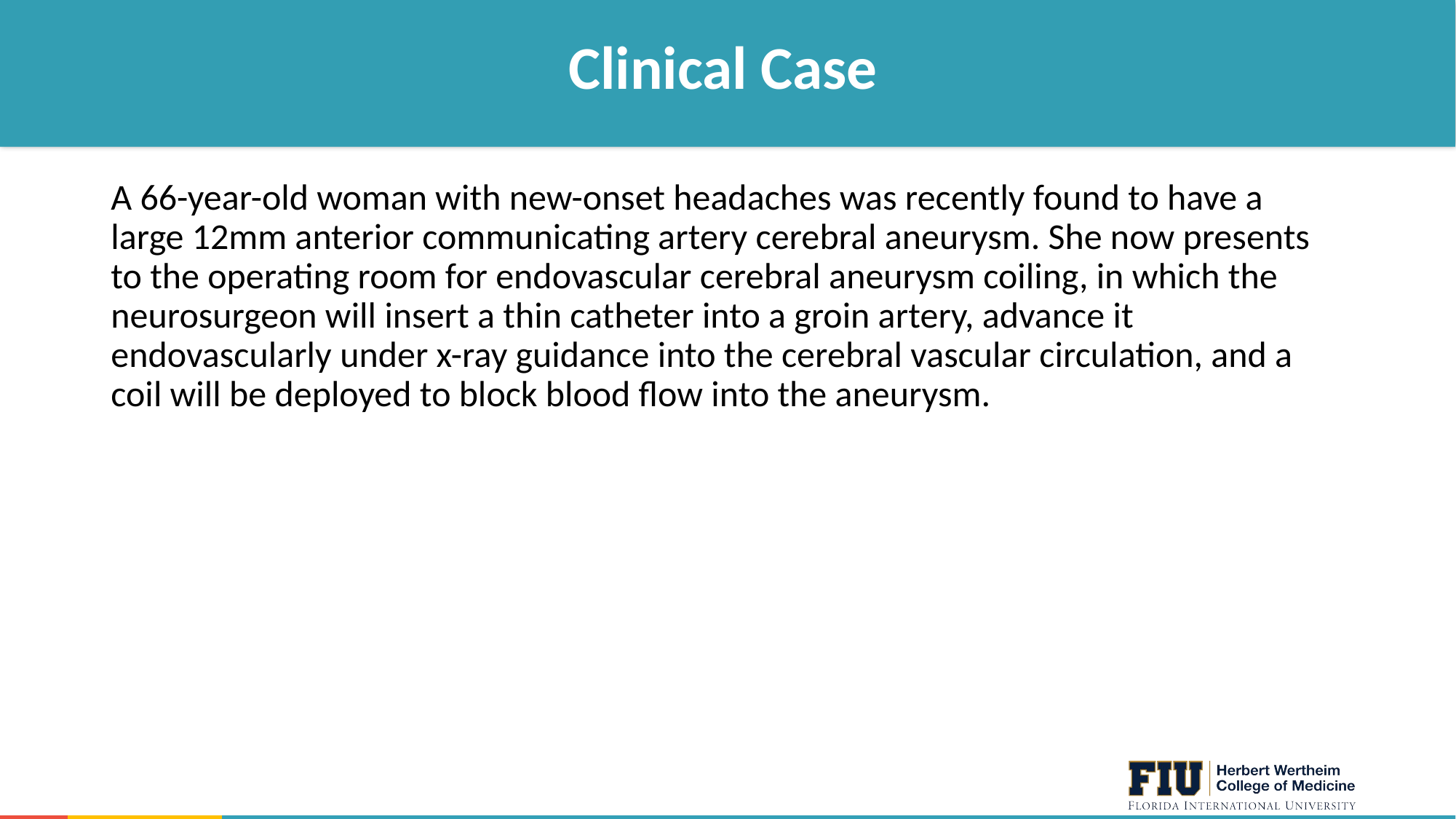

# Clinical Case
A 66-year-old woman with new-onset headaches was recently found to have a large 12mm anterior communicating artery cerebral aneurysm. She now presents to the operating room for endovascular cerebral aneurysm coiling, in which the neurosurgeon will insert a thin catheter into a groin artery, advance it endovascularly under x-ray guidance into the cerebral vascular circulation, and a coil will be deployed to block blood flow into the aneurysm.

## Slide 3
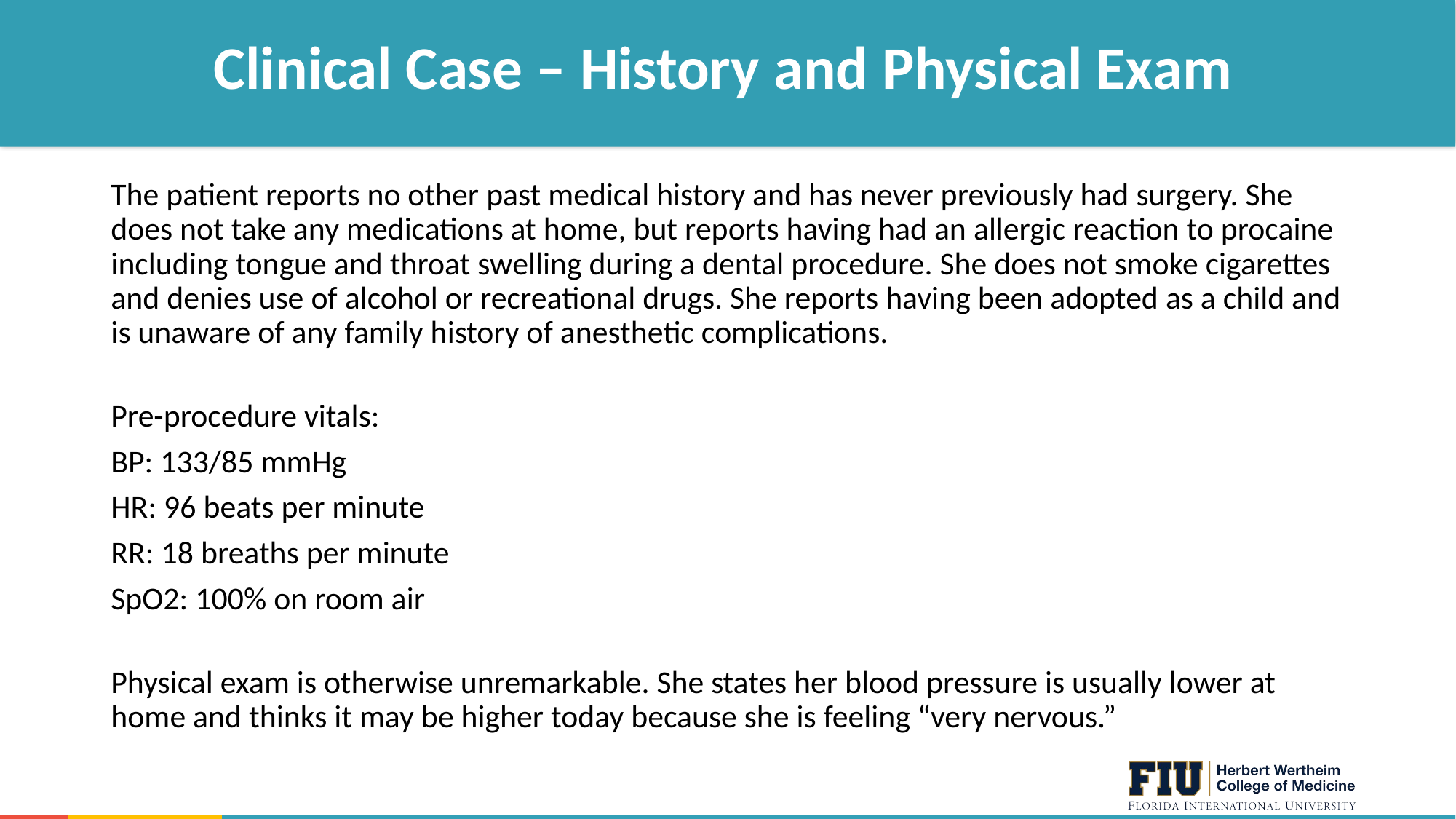

# Clinical Case – History and Physical Exam
The patient reports no other past medical history and has never previously had surgery. She does not take any medications at home, but reports having had an allergic reaction to procaine including tongue and throat swelling during a dental procedure. She does not smoke cigarettes and denies use of alcohol or recreational drugs. She reports having been adopted as a child and is unaware of any family history of anesthetic complications.
Pre-procedure vitals:
BP: 133/85 mmHg
HR: 96 beats per minute
RR: 18 breaths per minute
SpO2: 100% on room air
Physical exam is otherwise unremarkable. She states her blood pressure is usually lower at home and thinks it may be higher today because she is feeling “very nervous.”

## Slide 4
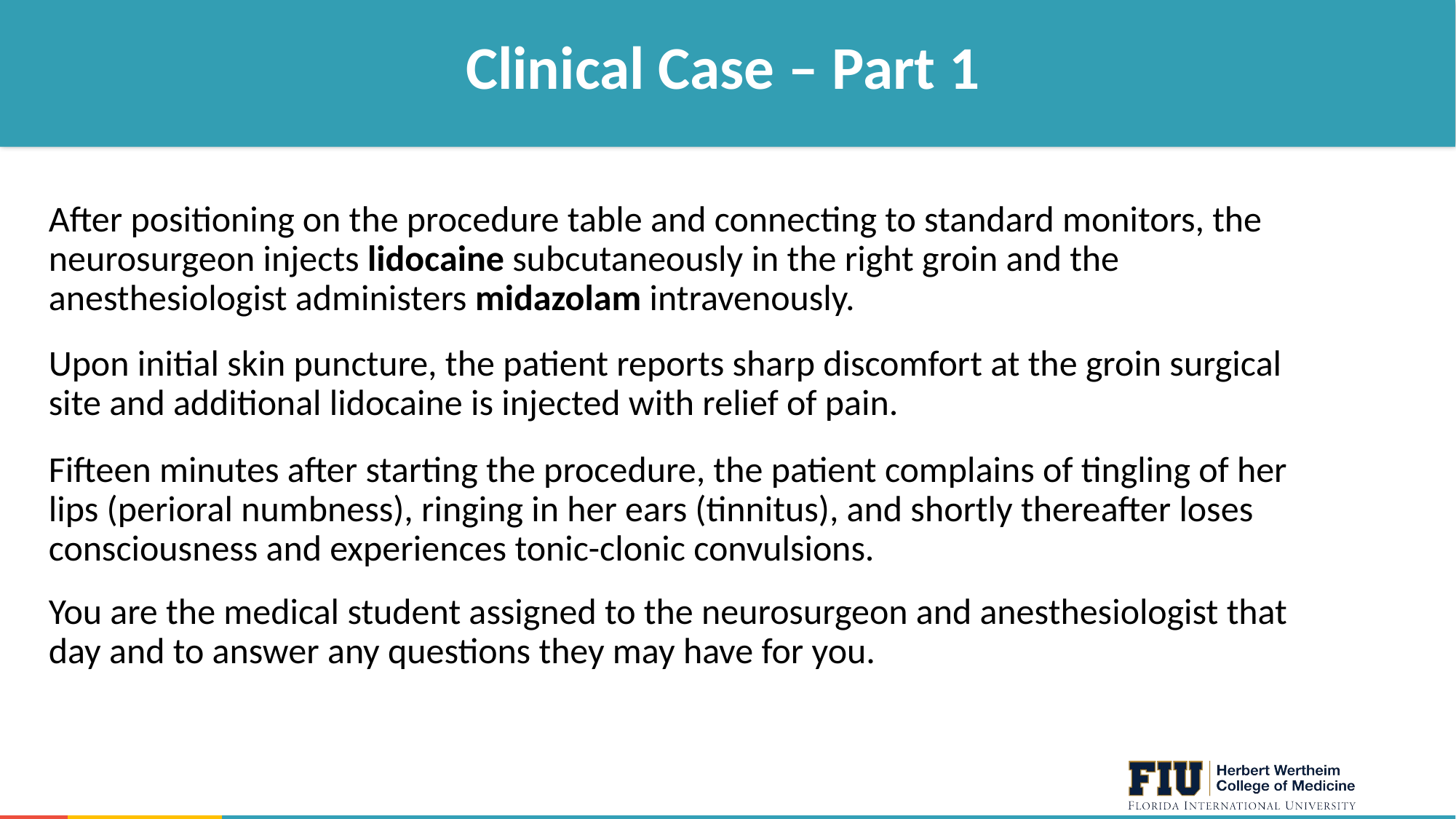

# Clinical Case – Part 1
After positioning on the procedure table and connecting to standard monitors, the neurosurgeon injects lidocaine subcutaneously in the right groin and the anesthesiologist administers midazolam intravenously.
Upon initial skin puncture, the patient reports sharp discomfort at the groin surgical site and additional lidocaine is injected with relief of pain.
Fifteen minutes after starting the procedure, the patient complains of tingling of her lips (perioral numbness), ringing in her ears (tinnitus), and shortly thereafter loses consciousness and experiences tonic-clonic convulsions.
You are the medical student assigned to the neurosurgeon and anesthesiologist that day and to answer any questions they may have for you.

## Slide 5
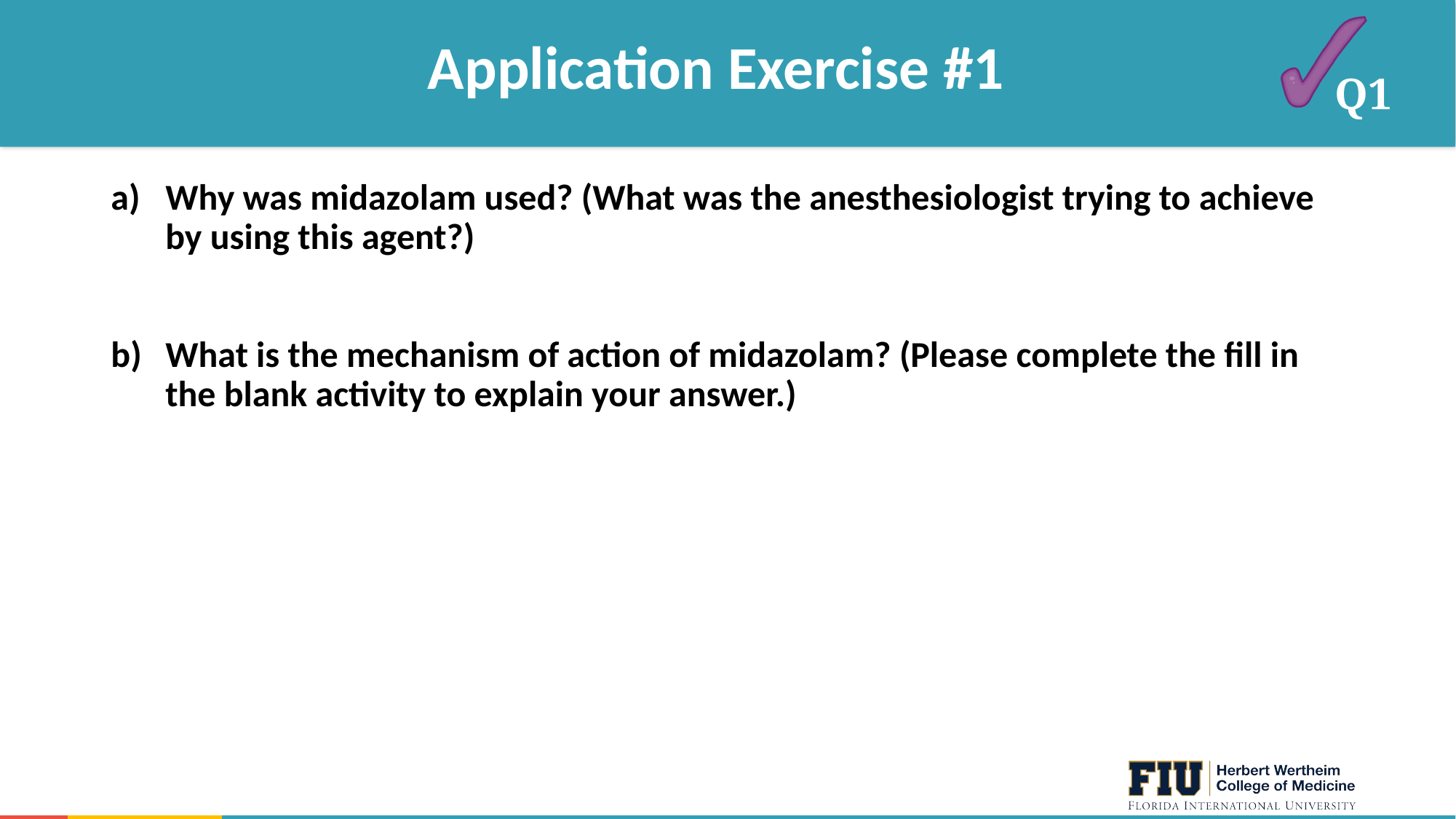

# Application Exercise #1
Q1
Why was midazolam used? (What was the anesthesiologist trying to achieve by using this agent?)
What is the mechanism of action of midazolam? (Please complete the fill in the blank activity to explain your answer.)

## Slide 6
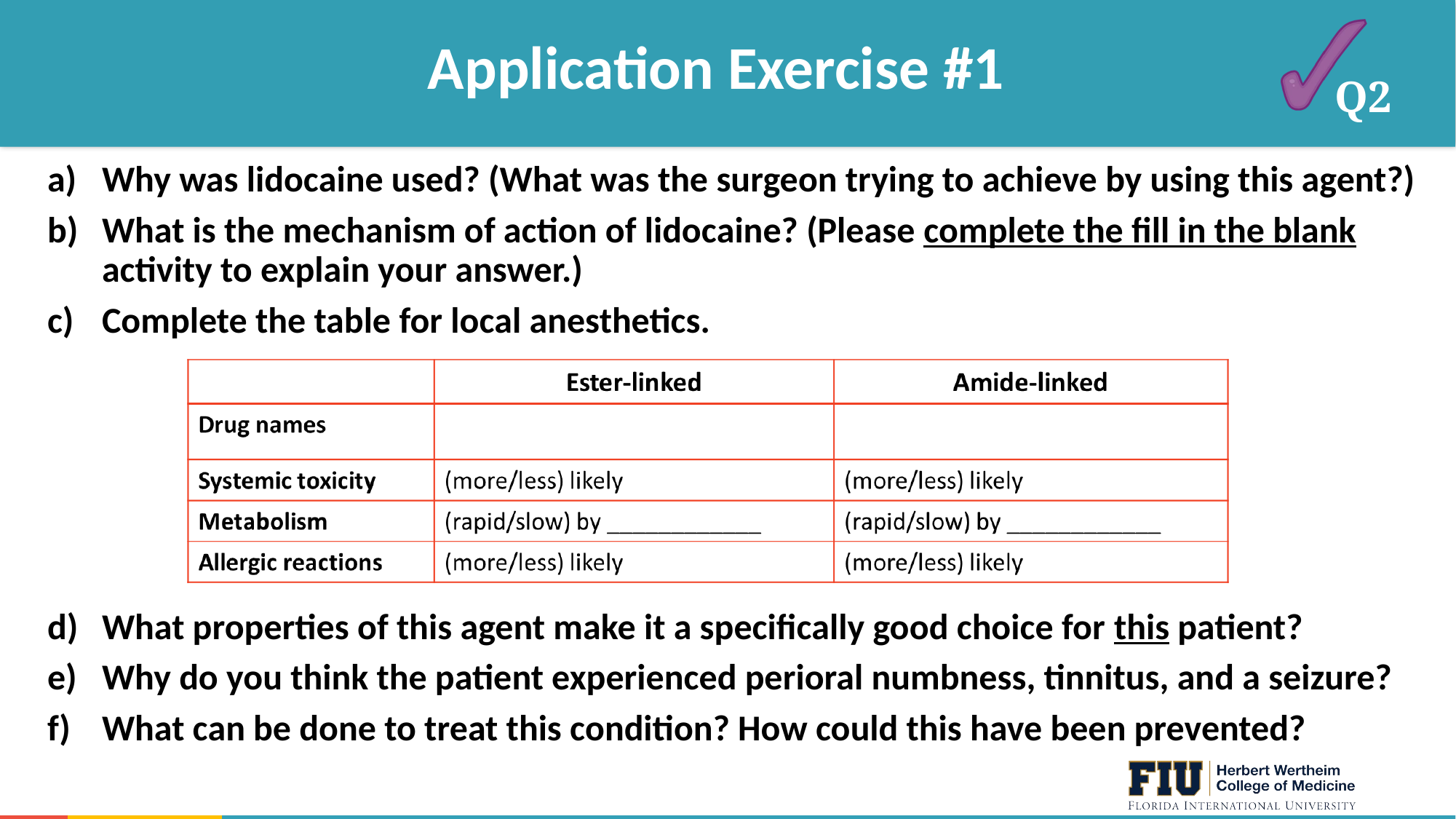

# Application Exercise #1
Q2
Why was lidocaine used? (What was the surgeon trying to achieve by using this agent?)
What is the mechanism of action of lidocaine? (Please complete the fill in the blank activity to explain your answer.)
Complete the table for local anesthetics.
What properties of this agent make it a specifically good choice for this patient?
Why do you think the patient experienced perioral numbness, tinnitus, and a seizure?
What can be done to treat this condition? How could this have been prevented?

## Slide 7
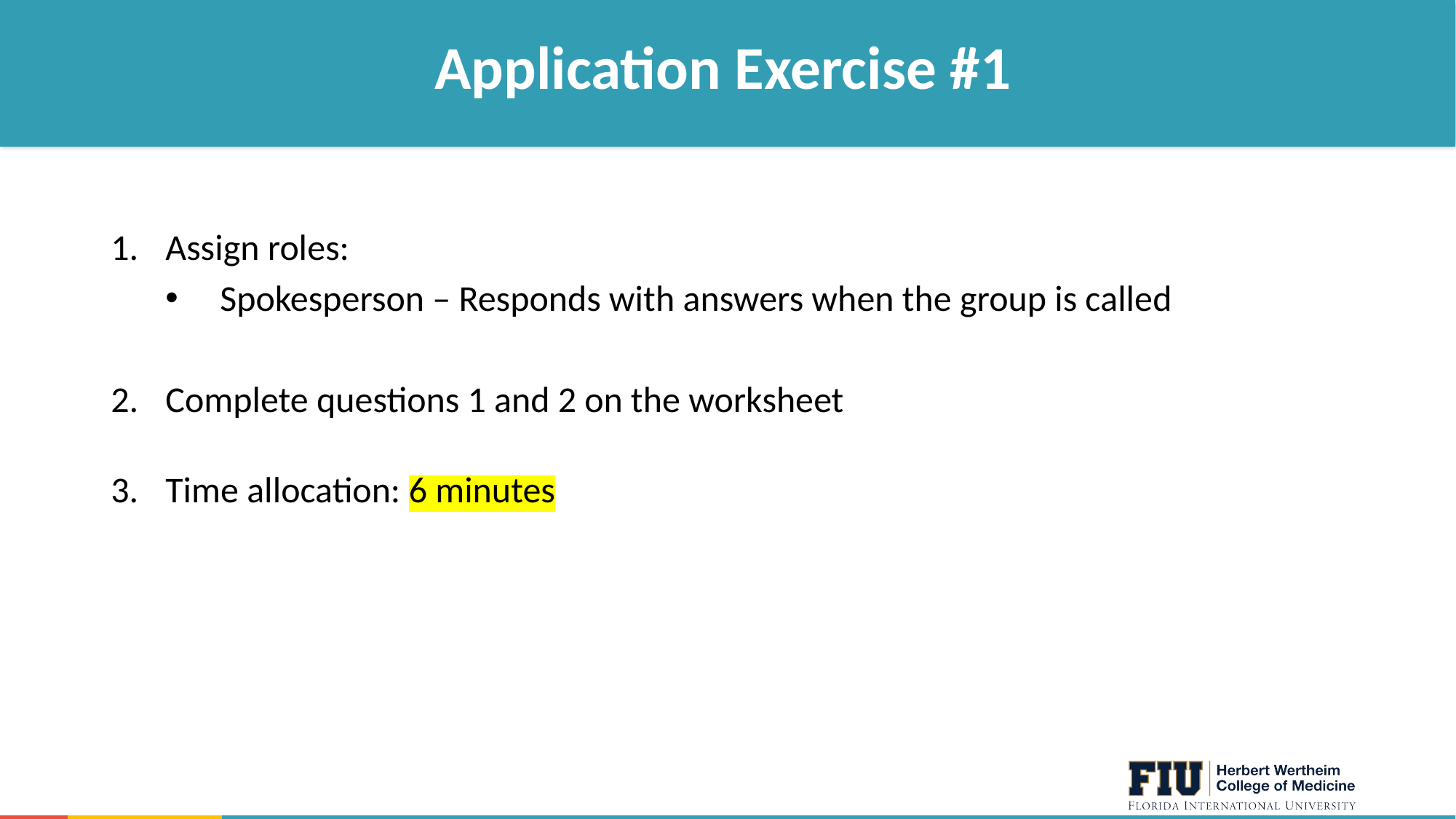

# Application Exercise #1
Assign roles:
Spokesperson – Responds with answers when the group is called
Complete questions 1 and 2 on the worksheet
Time allocation: 6 minutes

## Slide 8
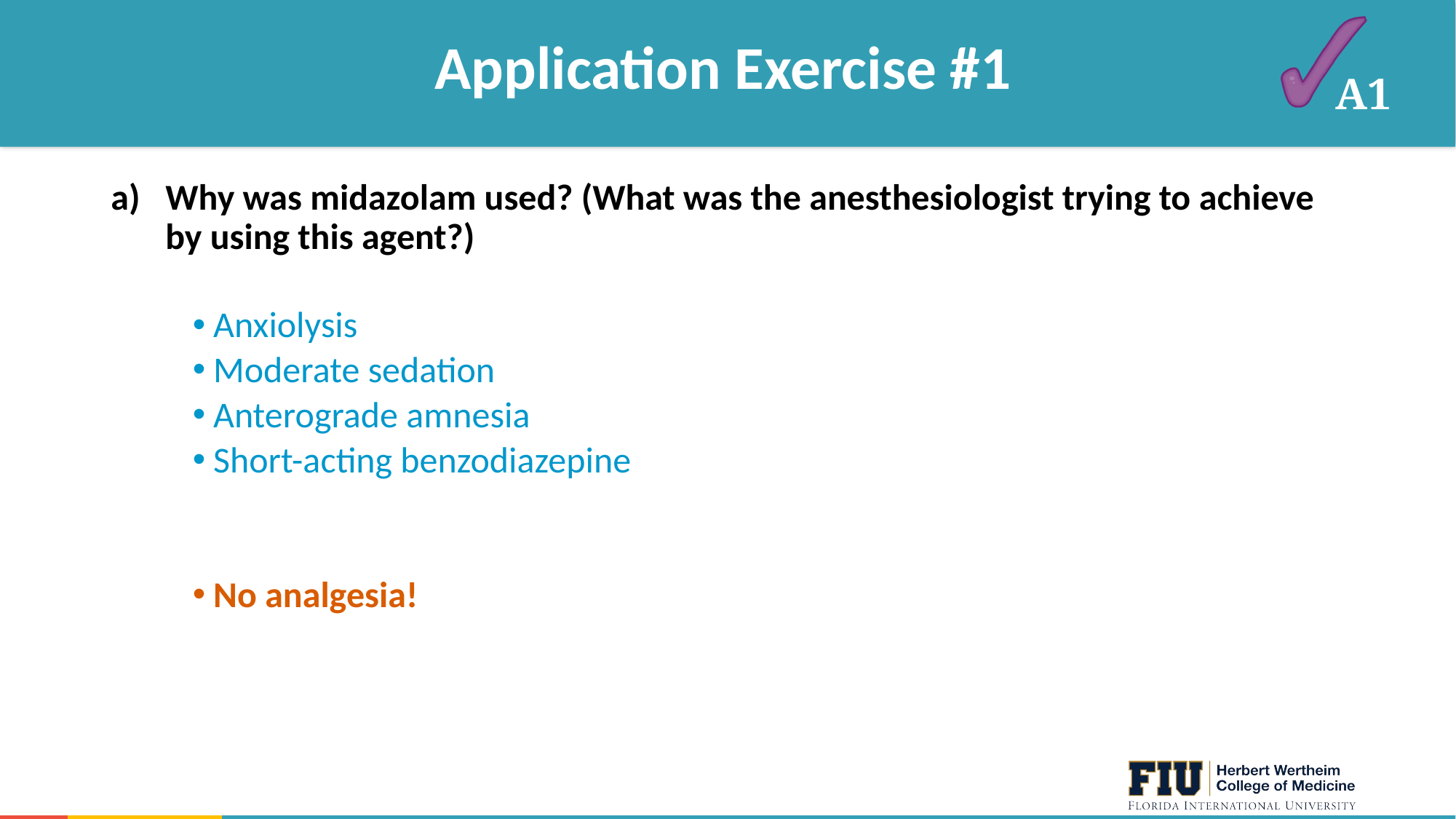

# Application Exercise #1
A1
Why was midazolam used? (What was the anesthesiologist trying to achieve by using this agent?)
Anxiolysis
Moderate sedation
Anterograde amnesia
Short-acting benzodiazepine
No analgesia!

## Slide 9
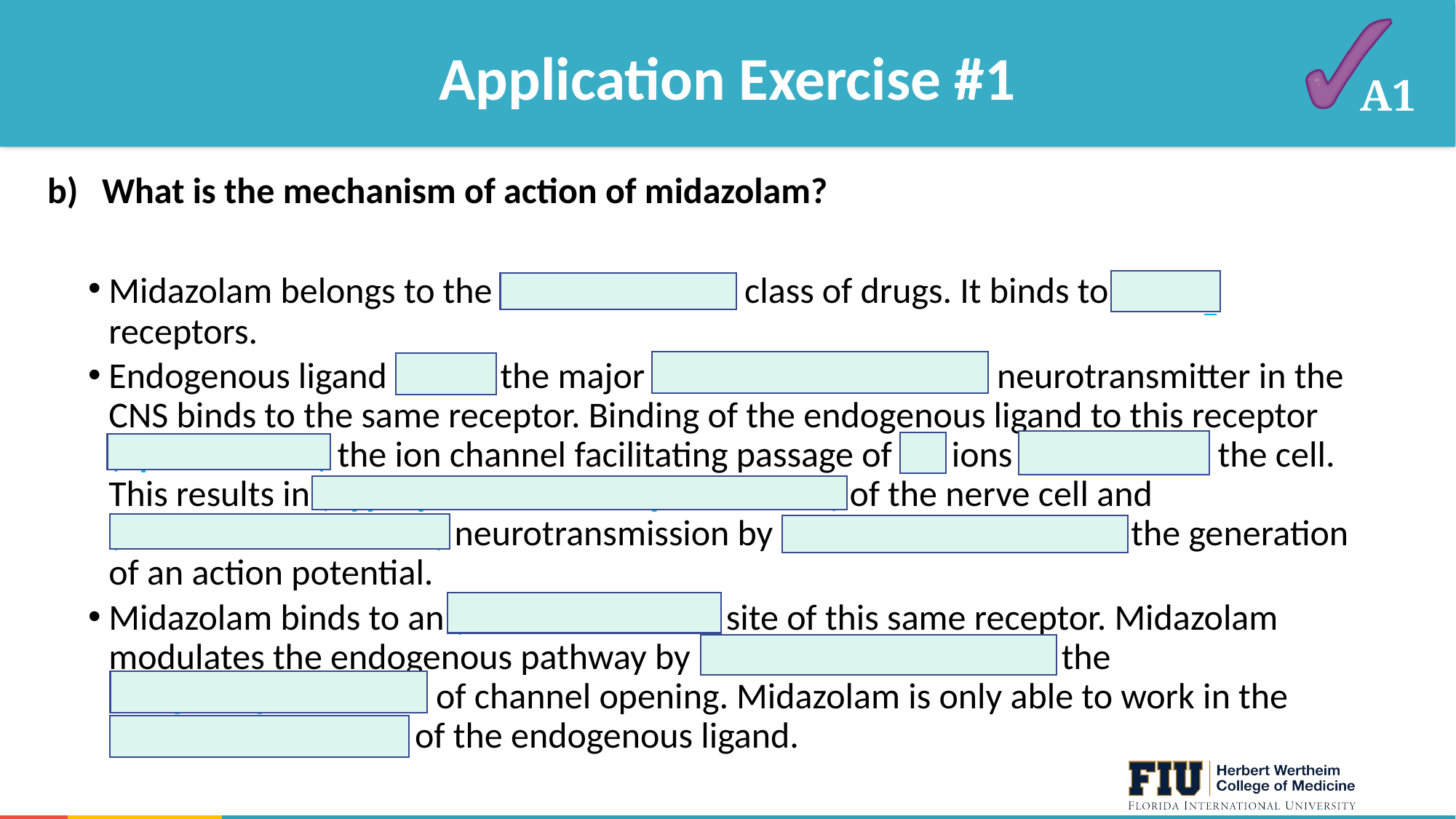

A1
# Application Exercise #1
What is the mechanism of action of midazolam?
Midazolam belongs to the benzodiazepine class of drugs. It binds to GABAA receptors.
Endogenous ligand GABA, the major (inhibitory/excitatory) neurotransmitter in the CNS binds to the same receptor. Binding of the endogenous ligand to this receptor (opens/closes) the ion channel facilitating passage of Cl- ions (into/out of) the cell. This results in (hyperpolarization/depolarization) of the nerve cell and (increased/decreased) neurotransmission by (inhibiting/promoting) the generation of an action potential.
Midazolam binds to an (allosteric/active) site of this same receptor. Midazolam modulates the endogenous pathway by (increasing/decreasing) the (frequency/duration) of channel opening. Midazolam is only able to work in the (presence/absence) of the endogenous ligand.

## Slide 10
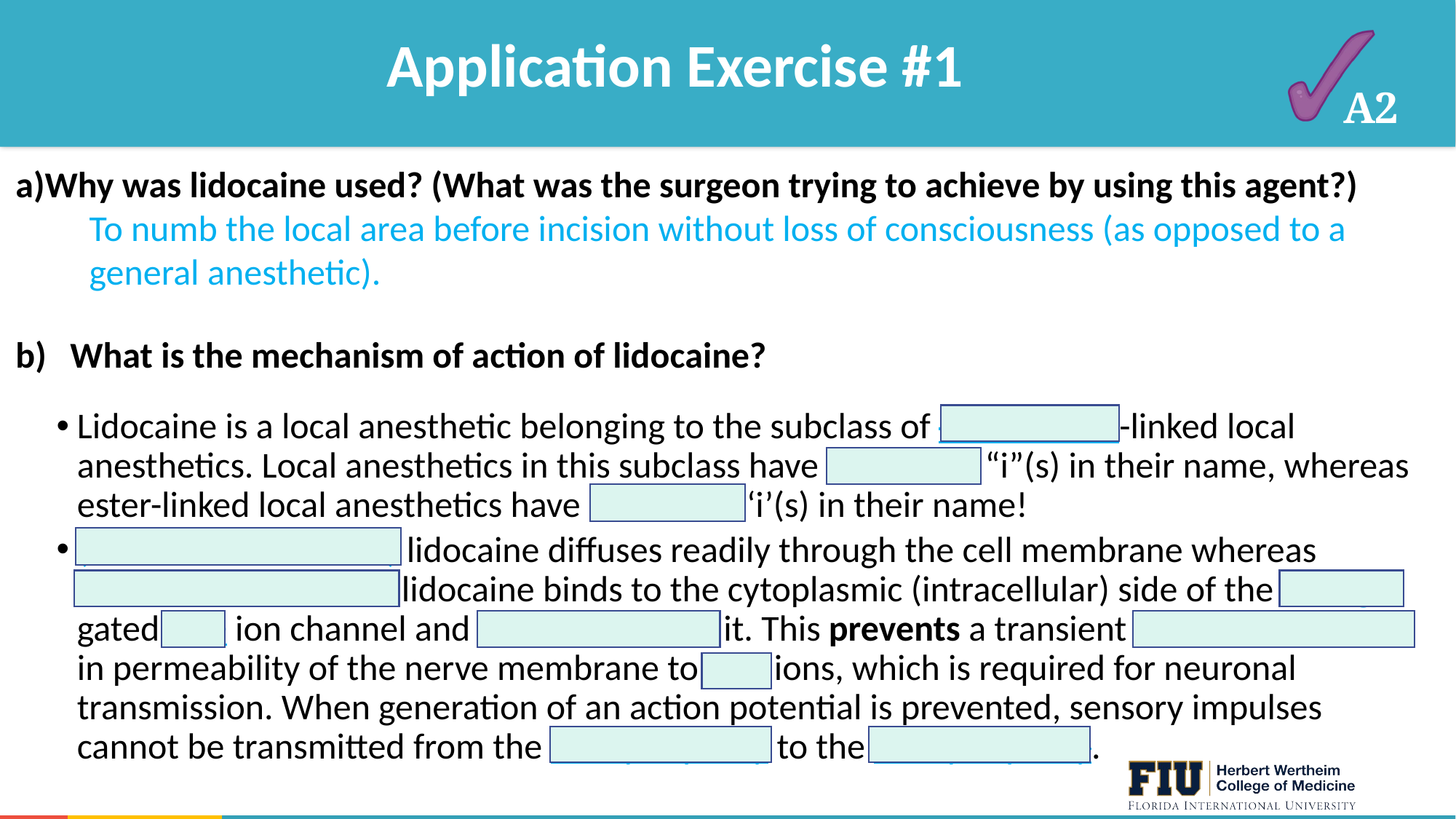

# Application Exercise #1
A2
Why was lidocaine used? (What was the surgeon trying to achieve by using this agent?)
 To numb the local area before incision without loss of consciousness (as opposed to a
 general anesthetic).
What is the mechanism of action of lidocaine?
Lidocaine is a local anesthetic belonging to the subclass of ester/amide-linked local anesthetics. Local anesthetics in this subclass have (one/two) “i”(s) in their name, whereas ester-linked local anesthetics have (one/two) ‘i’(s) in their name!
(Ionized/Non-ionized) lidocaine diffuses readily through the cell membrane whereas (ionized/non-ionized) lidocaine binds to the cytoplasmic (intracellular) side of the voltage-gated Na+ ion channel and activates/blocks it. This prevents a transient increase/decrease in permeability of the nerve membrane to Na+ ions, which is required for neuronal transmission. When generation of an action potential is prevented, sensory impulses cannot be transmitted from the CNS/periphery to the CNS/periphery.

## Slide 11
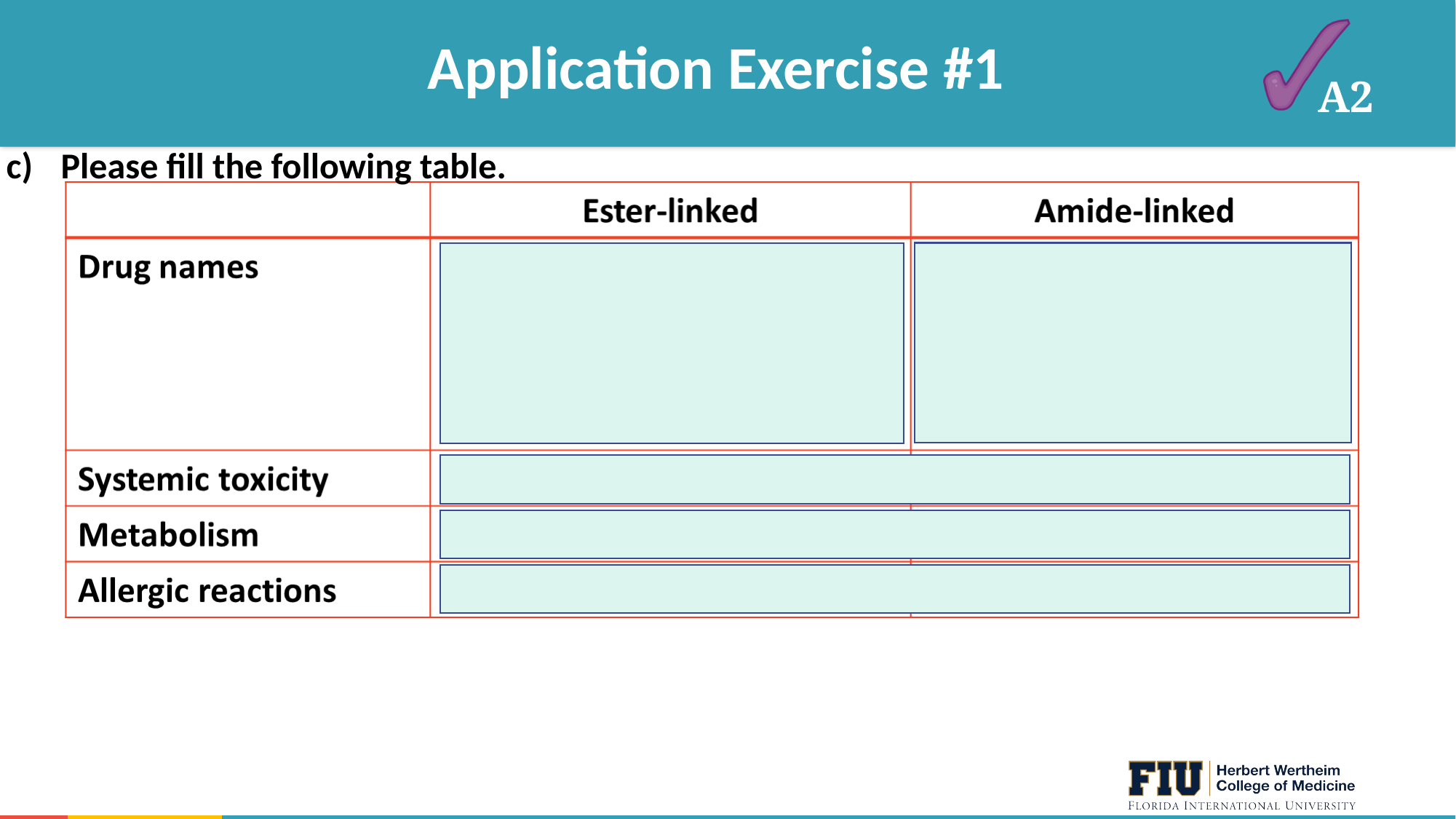

# Application Exercise #1
A2
Please fill the following table.
One “i”
Two “i”s

## Slide 12
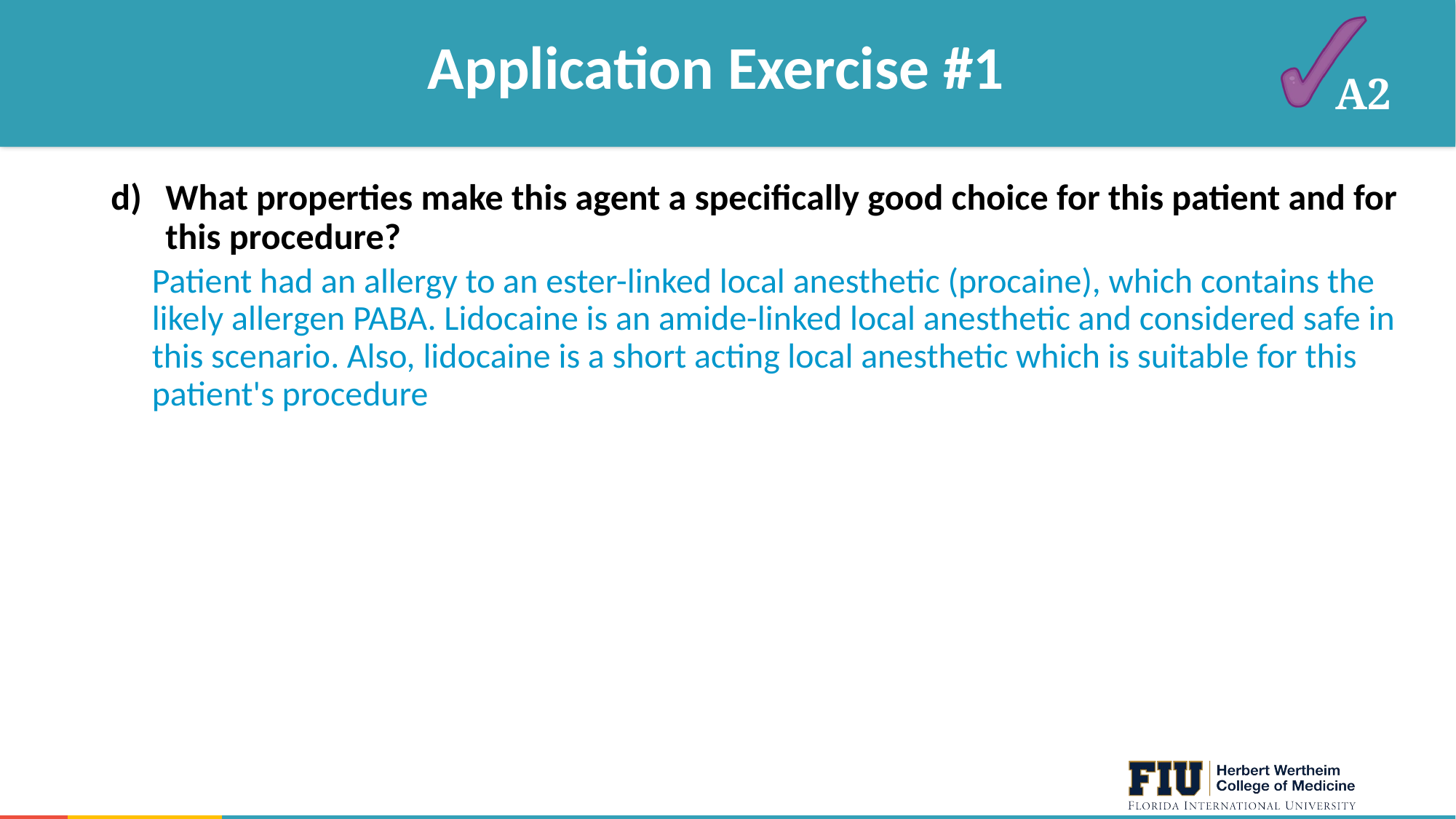

# Application Exercise #1
A2
What properties make this agent a specifically good choice for this patient and for this procedure?
Patient had an allergy to an ester-linked local anesthetic (procaine), which contains the likely allergen PABA. Lidocaine is an amide-linked local anesthetic and considered safe in this scenario. Also, lidocaine is a short acting local anesthetic which is suitable for this patient's procedure

## Slide 13
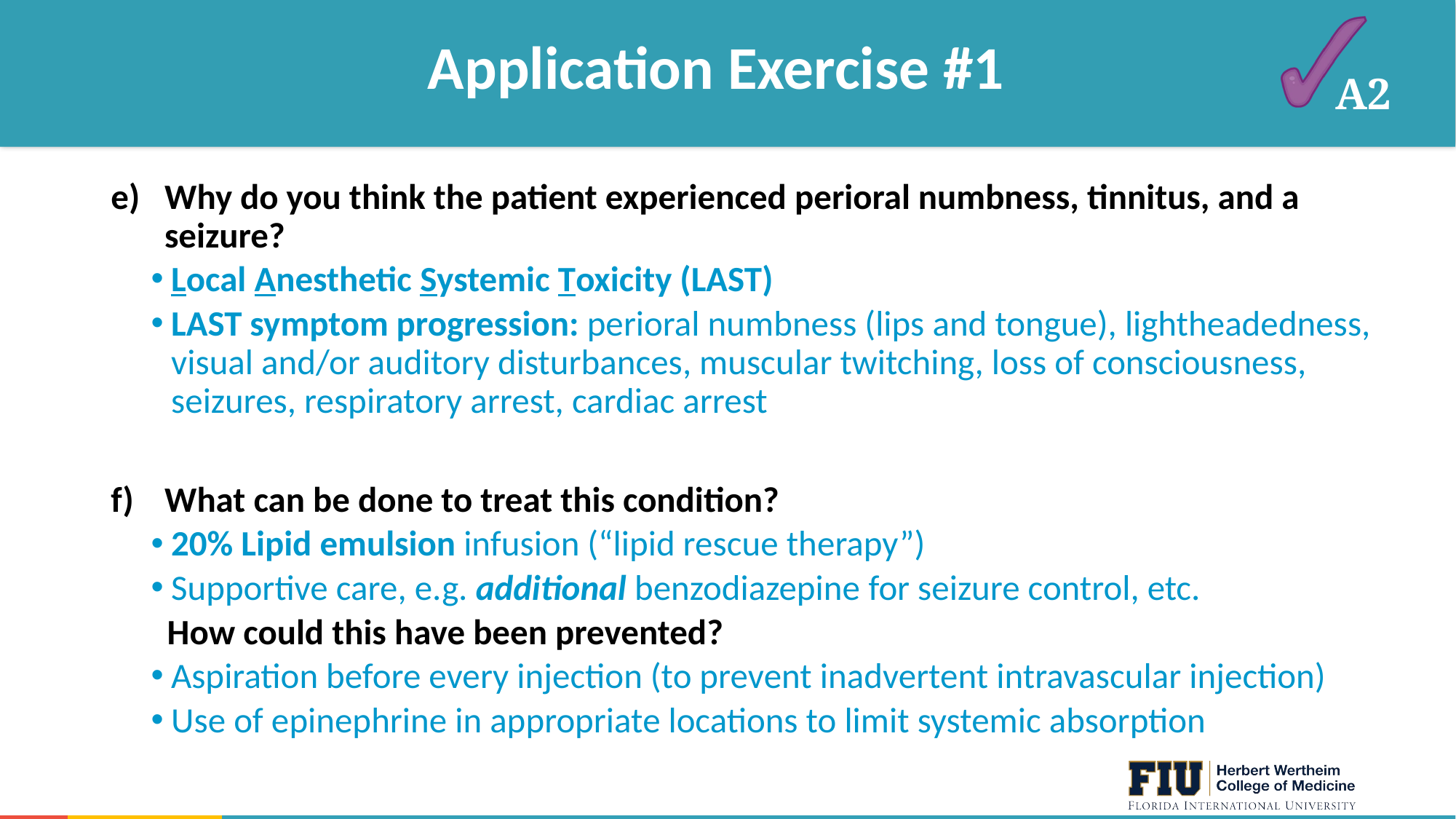

# Application Exercise #1
A2
Why do you think the patient experienced perioral numbness, tinnitus, and a seizure?
Local Anesthetic Systemic Toxicity (LAST)
LAST symptom progression: perioral numbness (lips and tongue), lightheadedness, visual and/or auditory disturbances, muscular twitching, loss of consciousness, seizures, respiratory arrest, cardiac arrest
What can be done to treat this condition?
20% Lipid emulsion infusion (“lipid rescue therapy”)
Supportive care, e.g. additional benzodiazepine for seizure control, etc.
 How could this have been prevented?
Aspiration before every injection (to prevent inadvertent intravascular injection)
Use of epinephrine in appropriate locations to limit systemic absorption

## Slide 14
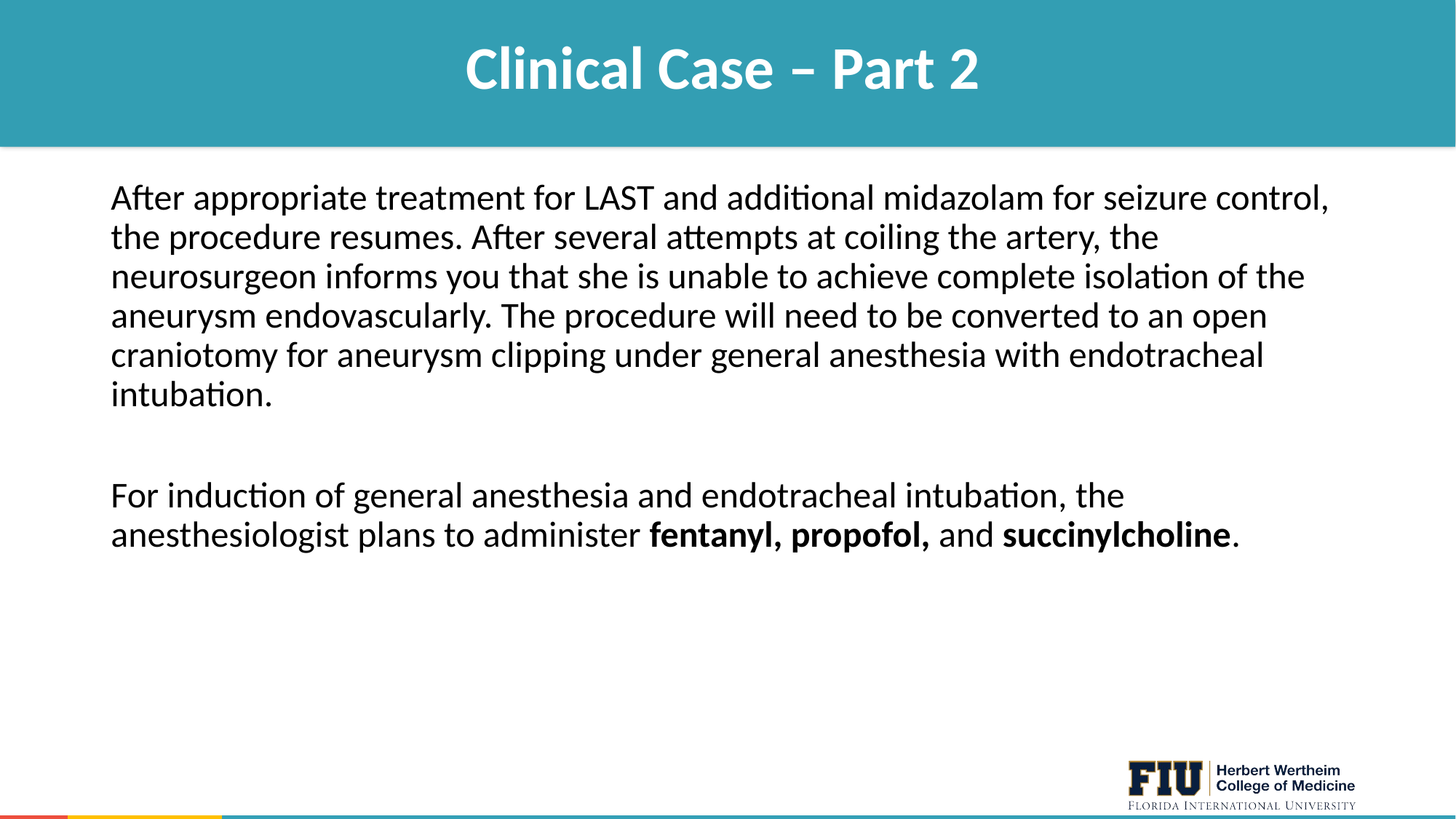

# Clinical Case – Part 2
After appropriate treatment for LAST and additional midazolam for seizure control, the procedure resumes. After several attempts at coiling the artery, the neurosurgeon informs you that she is unable to achieve complete isolation of the aneurysm endovascularly. The procedure will need to be converted to an open craniotomy for aneurysm clipping under general anesthesia with endotracheal intubation.
For induction of general anesthesia and endotracheal intubation, the anesthesiologist plans to administer fentanyl, propofol, and succinylcholine.

## Slide 15
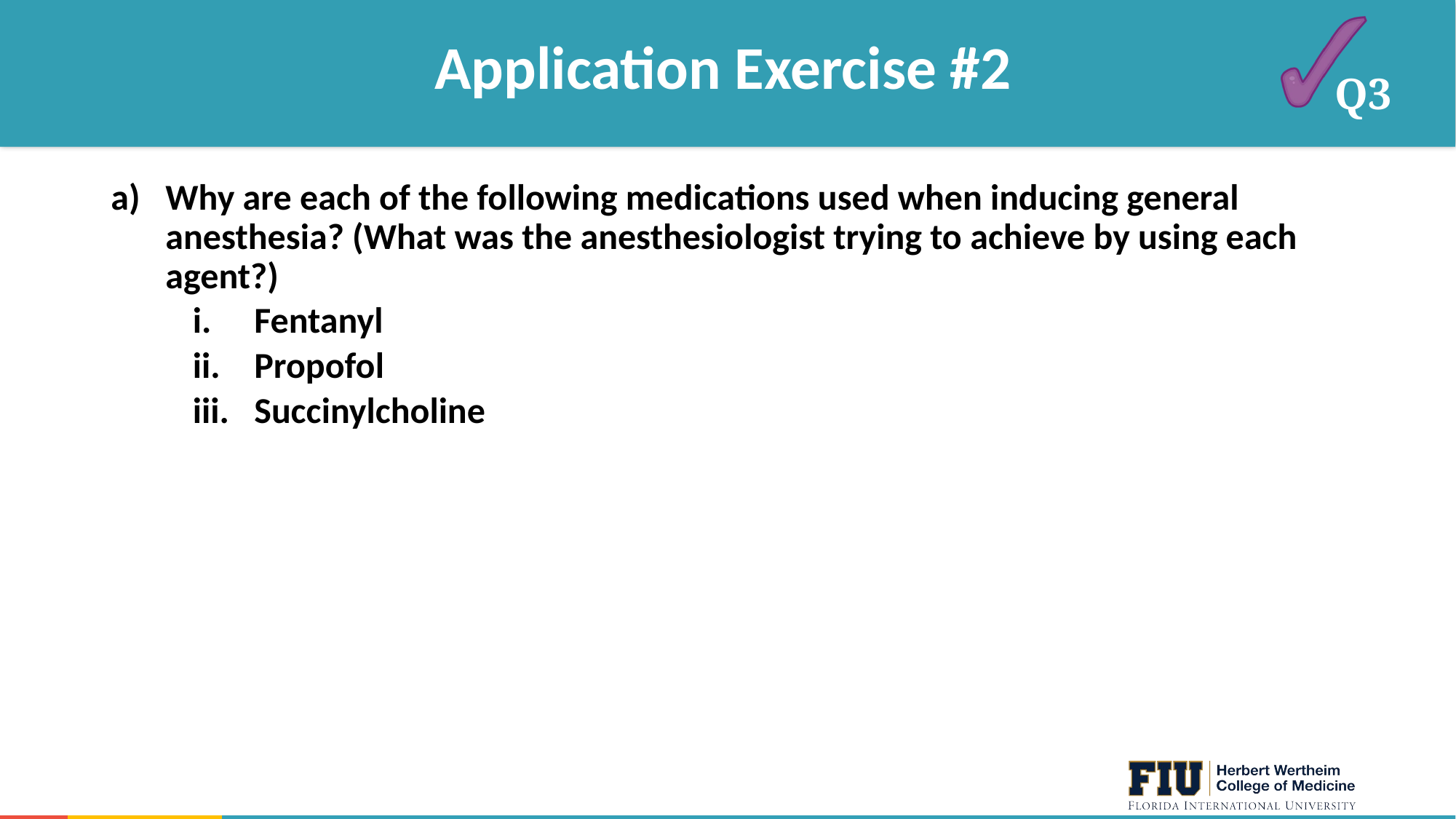

# Application Exercise #2
Q3
Why are each of the following medications used when inducing general anesthesia? (What was the anesthesiologist trying to achieve by using each agent?)
Fentanyl
Propofol
Succinylcholine

## Slide 16
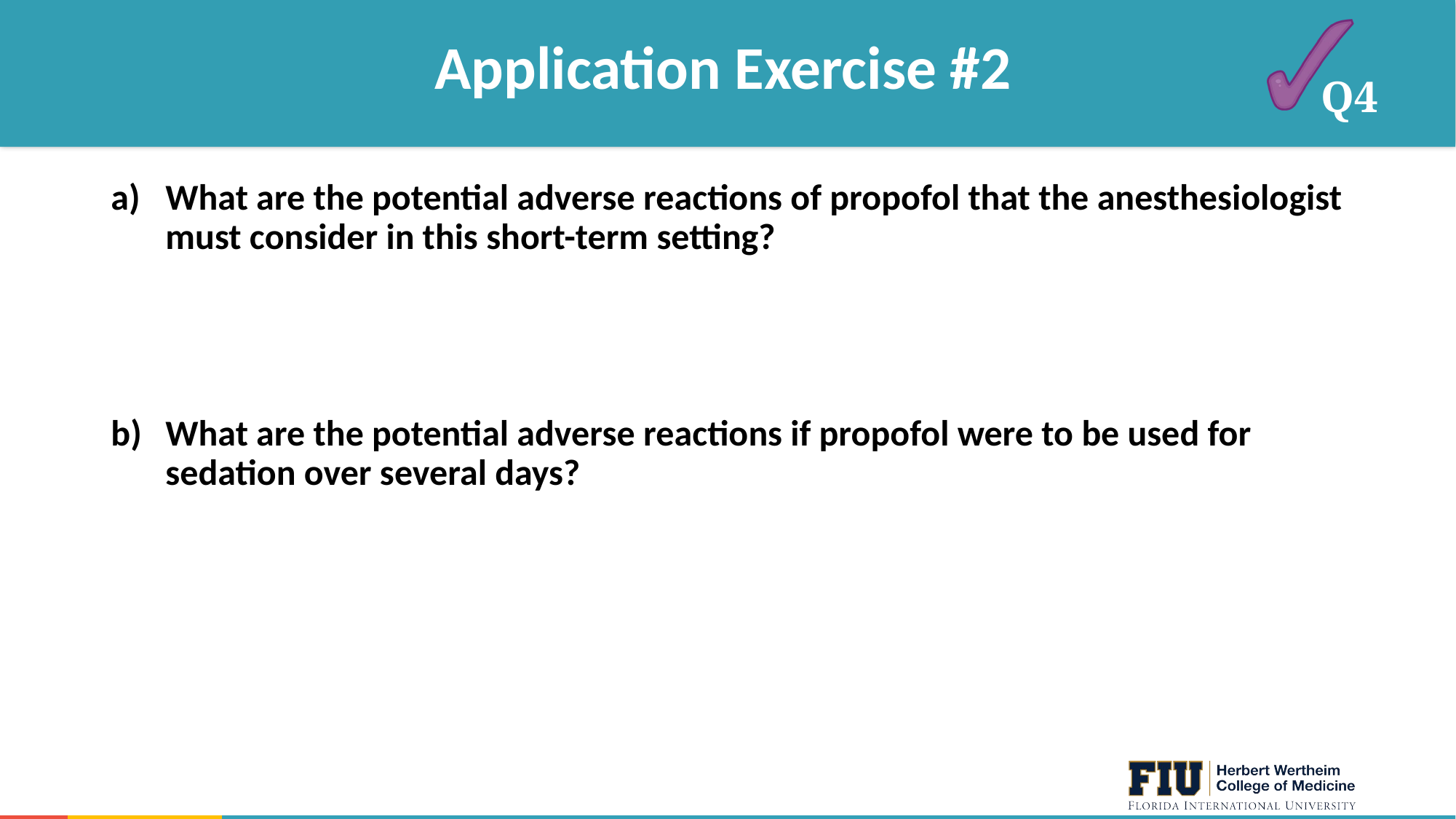

# Application Exercise #2
Q4
What are the potential adverse reactions of propofol that the anesthesiologist must consider in this short-term setting?
What are the potential adverse reactions if propofol were to be used for sedation over several days?

## Slide 17
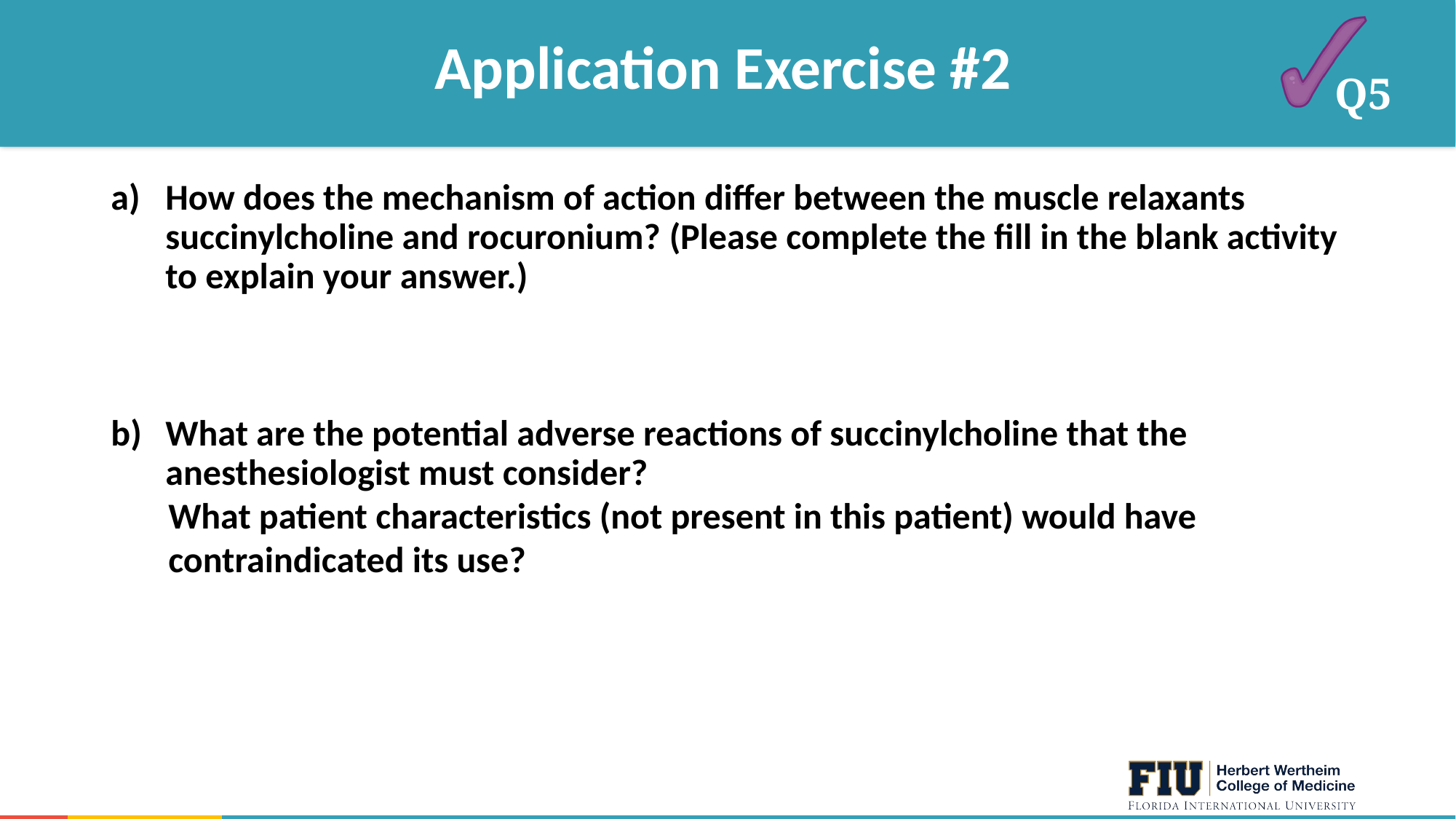

# Application Exercise #2
Q5
How does the mechanism of action differ between the muscle relaxants succinylcholine and rocuronium? (Please complete the fill in the blank activity to explain your answer.)
What are the potential adverse reactions of succinylcholine that the anesthesiologist must consider?
 What patient characteristics (not present in this patient) would have
 contraindicated its use?

## Slide 18
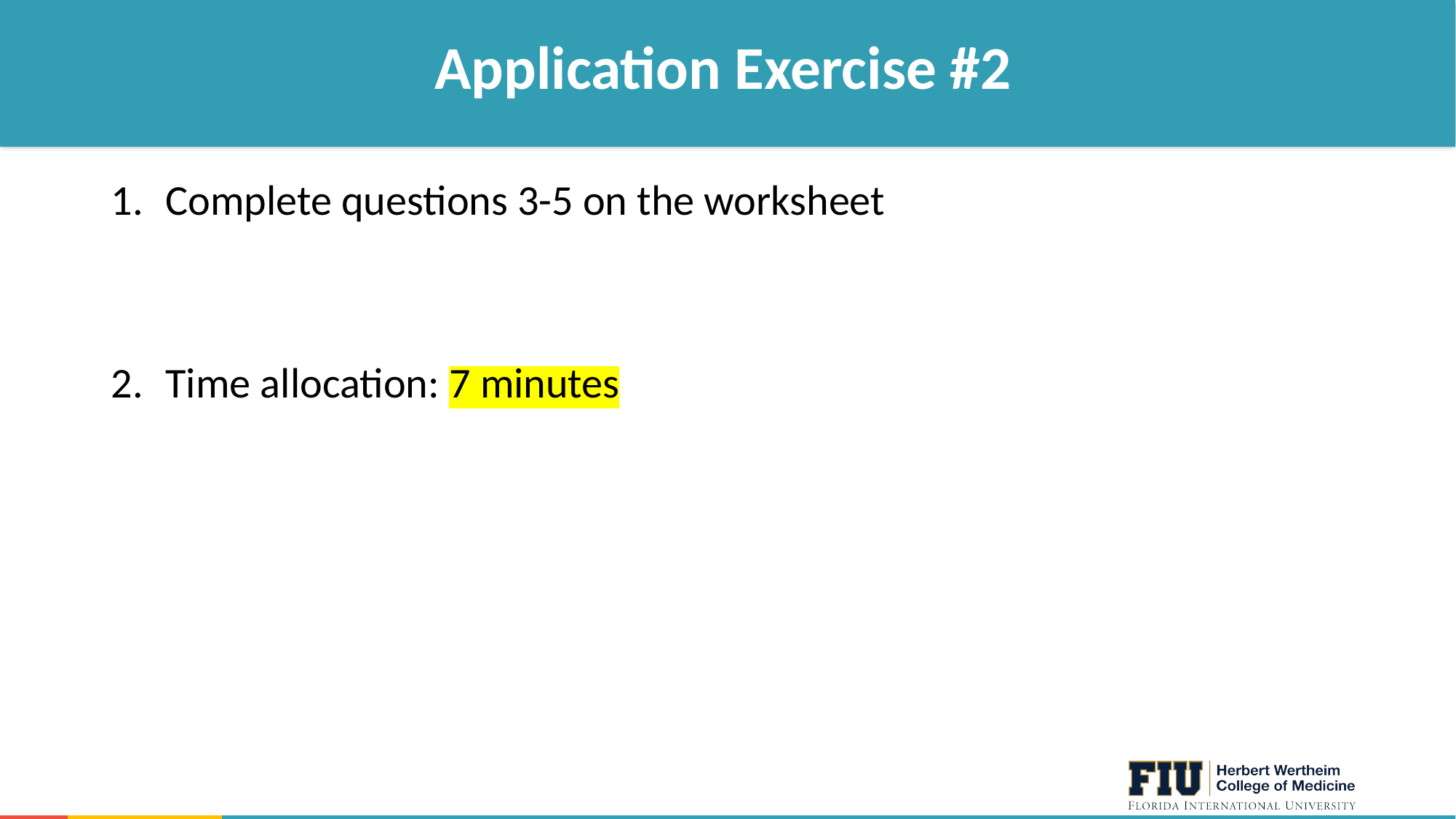

# Application Exercise #2
Complete questions 3-5 on the worksheet
Time allocation: 7 minutes

## Slide 19
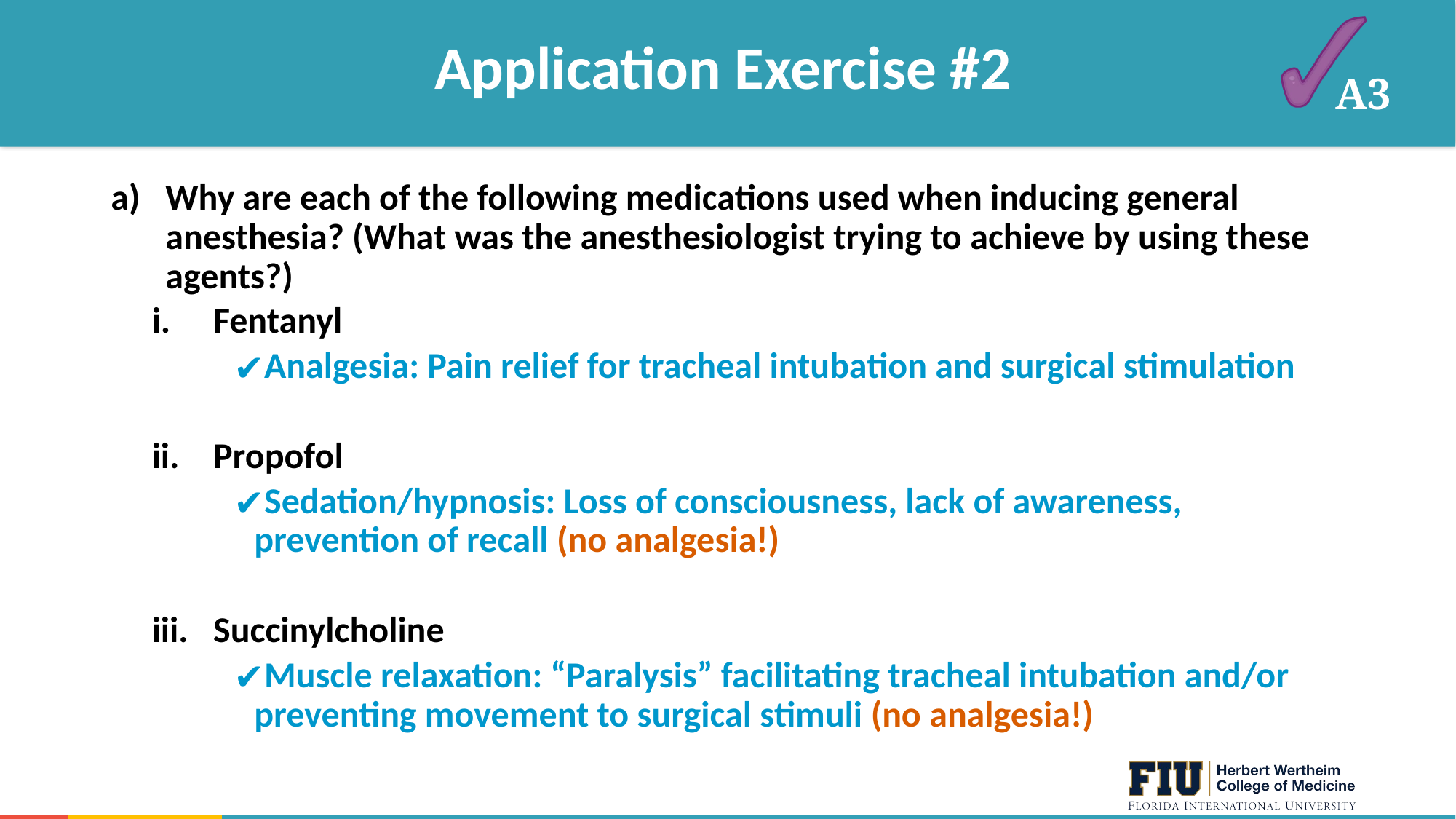

# Application Exercise #2
A3
Why are each of the following medications used when inducing general anesthesia? (What was the anesthesiologist trying to achieve by using these agents?)
Fentanyl
Analgesia: Pain relief for tracheal intubation and surgical stimulation
Propofol
Sedation/hypnosis: Loss of consciousness, lack of awareness, prevention of recall (no analgesia!)
Succinylcholine
Muscle relaxation: “Paralysis” facilitating tracheal intubation and/or preventing movement to surgical stimuli (no analgesia!)

## Slide 20
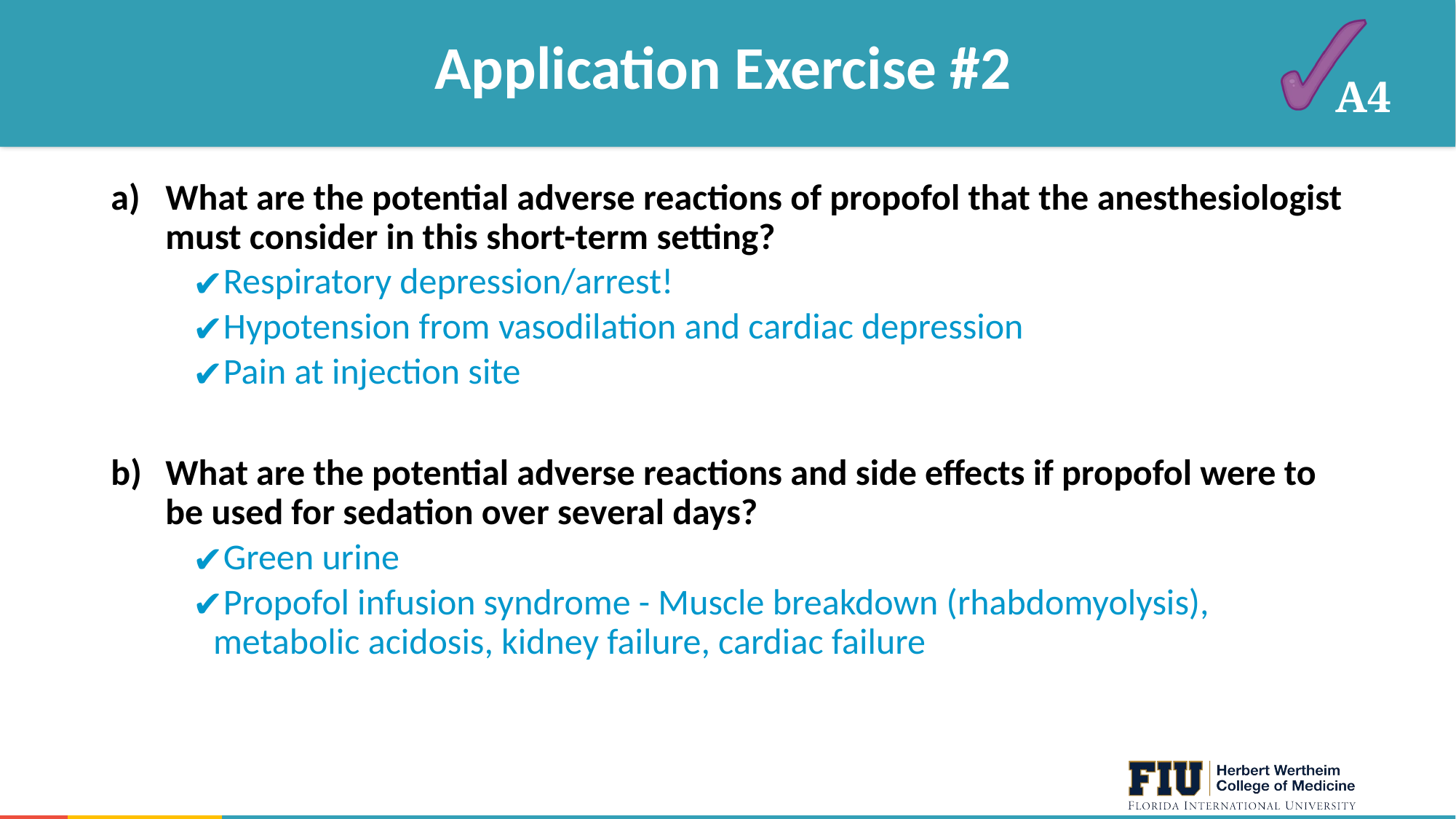

# Application Exercise #2
A4
What are the potential adverse reactions of propofol that the anesthesiologist must consider in this short-term setting?
Respiratory depression/arrest!
Hypotension from vasodilation and cardiac depression
Pain at injection site
What are the potential adverse reactions and side effects if propofol were to be used for sedation over several days?
Green urine
Propofol infusion syndrome - Muscle breakdown (rhabdomyolysis), metabolic acidosis, kidney failure, cardiac failure

## Slide 21
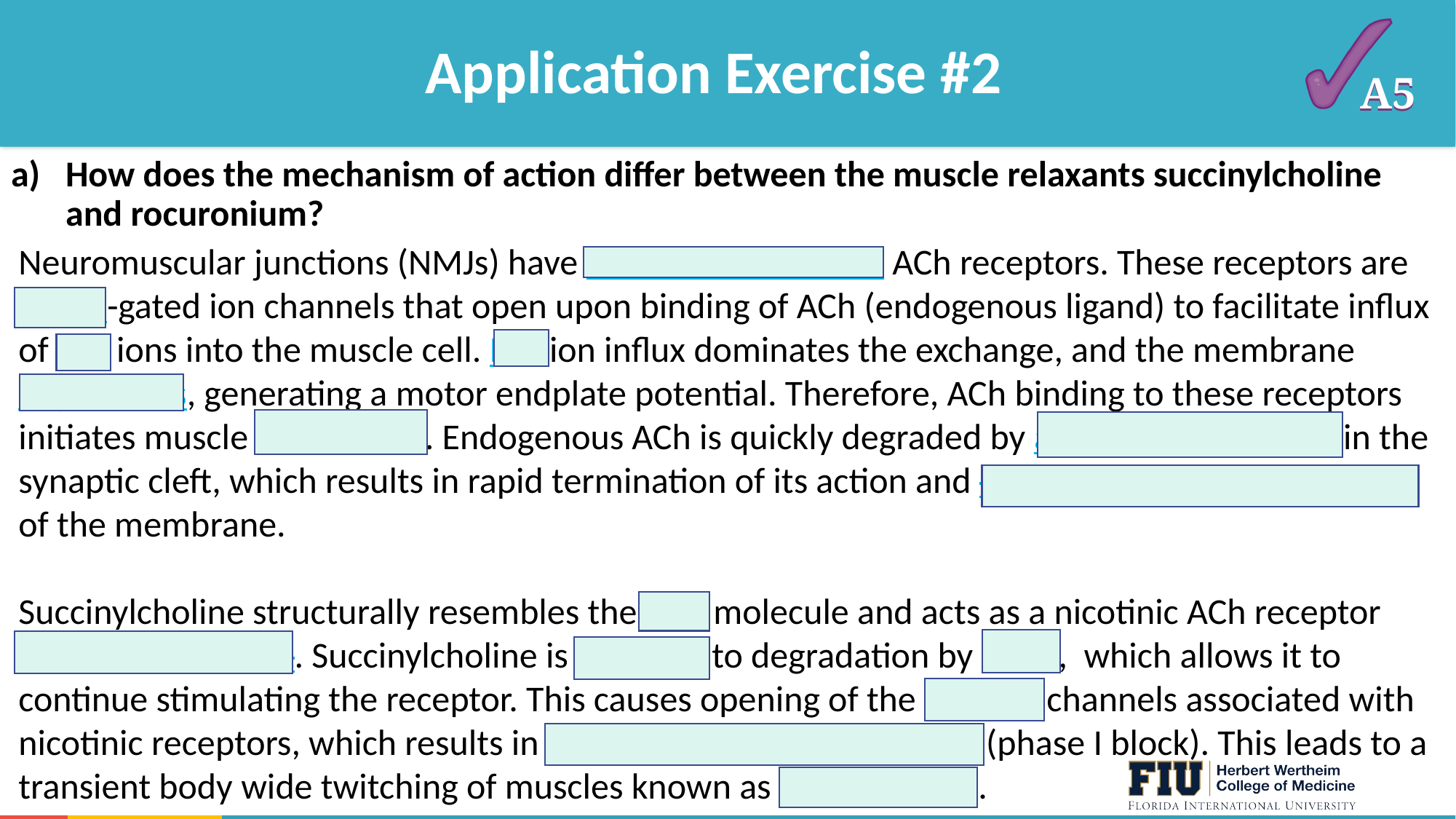

A5
Application Exercise #2
A5
How does the mechanism of action differ between the muscle relaxants succinylcholine and rocuronium?
Neuromuscular junctions (NMJs) have nicotinic/muscarinic ACh receptors. These receptors are ligand-gated ion channels that open upon binding of ACh (endogenous ligand) to facilitate influx of Na+ ions into the muscle cell. Na+ ion influx dominates the exchange, and the membrane depolarizes, generating a motor endplate potential. Therefore, ACh binding to these receptors initiates muscle contraction. Endogenous ACh is quickly degraded by acetylcholinesterase in the synaptic cleft, which results in rapid termination of its action and depolarization/repolarization of the membrane.
Succinylcholine structurally resembles the ACh molecule and acts as a nicotinic ACh receptor agonist/antagonist. Succinylcholine is resistant to degradation by AChE, which allows it to continue stimulating the receptor. This causes opening of the Na+ ion channels associated with nicotinic receptors, which results in depolarization/repolarization (phase I block). This leads to a transient body wide twitching of muscles known as fasciculations.

## Slide 22
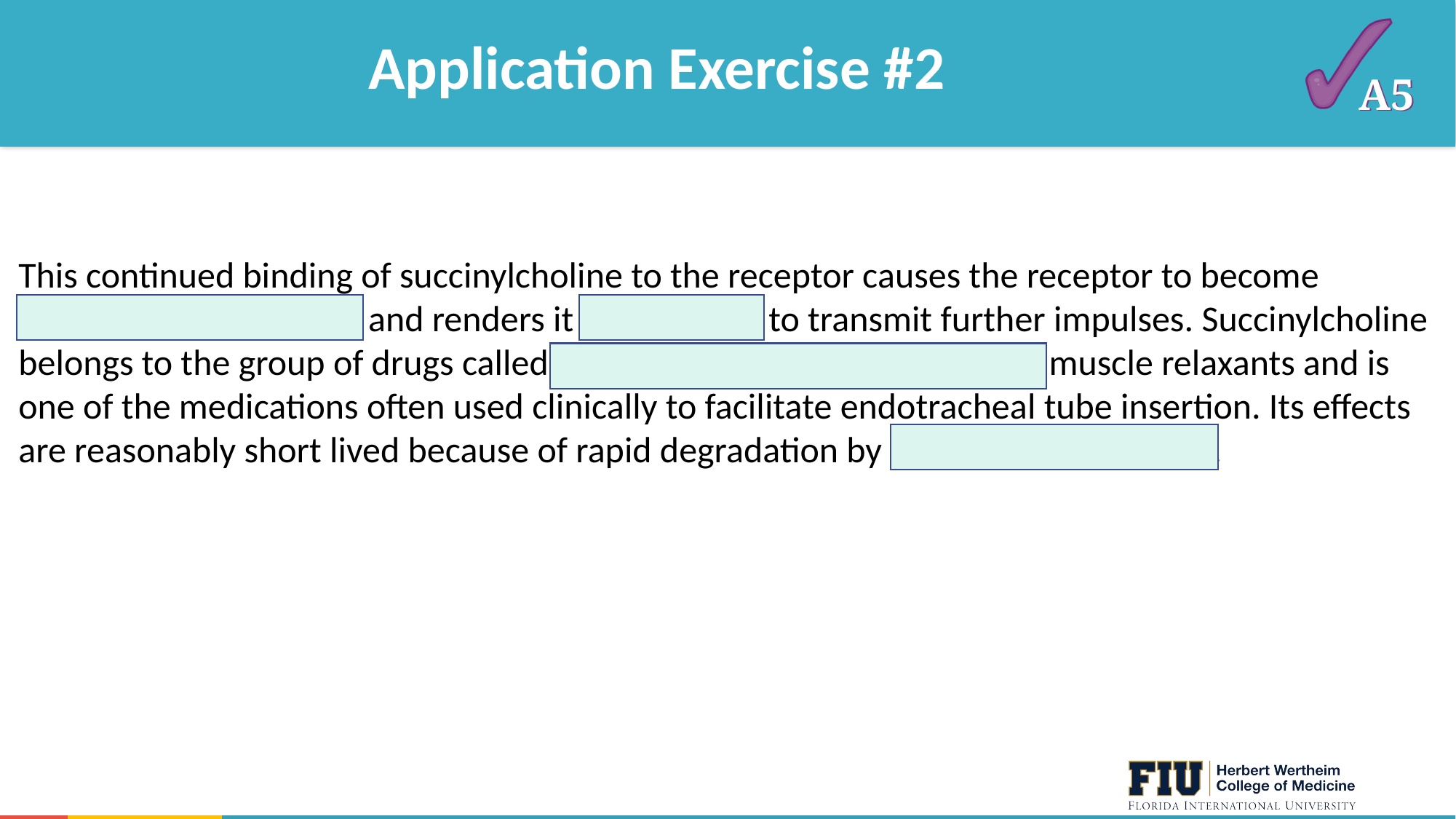

Application Exercise #2
A5
#
A5
This continued binding of succinylcholine to the receptor causes the receptor to become sensitized/desensitized and renders it unable/able to transmit further impulses. Succinylcholine belongs to the group of drugs called depolarizing/non–depolarization muscle relaxants and is one of the medications often used clinically to facilitate endotracheal tube insertion. Its effects are reasonably short lived because of rapid degradation by pseudocholinesterase.

## Slide 23
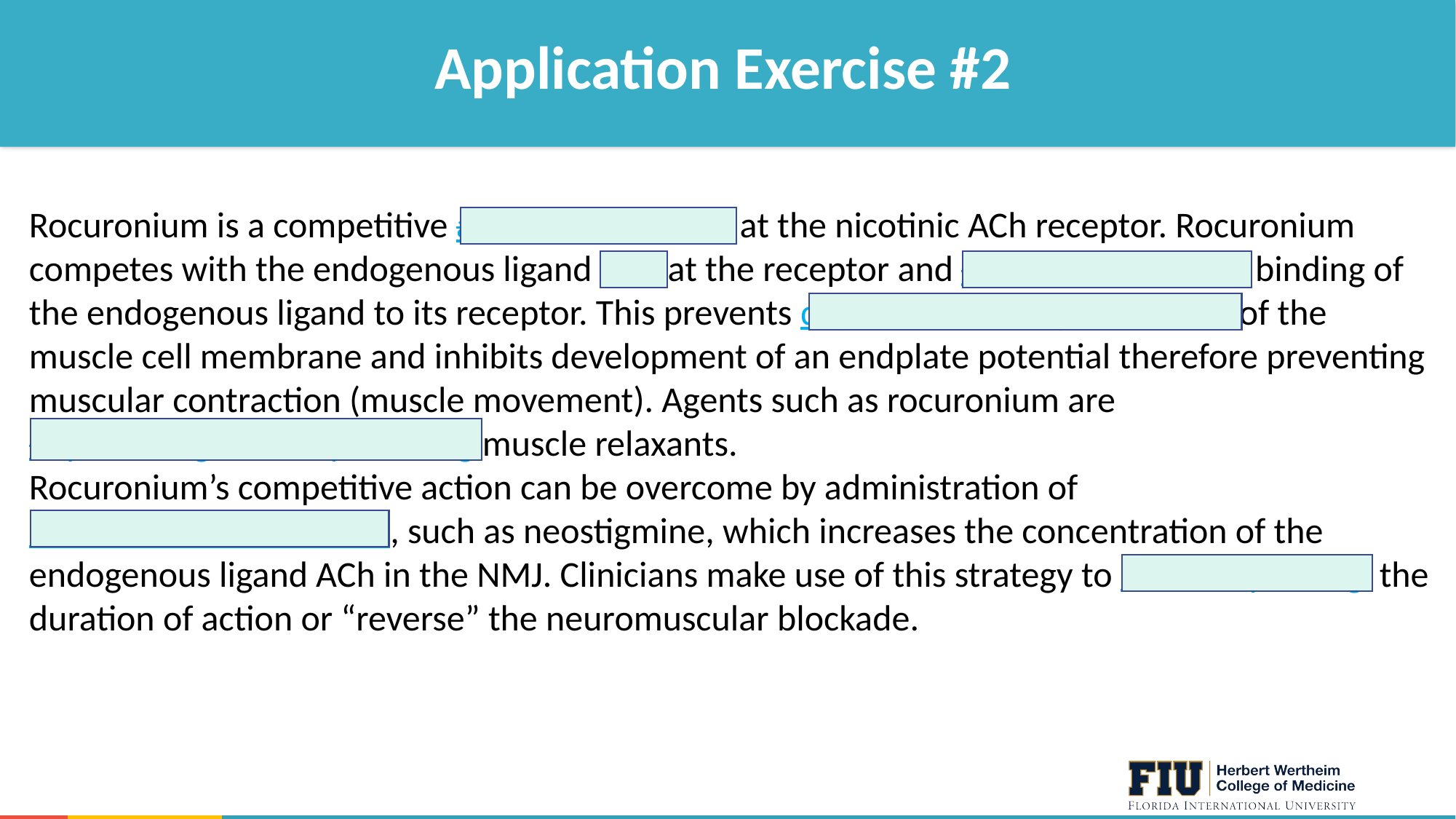

# Application Exercise #2
A5
A5
Rocuronium is a competitive agonist/antagonist at the nicotinic ACh receptor. Rocuronium competes with the endogenous ligand ACh at the receptor and promotes/prevents binding of the endogenous ligand to its receptor. This prevents depolarization/repolarization of the muscle cell membrane and inhibits development of an endplate potential therefore preventing muscular contraction (muscle movement). Agents such as rocuronium are
depolarizing/non-depolarizing muscle relaxants.
Rocuronium’s competitive action can be overcome by administration of
cholinesterase inhibitors, such as neostigmine, which increases the concentration of the endogenous ligand ACh in the NMJ. Clinicians make use of this strategy to shorten/prolong the duration of action or “reverse” the neuromuscular blockade.

## Slide 24
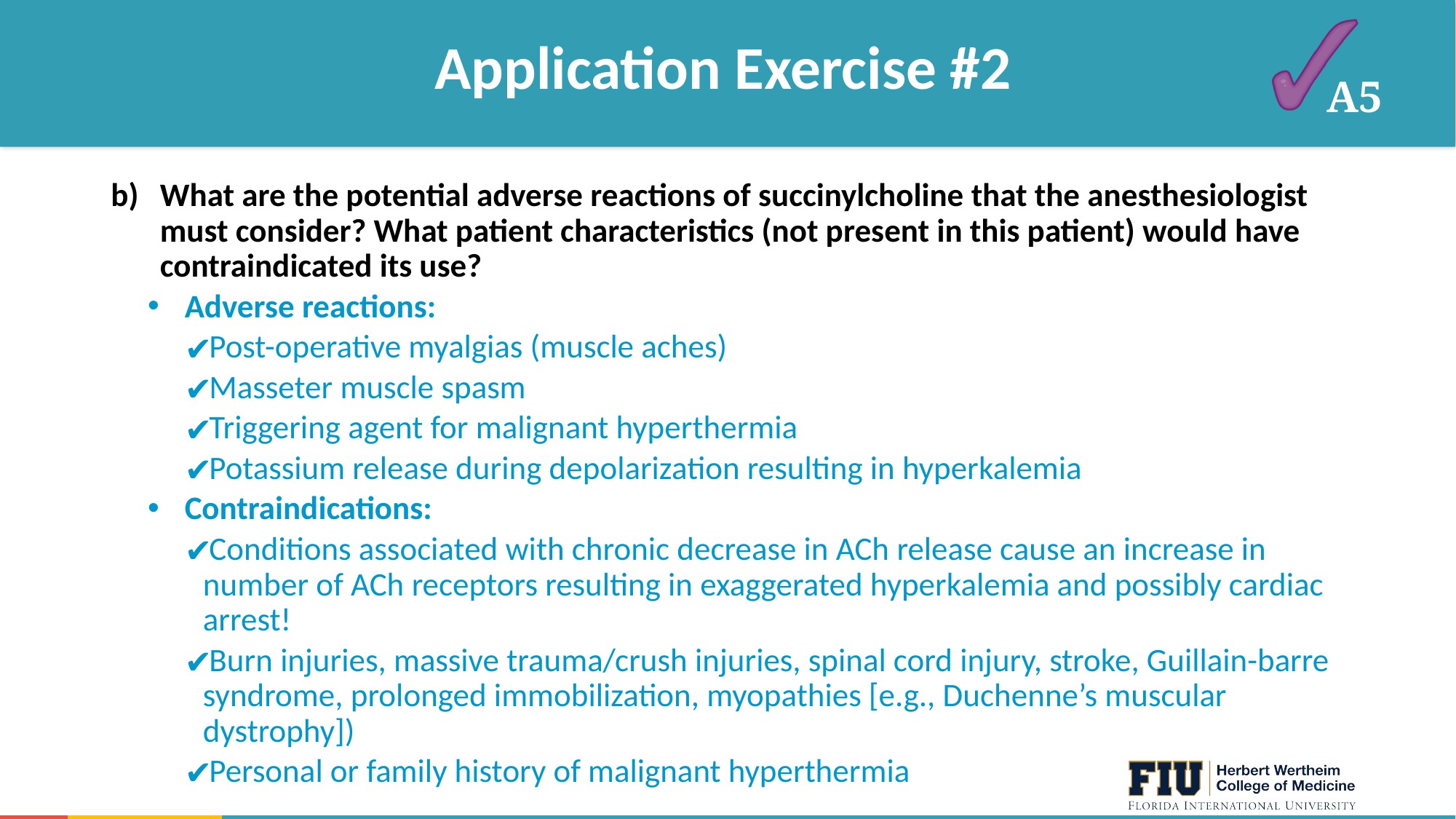

# Application Exercise #2
A5
What are the potential adverse reactions of succinylcholine that the anesthesiologist must consider? What patient characteristics (not present in this patient) would have contraindicated its use?
Adverse reactions:
Post-operative myalgias (muscle aches)
Masseter muscle spasm
Triggering agent for malignant hyperthermia
Potassium release during depolarization resulting in hyperkalemia
Contraindications:
Conditions associated with chronic decrease in ACh release cause an increase in number of ACh receptors resulting in exaggerated hyperkalemia and possibly cardiac arrest!
Burn injuries, massive trauma/crush injuries, spinal cord injury, stroke, Guillain-barre syndrome, prolonged immobilization, myopathies [e.g., Duchenne’s muscular dystrophy])
Personal or family history of malignant hyperthermia

## Slide 25
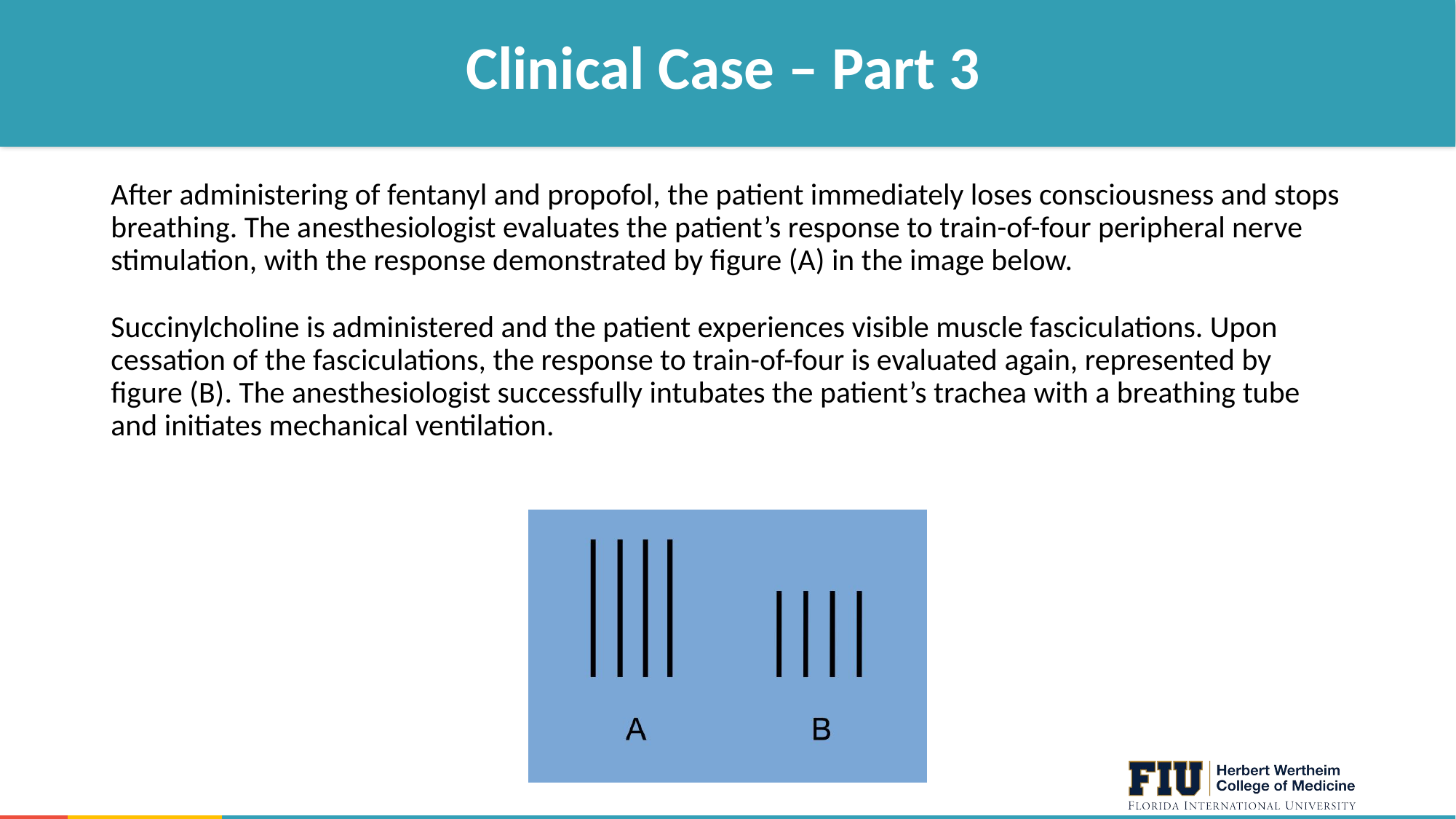

# Clinical Case – Part 3
After administering of fentanyl and propofol, the patient immediately loses consciousness and stops breathing. The anesthesiologist evaluates the patient’s response to train-of-four peripheral nerve stimulation, with the response demonstrated by figure (A) in the image below.
Succinylcholine is administered and the patient experiences visible muscle fasciculations. Upon cessation of the fasciculations, the response to train-of-four is evaluated again, represented by figure (B). The anesthesiologist successfully intubates the patient’s trachea with a breathing tube and initiates mechanical ventilation.

## Slide 26
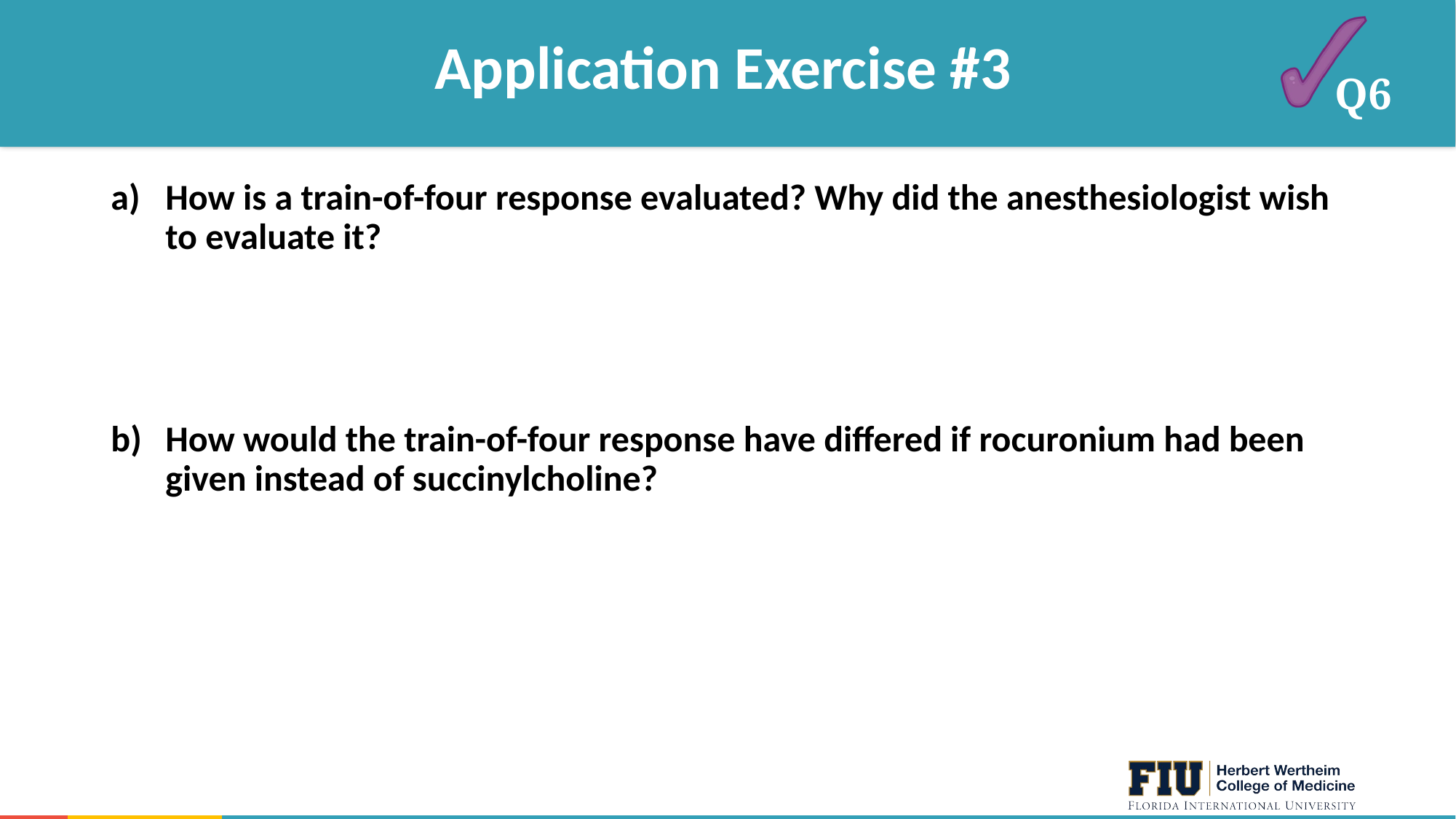

# Application Exercise #3
Q6
How is a train-of-four response evaluated? Why did the anesthesiologist wish to evaluate it?
How would the train-of-four response have differed if rocuronium had been given instead of succinylcholine?

## Slide 27
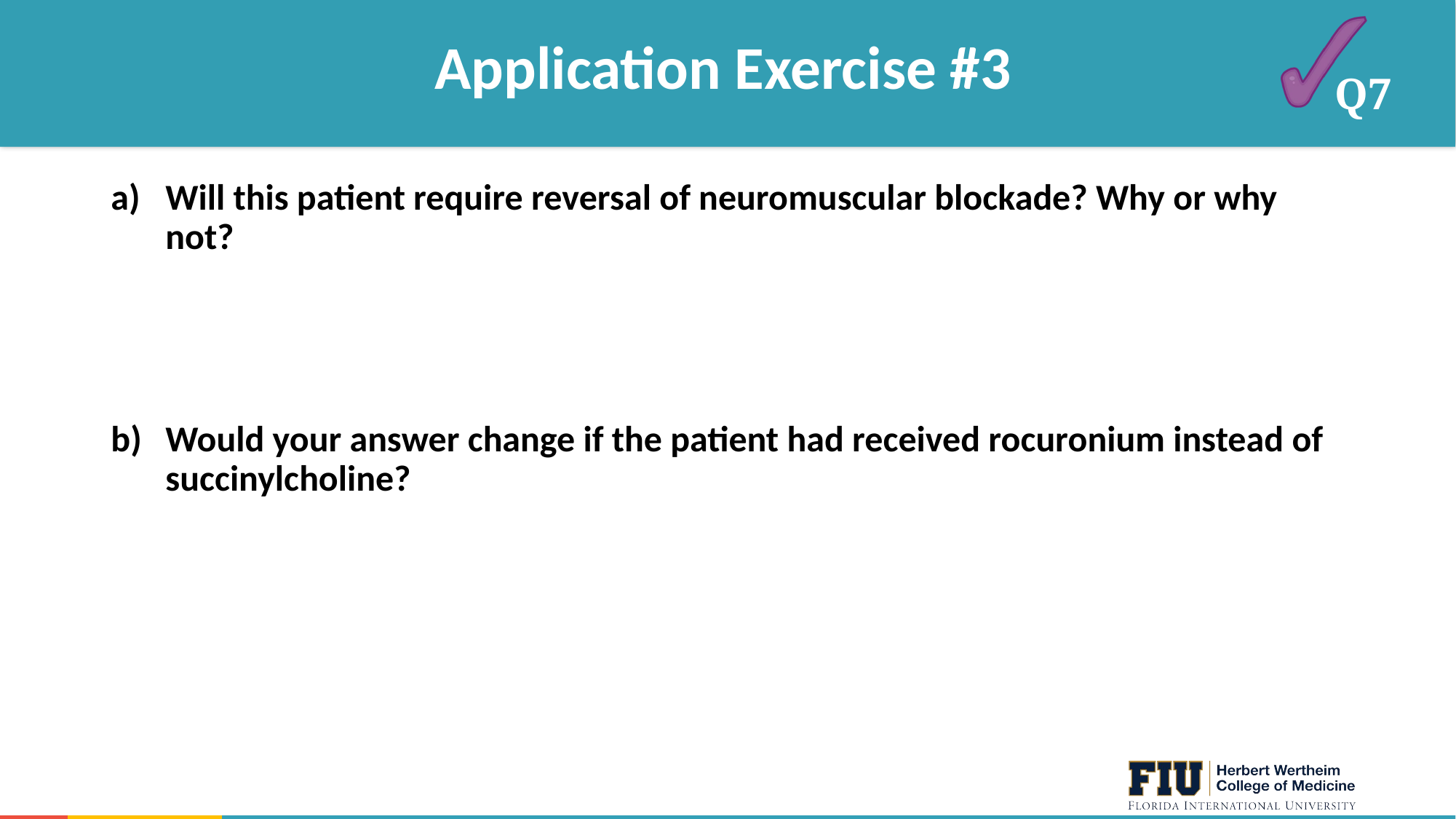

# Application Exercise #3
Q7
Will this patient require reversal of neuromuscular blockade? Why or why not?
Would your answer change if the patient had received rocuronium instead of succinylcholine?

## Slide 28
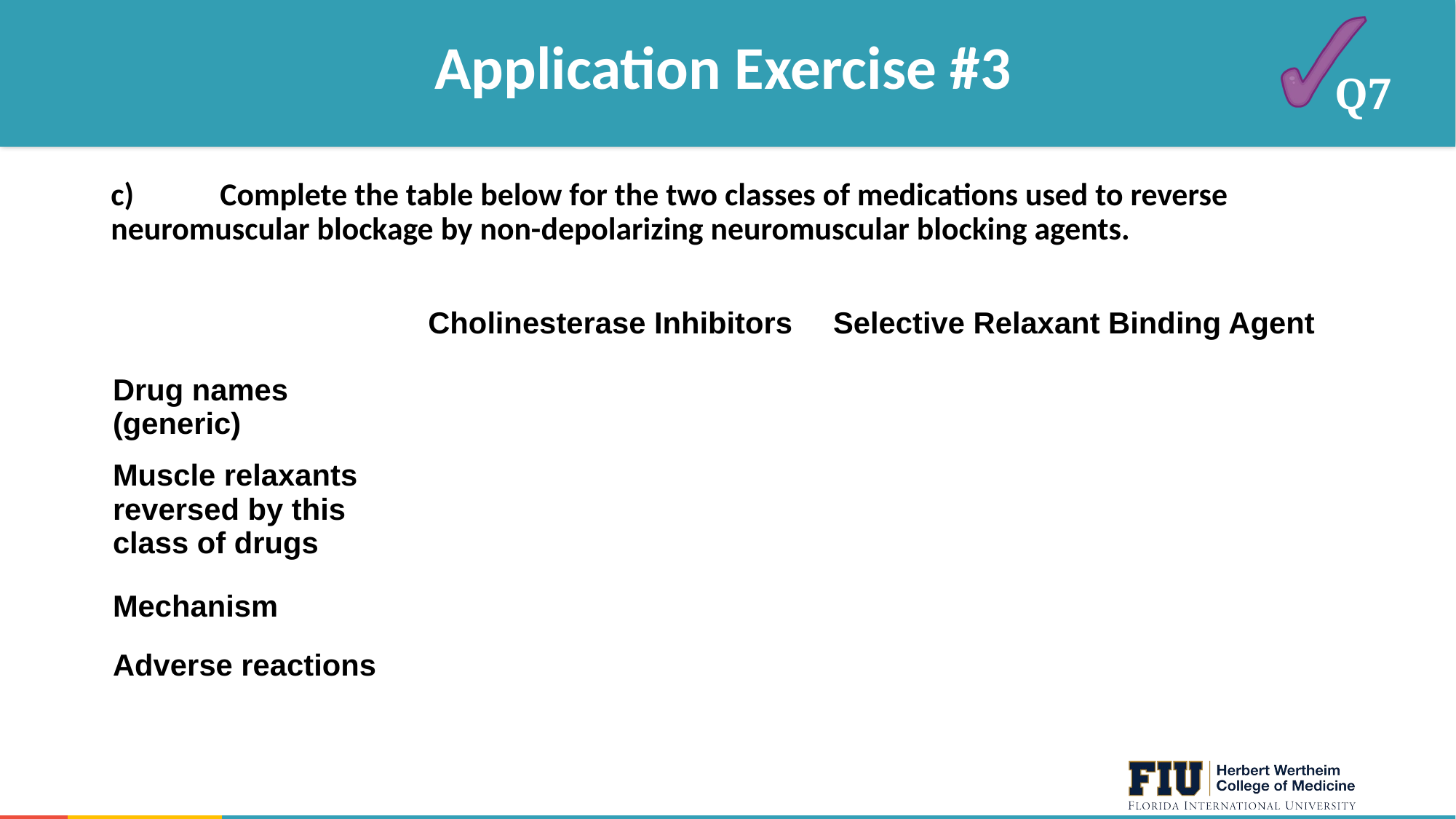

# Application Exercise #3
Q7
c)	Complete the table below for the two classes of medications used to reverse 	neuromuscular blockage by non-depolarizing neuromuscular blocking agents.
| | Cholinesterase Inhibitors | Selective Relaxant Binding Agent |
| --- | --- | --- |
| Drug names (generic) | | |
| Muscle relaxants reversed by this class of drugs | | |
| Mechanism | | |
| Adverse reactions | | |

## Slide 29
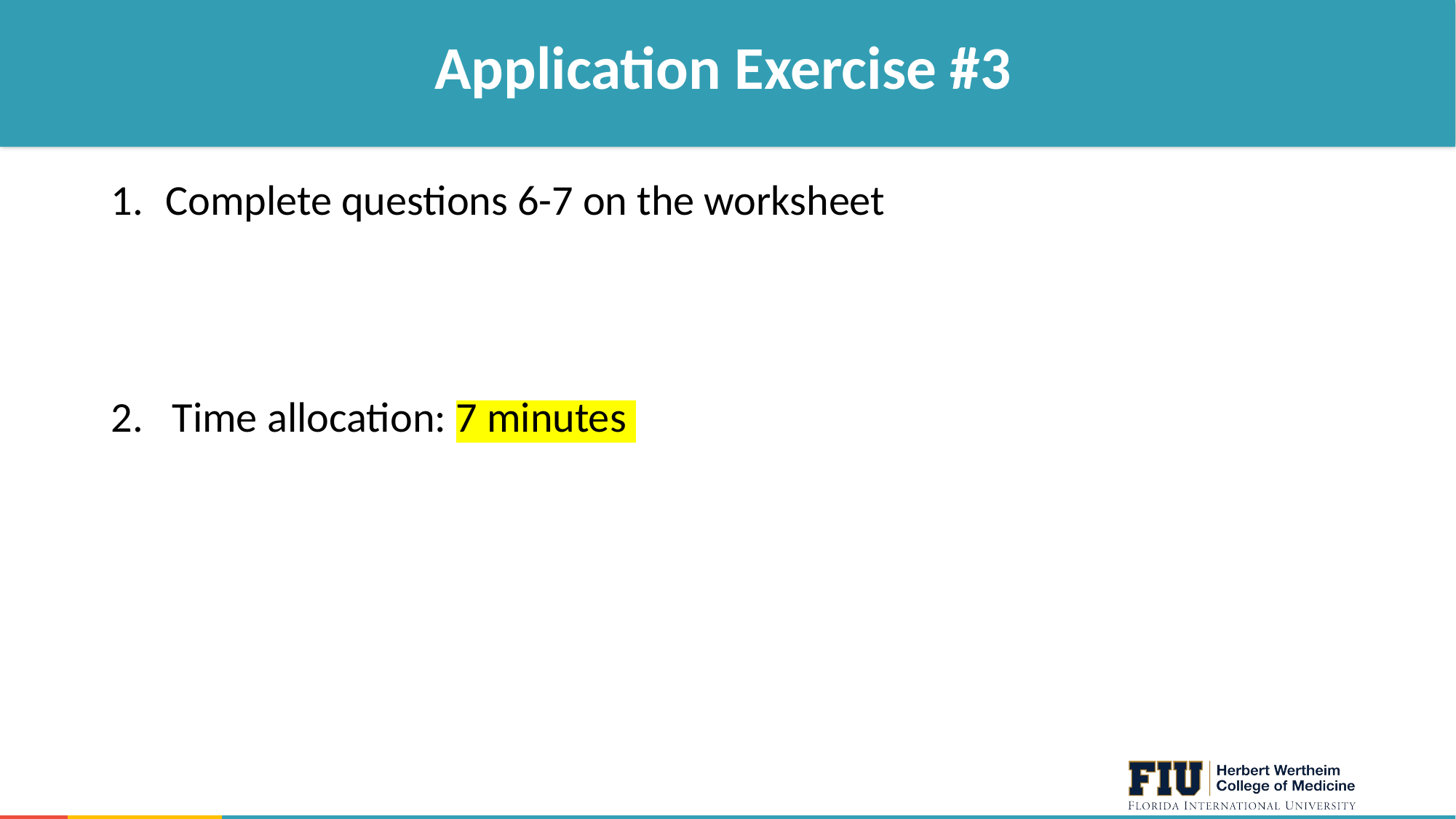

# Application Exercise #3
Complete questions 6-7 on the worksheet
Time allocation: 7 minutes

## Slide 30
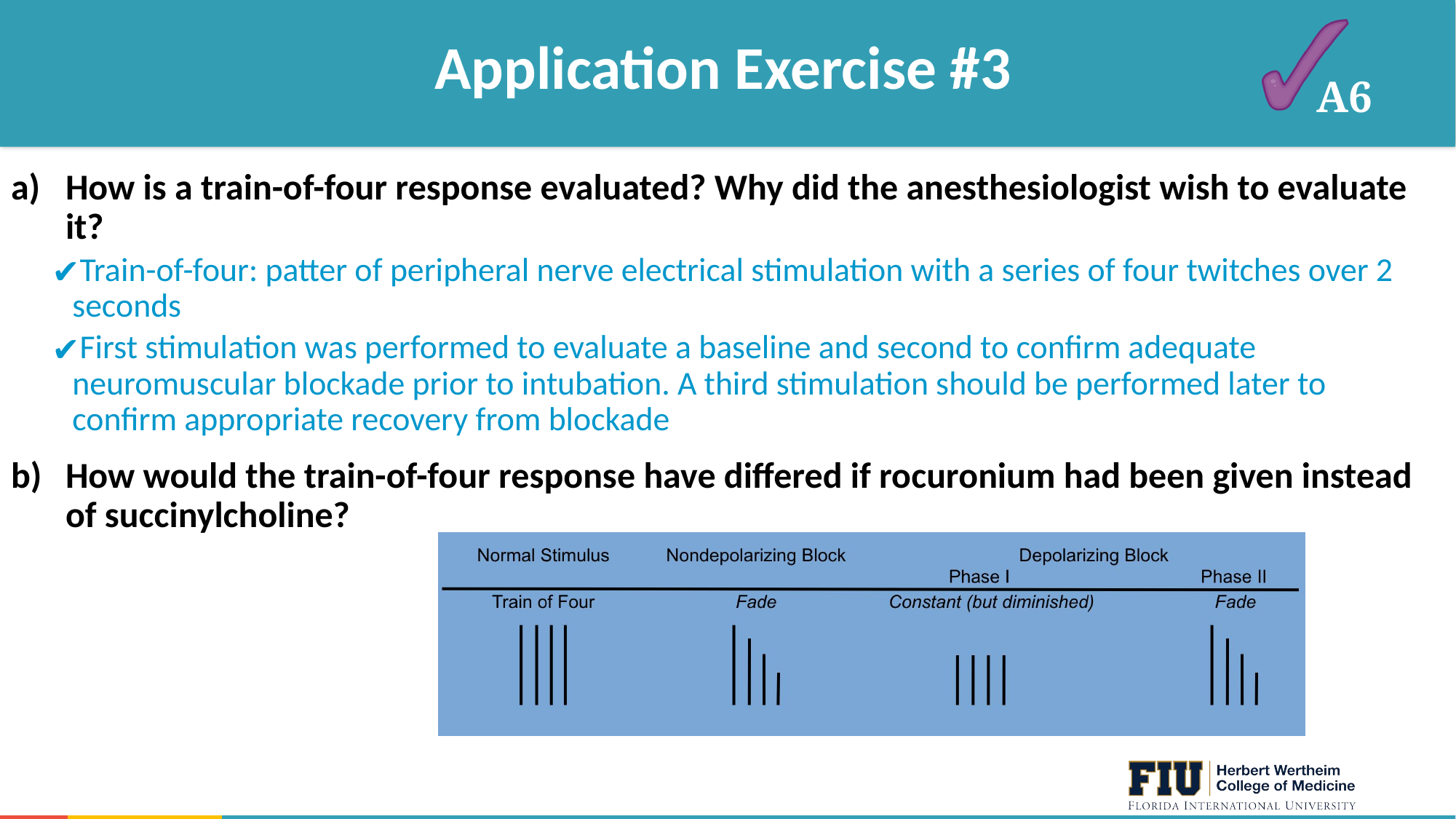

# Application Exercise #3
A6
How is a train-of-four response evaluated? Why did the anesthesiologist wish to evaluate it?
Train-of-four: patter of peripheral nerve electrical stimulation with a series of four twitches over 2 seconds
First stimulation was performed to evaluate a baseline and second to confirm adequate neuromuscular blockade prior to intubation. A third stimulation should be performed later to confirm appropriate recovery from blockade
How would the train-of-four response have differed if rocuronium had been given instead of succinylcholine?

## Slide 31
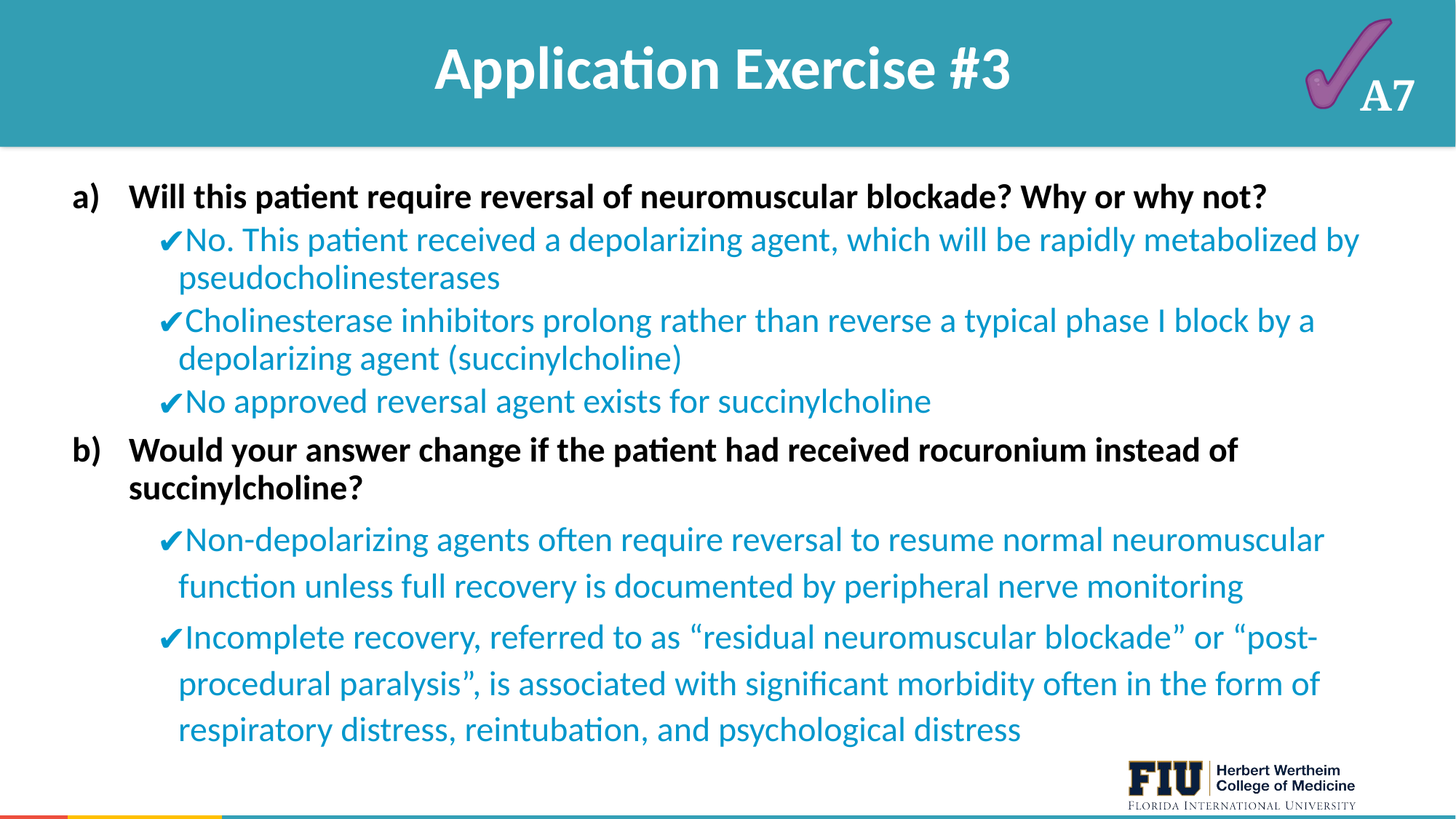

# Application Exercise #3
A7
Will this patient require reversal of neuromuscular blockade? Why or why not?
No. This patient received a depolarizing agent, which will be rapidly metabolized by pseudocholinesterases
Cholinesterase inhibitors prolong rather than reverse a typical phase I block by a depolarizing agent (succinylcholine)
No approved reversal agent exists for succinylcholine
Would your answer change if the patient had received rocuronium instead of succinylcholine?
Non-depolarizing agents often require reversal to resume normal neuromuscular function unless full recovery is documented by peripheral nerve monitoring
Incomplete recovery, referred to as “residual neuromuscular blockade” or “post-procedural paralysis”, is associated with significant morbidity often in the form of respiratory distress, reintubation, and psychological distress

## Slide 32
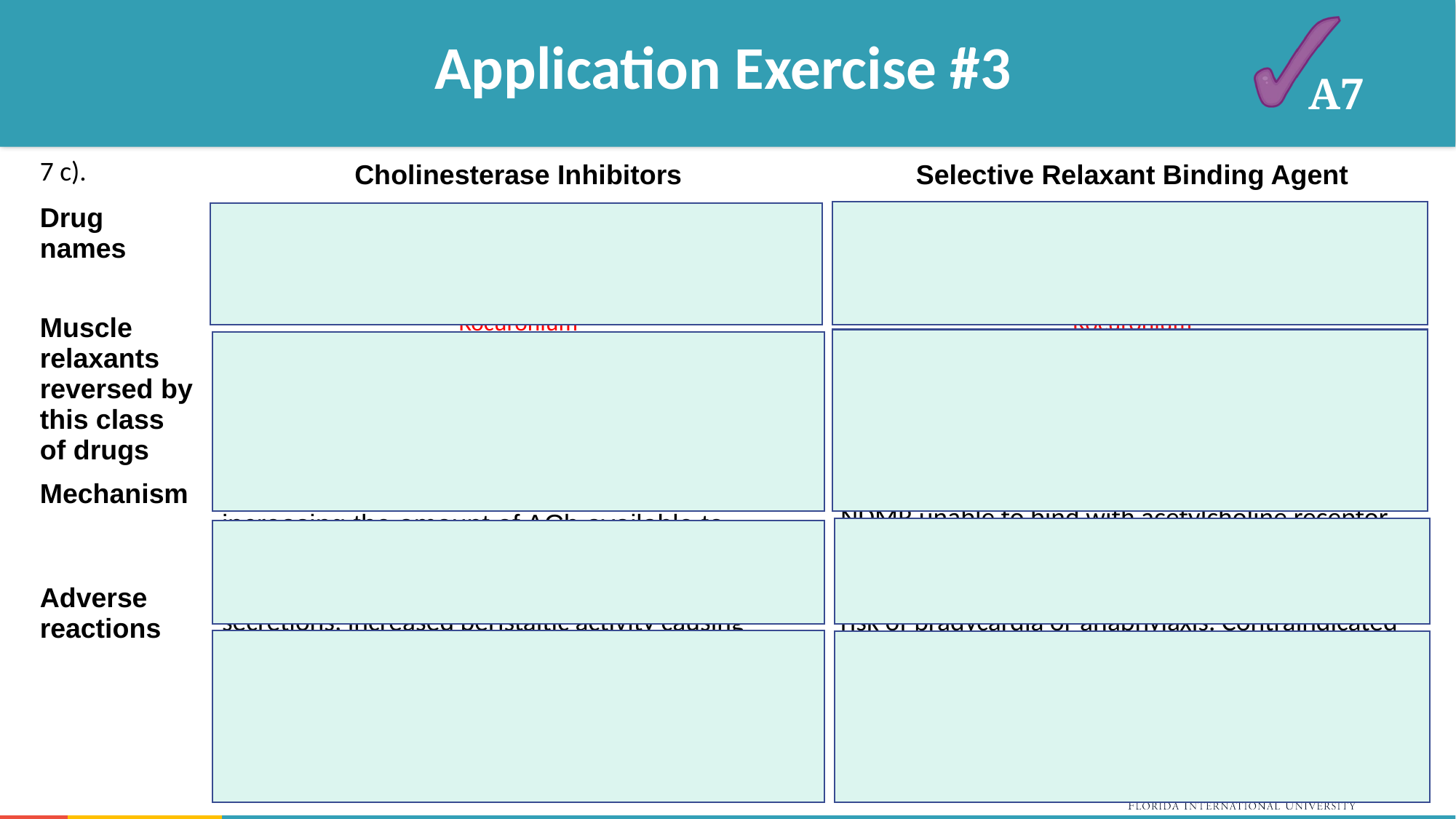

# Application Exercise #3
A7
| 7 c). | Cholinesterase Inhibitors | Selective Relaxant Binding Agent |
| --- | --- | --- |
| Drug names | Neostigmine Pyridostigmine Physostigmine Endrophonium | Sugammadex |
| Muscle relaxants reversed by this class of drugs | Rocuronium Vecuronium Pancuronium Mivacurium Atracurium Cisatracurium | Rocuronium Vecuronium |
| Mechanism | Inactivates acetylcholinesterase indirectly increasing the amount of ACh available to compete with NDMR | Binds NDMR forming a complex rendering the NDMR unable to bind with acetylcholine receptor |
| Adverse reactions | Profound bradycardia, bronchospasm, increased secretions, increased peristaltic activity causing postoperative nauseas, vomiting, and fecal incontinence/diarrhea (Think of acetylcholine stimulation on muscarinic receptors!) | Decreased efficacy of oral contraceptives, very small risk of bradycardia or anaphylaxis. Contraindicated in renal failure (renally excreted drug) |

## Slide 33
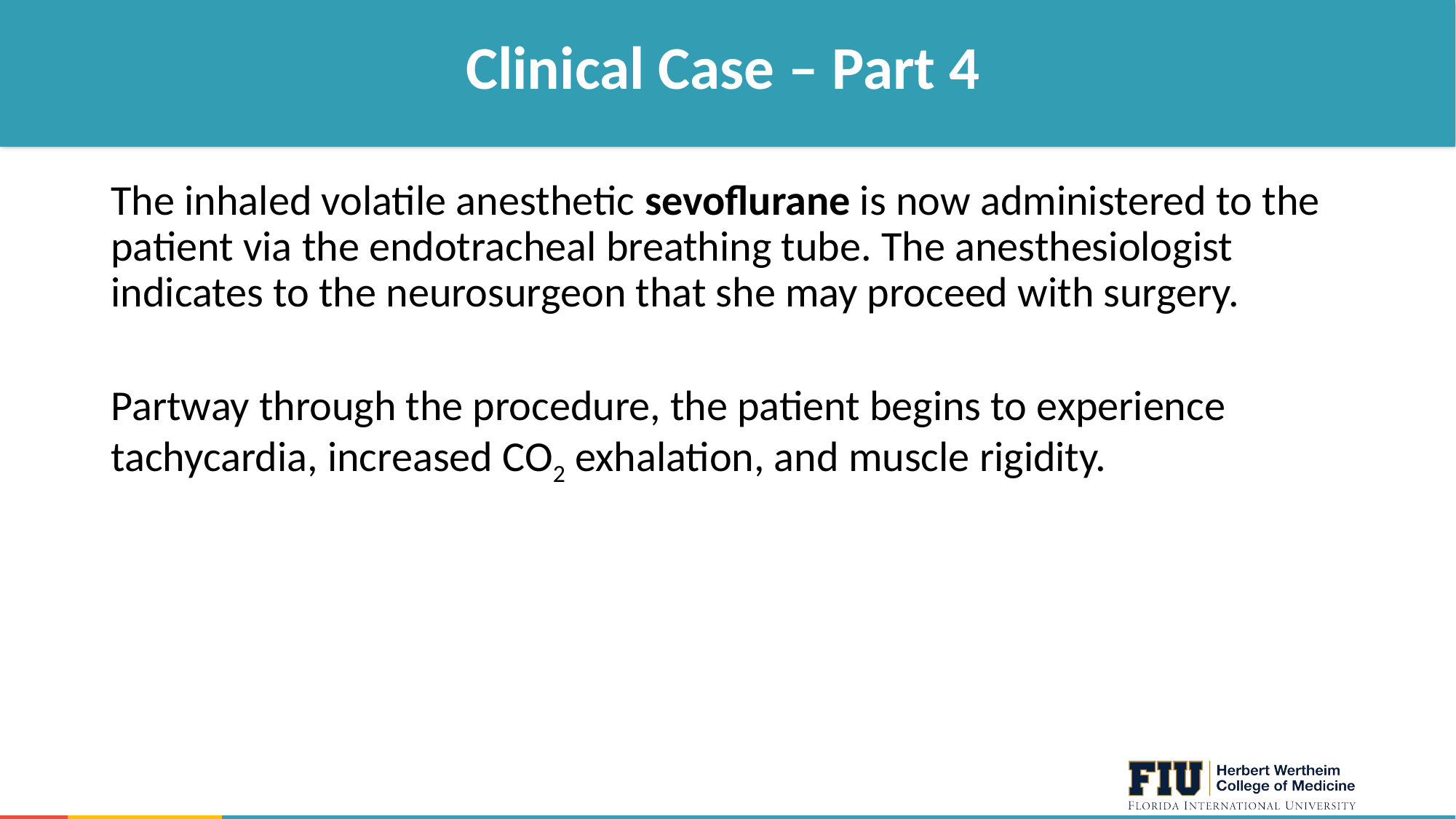

# Clinical Case – Part 4
The inhaled volatile anesthetic sevoflurane is now administered to the patient via the endotracheal breathing tube. The anesthesiologist indicates to the neurosurgeon that she may proceed with surgery.
Partway through the procedure, the patient begins to experience tachycardia, increased CO2 exhalation, and muscle rigidity.

## Slide 34
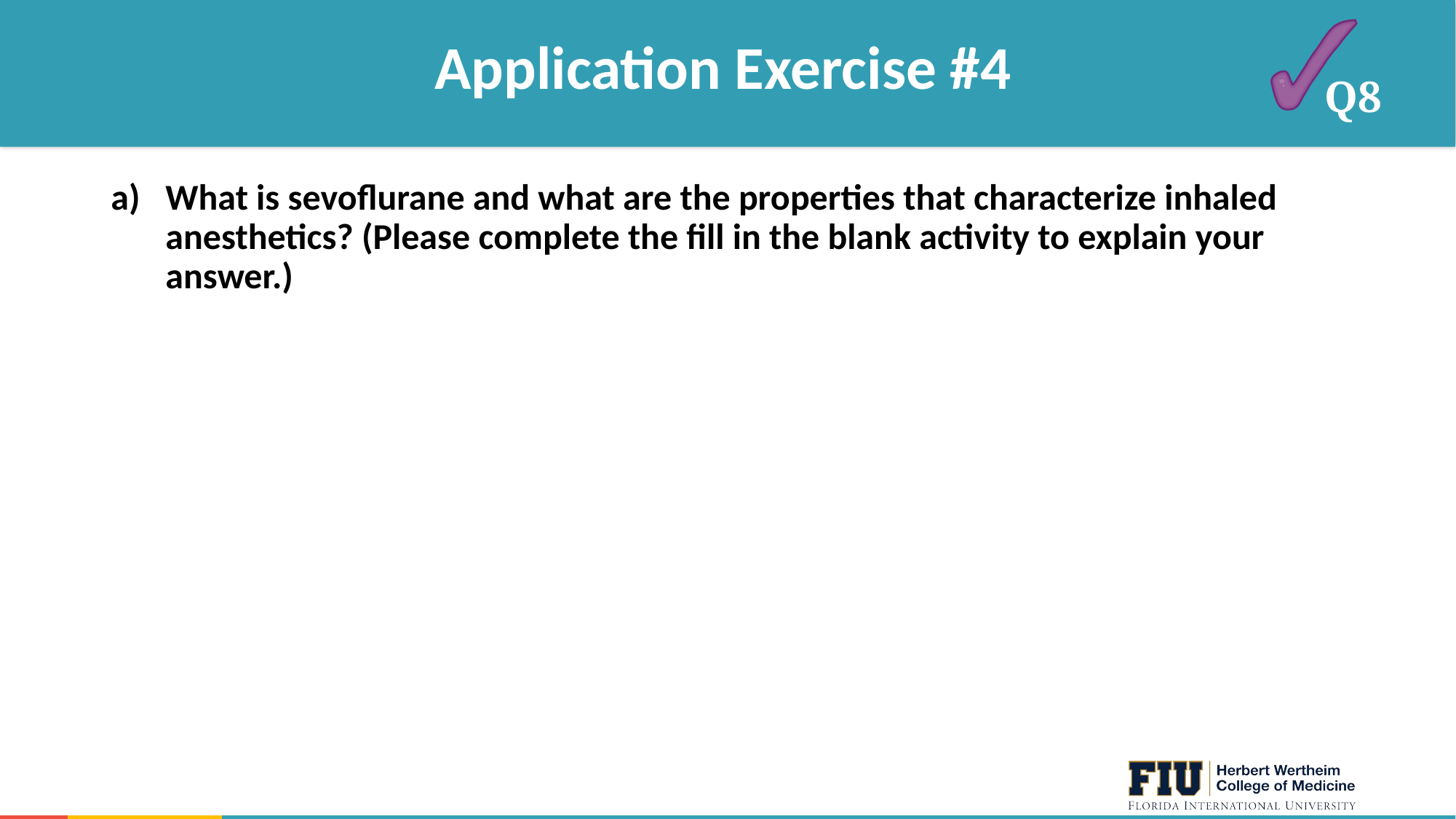

# Application Exercise #4
Q8
What is sevoflurane and what are the properties that characterize inhaled anesthetics? (Please complete the fill in the blank activity to explain your answer.)

## Slide 35
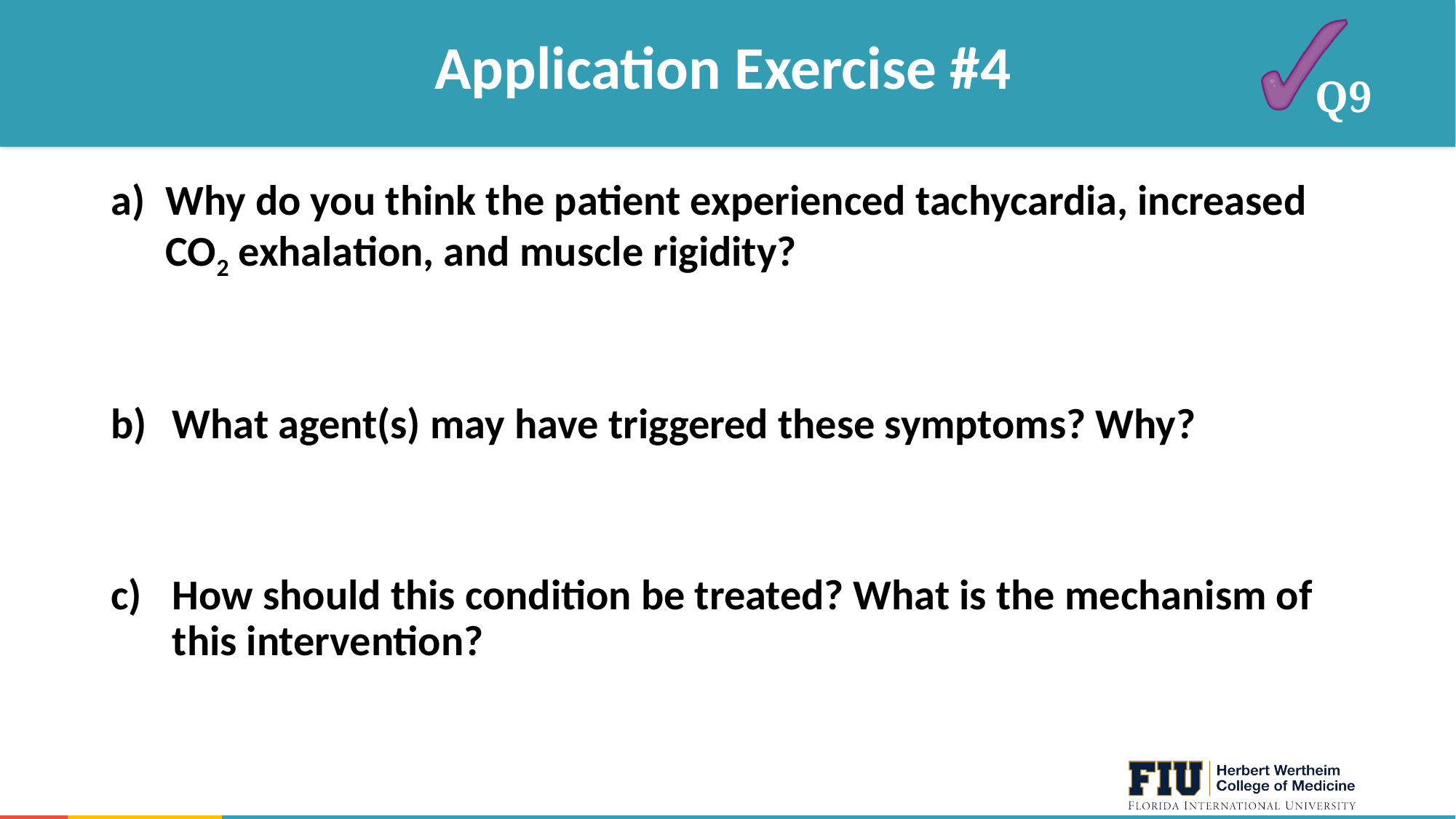

# Application Exercise #4
Q9
Why do you think the patient experienced tachycardia, increased CO2 exhalation, and muscle rigidity?
What agent(s) may have triggered these symptoms? Why?
How should this condition be treated? What is the mechanism of this intervention?

## Slide 36
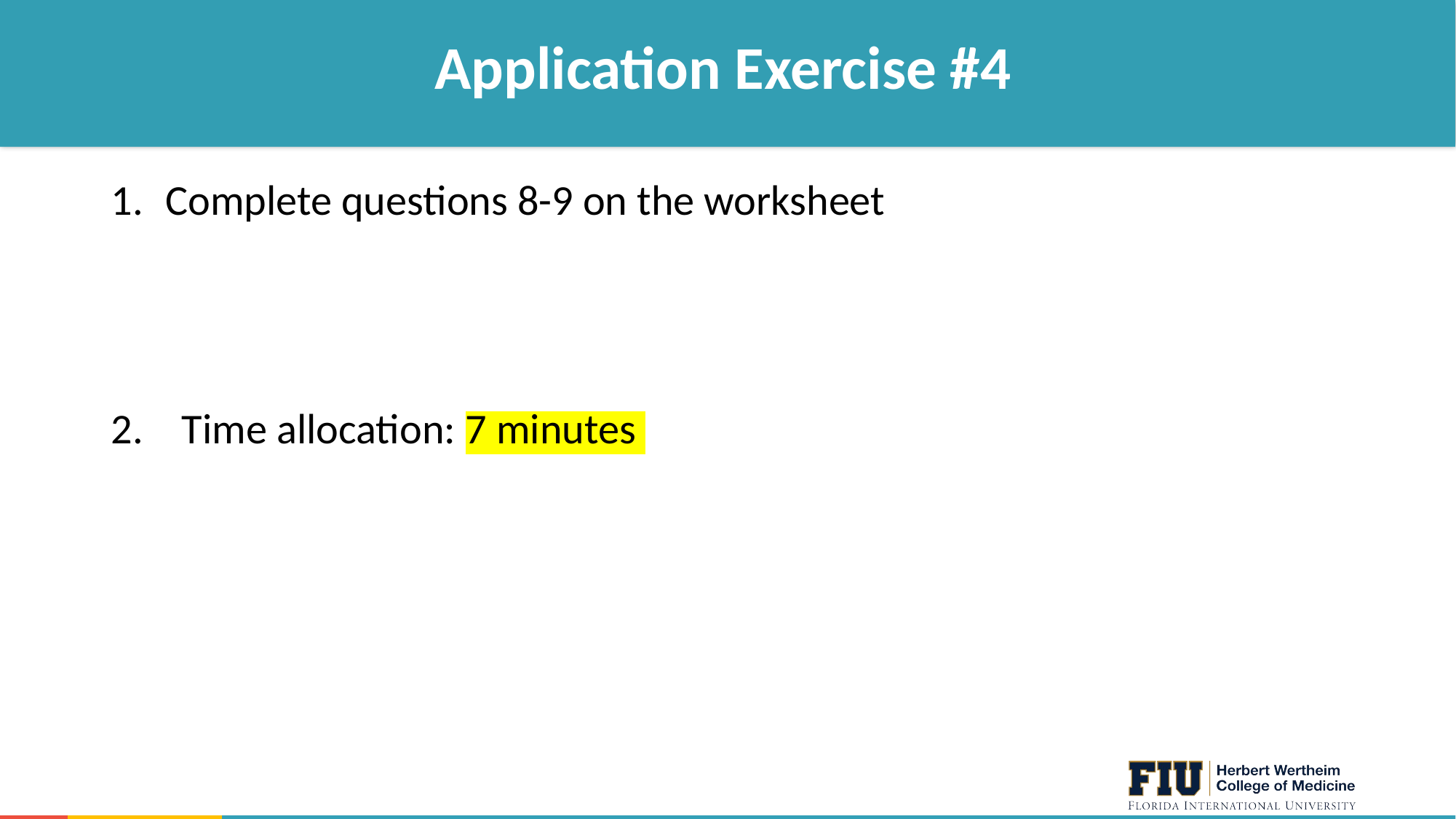

# Application Exercise #4
Complete questions 8-9 on the worksheet
 Time allocation: 7 minutes

## Slide 37
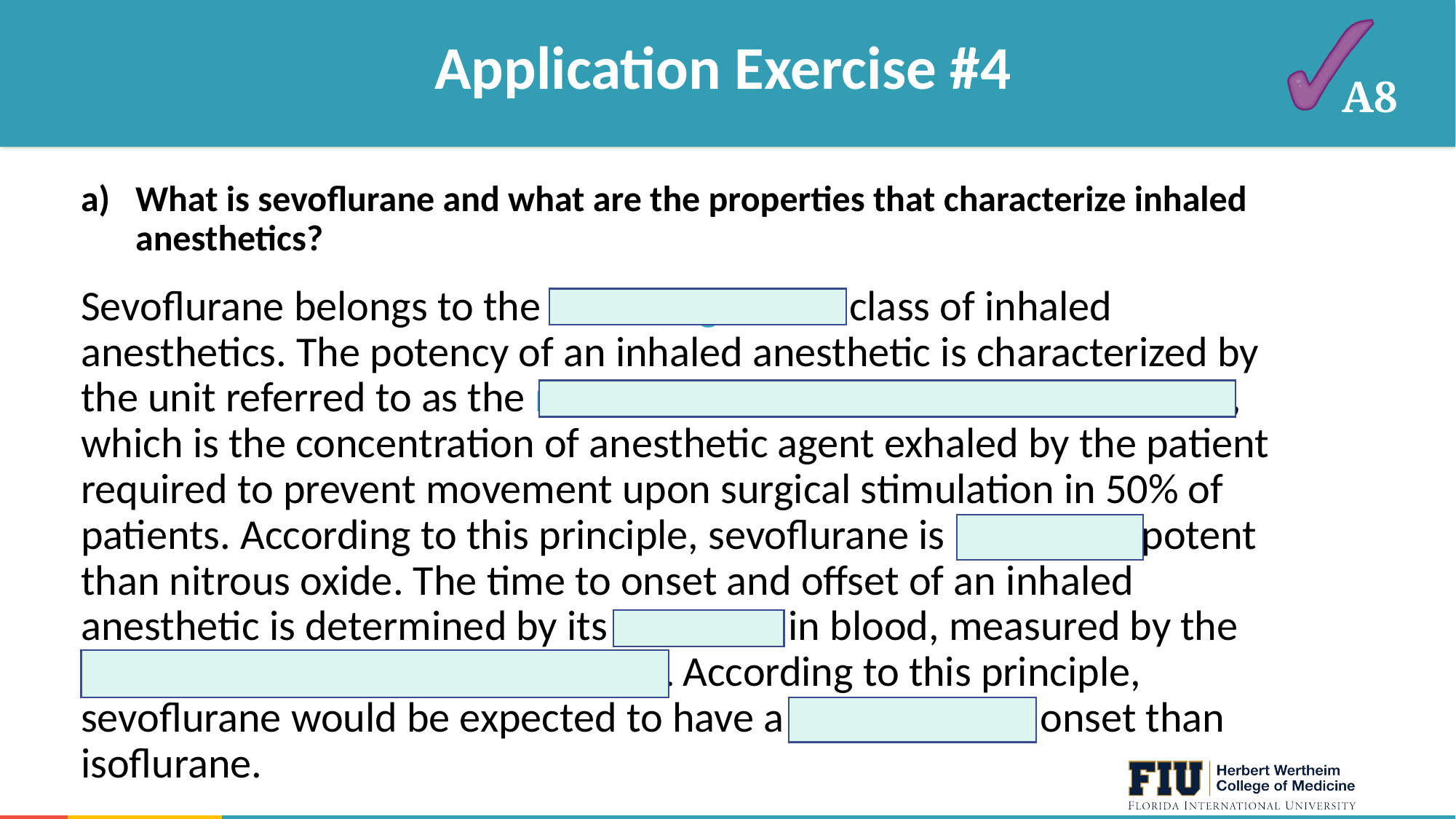

# Application Exercise #4
A8
What is sevoflurane and what are the properties that characterize inhaled anesthetics?
Sevoflurane belongs to the volatile/gaseous class of inhaled anesthetics. The potency of an inhaled anesthetic is characterized by the unit referred to as the minimum alveolar concentration (MAC), which is the concentration of anesthetic agent exhaled by the patient required to prevent movement upon surgical stimulation in 50% of patients. According to this principle, sevoflurane is more/less potent than nitrous oxide. The time to onset and offset of an inhaled anesthetic is determined by its solubility in blood, measured by the blood-gas partition coefficient (λ). According to this principle, sevoflurane would be expected to have a slower/faster onset than isoflurane.

## Slide 38
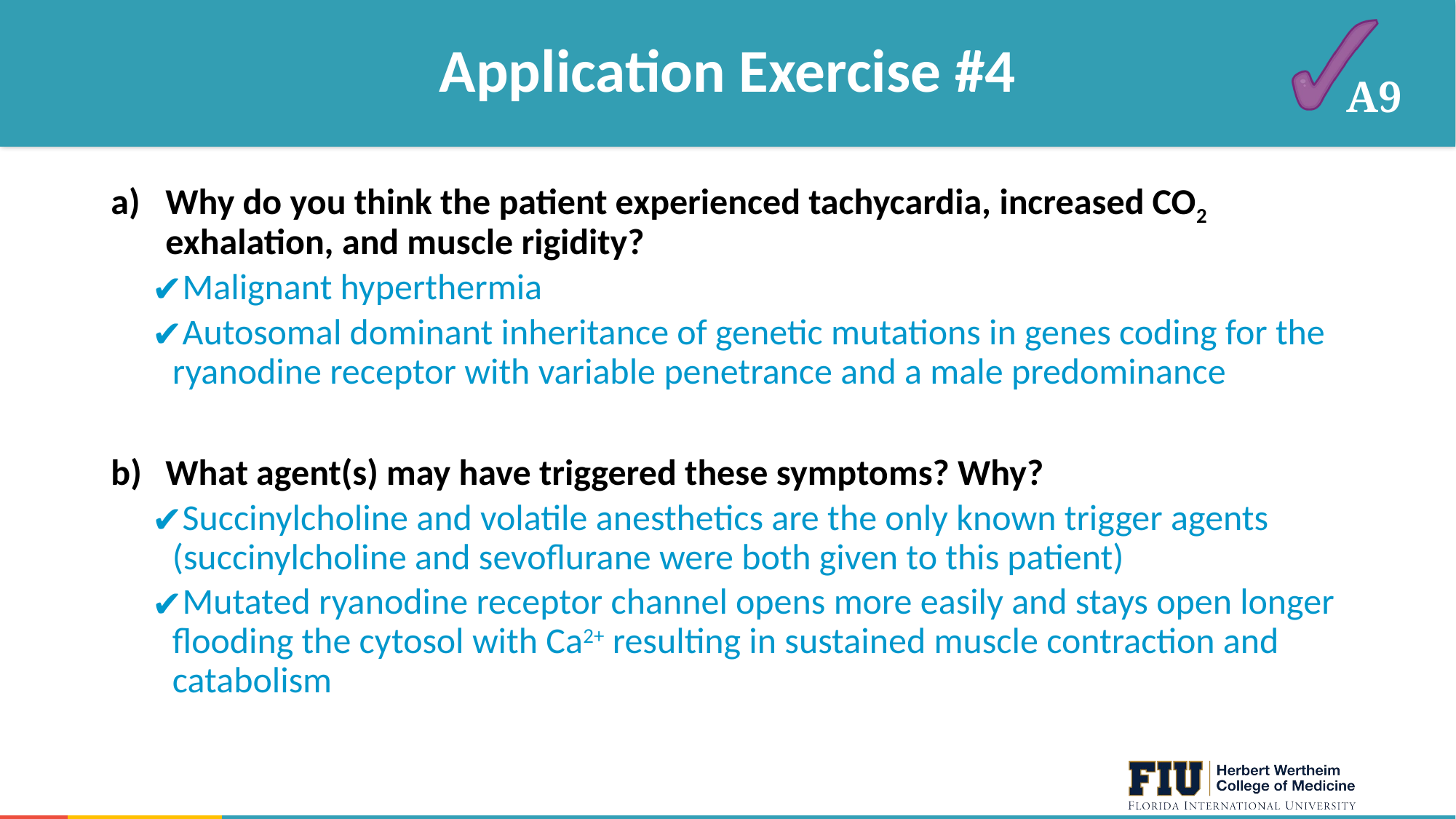

# Application Exercise #4
A9
Why do you think the patient experienced tachycardia, increased CO2 exhalation, and muscle rigidity?
Malignant hyperthermia
Autosomal dominant inheritance of genetic mutations in genes coding for the ryanodine receptor with variable penetrance and a male predominance
What agent(s) may have triggered these symptoms? Why?
Succinylcholine and volatile anesthetics are the only known trigger agents (succinylcholine and sevoflurane were both given to this patient)
Mutated ryanodine receptor channel opens more easily and stays open longer flooding the cytosol with Ca2+ resulting in sustained muscle contraction and catabolism

## Slide 39
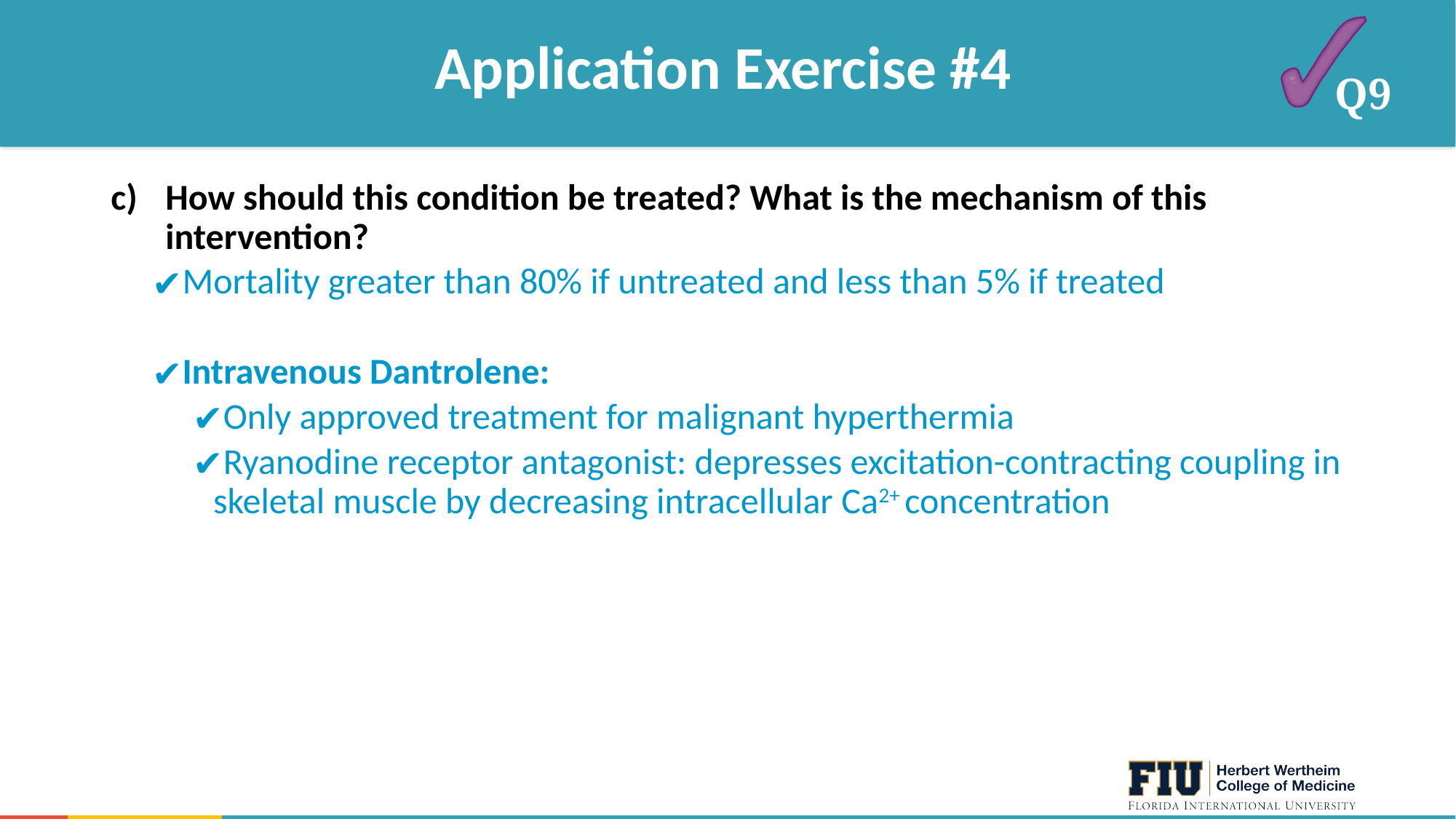

# Application Exercise #4
Q9
How should this condition be treated? What is the mechanism of this intervention?
Mortality greater than 80% if untreated and less than 5% if treated
Intravenous Dantrolene:
Only approved treatment for malignant hyperthermia
Ryanodine receptor antagonist: depresses excitation-contracting coupling in skeletal muscle by decreasing intracellular Ca2+ concentration

## Slide 40
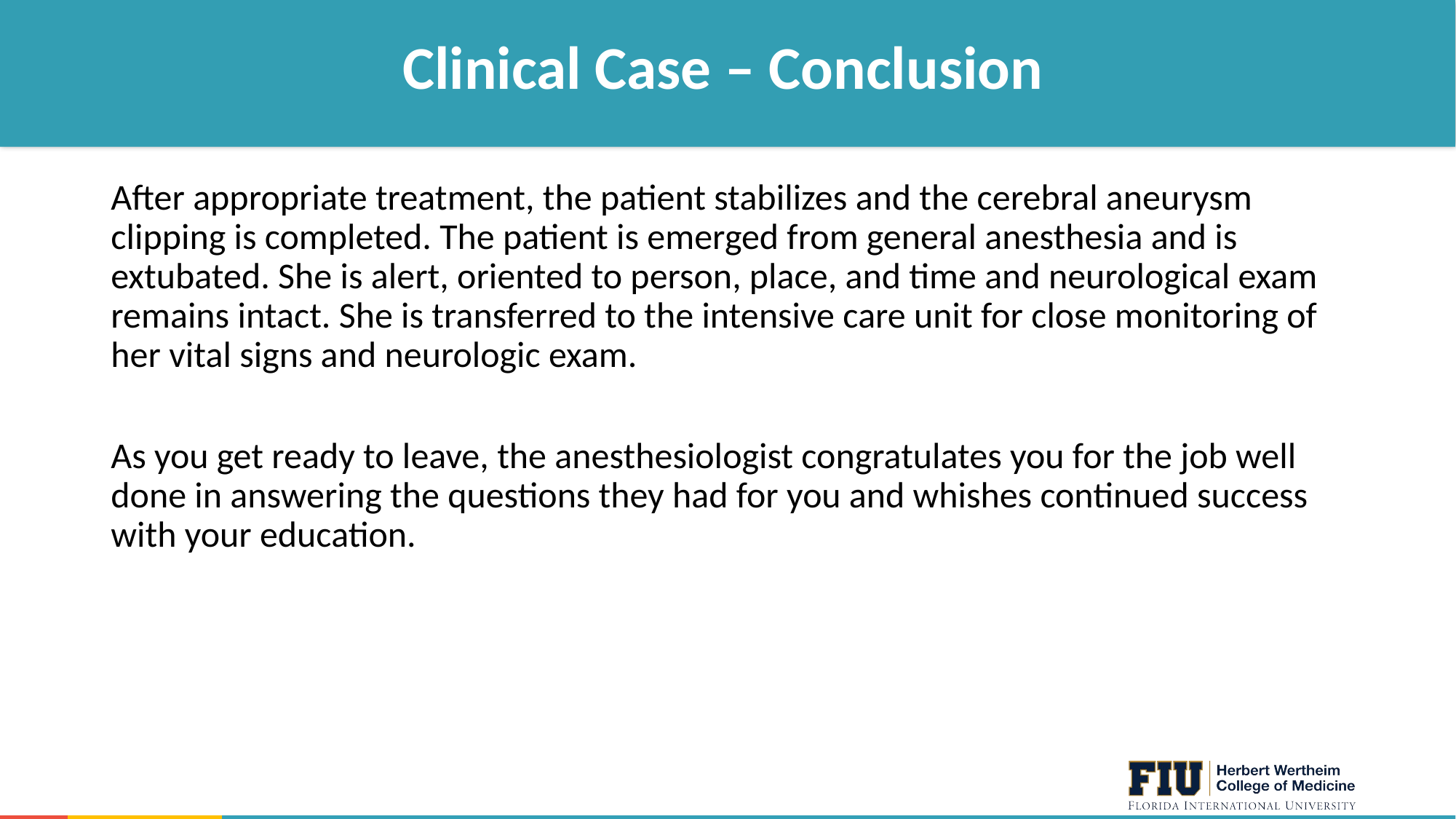

# Clinical Case – Conclusion
After appropriate treatment, the patient stabilizes and the cerebral aneurysm clipping is completed. The patient is emerged from general anesthesia and is extubated. She is alert, oriented to person, place, and time and neurological exam remains intact. She is transferred to the intensive care unit for close monitoring of her vital signs and neurologic exam.
As you get ready to leave, the anesthesiologist congratulates you for the job well done in answering the questions they had for you and whishes continued success with your education.
