# Supplementary material for: Pharmacology of Sedating and Anesthetic Agents: A Case-Based Flipped Classroom Exercise for Preclinical Medical Students
Source: MedEdPORTAL. 2024 Nov 8;20:11462. doi: 10.15766/mep_2374-8265.11462 (PMC11543632; doi:10.15766/mep_2374-8265.11462)
Supplement: Supplementary file 1 — Study Guide.docxPresession Readiness Quiz.docxIn-Class Student Worksheet.docxClinical Case Slides.pptxFacilitator Guide.docxPostsession Consolidation Quiz.docxPostsession Satisfaction Survey.docx [file mep_2374-8265.11462-s001.zip › E. Facilitator Guide.docx]

Facilitator Guide

Pharmacology of Sedating and Anesthetic Agents

This facilitator guide may be used by the facilitator(s) in preparing for the flipped classroom session as well as to guide instruction during the in-person session. This facilitator guide includes step-by-step instructions for the in-class session, including slide numbers for the corresponding PowerPoint presentation, instructions for facilitators, recommended time for each application exercise, probing questions, and review of all correct answers. The slides of the corresponding PowerPoint presentation are designed to comprehensively cover all aspects of the case, revealing it gradually as the session progresses. Yellow highlighted text indicates notes to the facilitator(s) including recommendations for probing questions.

Recommended timing for this session: 2 hours.

## Case Content Cover Sheet

| **Organ System** | Neurology |
| --- | --- |
| **Case** | Sedating and Anesthetic Agents |
| **Clinical Skills / Reasoning** |  |
|  | Gather pertinent information from a history and physical exam |
|  | Recognize role of anesthesia in neurological surgery/general surgery |
|  | Understand medication choices |
|  | Explain medication mechanism of action (MOA) |
|  | Recognize medication side eﬀects/adverse events |
|  | Manage medication side eﬀects/adverse events |
|  | Appreciate pharmacodynamic properties involved in MOA, side eﬀects/adverse events and their management |
| **Interpretation of Studies** |  |
|  | Peripheral nerve stimulation |
| **Community-Engaged** |  |
|  | Anesthetic Rx Safety |
| **Pharmacology** |  |
|  | Lidocaine, Midazolam |
|  | Fentanyl, Propofol |
|  | Succinylcholine |
|  | Rocuronium, Vecuronium |
|  | Pancuronium, Mivacurium |
|  | Atracurium, Cisatracurium |
|  | Physostigmine, Sugammadex |
|  | Pyridostigmine, Neostigmine |
|  | Edrophonium, Sevoﬂurane |
|  | Dantrolene |

## EDUCATIONAL OBJECTIVES

1. Explain the signiﬁcance of minimal alveolar concentration and therapeutic and analgesic indices.
2. Recognize mechanisms of action of inhaled anesthetic agents (i.e., halothane, sevoﬂurane, desﬂurane, sevoﬂurane, and nitrous oxide), and list their physiological eﬀects, clinical uses, and adverse eﬀects.
3. Recognize the mechanisms of action of intravenous anesthetic agents, and list their physiological eﬀects, clinical uses, adverse eﬀects.
4. Recognize the indications, mechanisms of action, and side eﬀects of topical anesthetics.

## EXECUTIVE SUMMARY

This is a case of a 66-year-old woman with new-onset headaches who was recently found to have a large 12mm anterior communicating artery cerebral aneurysm, who presents to the operating room for endovascular cerebral aneurysm coiling performed by a neurosurgeon. Students will be guided through the case as the sedation and anesthetic needs of the patient evolves. The students will work together to solve problems as it pertains to the pharmacology of sedating and anesthetic agents. Speciﬁcally, they will explore the mechanisms of action of various medications, any sub-classiﬁcations within (e.g. local anesthetic: ester-linked vs. amide-linked), and the clinical indications, leading to a discussion on the adverse reactions (common and rare) and their subsequent management.

**GENERAL INSTRUCTIONS**

At the start of the first application exercise, ask each group to identify a “spokesperson” who will respond with answers when the group is called.

When reviewing answers and facilitating discussion in the large group format after each application exercise, randomly call on one group to share their answer. If incorrect answers are presented, ask probing questions to guide students to the correct answers before showing it on the slides. If groups are truly “stuck,” you may encourage them to “phone a friend” and have another group attempt to answer. Encourage discussion if there is uncertainty. Reveal the answer slides only after the students have presented the correct answers in the large group format. For fill in the blank questions, press the space bar to sequentially fill in the blanks on the slides when the slides are viewed in “presenter view.”

**STEP-BY-STEP SLIDE GUIDE**

### Slide 1- Introduction

Introduce the session to the students.

### Slide 2- Clinical Case

A 66-year-old woman with new-onset headaches was recently found to have a large 12mm

anterior communicating artery cerebral aneurysm. She now presents to the operating room for endovascular cerebral aneurysm coiling, in which the neurosurgeon will insert a thin catheter into a groin artery, advance it endovascularly under x-ray guidance into the cerebral vascular

circulation, and a coil will be deployed to block blood ﬂow into the aneurysm.

### Slide 3- Clinical Case - History and Physical Exam

The patient reports no other past medical history and has never previously had surgery. She

does not take any medications at home, but reports having had an allergic reaction to procaine including tongue and throat swelling during a dental procedure. She does not smoke cigarettes and denies use of alcohol or recreational drugs. She reports having been adopted as a child and is unaware of any family history of anesthetic complications.

Pre-procedure vitals:

BP: 133/85 mmHg

HR: 96 beats per minute

RR: 18 breaths per minute

SpO2: 100% on room air

### Physical exam is otherwise unremarkable. She states her blood pressure is usually lower at home and thinks it may be higher today because she is feeling “very nervous.”

### Slide 4 - Clinical Case – Procedure Part 1

After positioning on the procedure table and connecting to standard monitors, the

neurosurgeon injects **lidocaine** subcutaneously in the right groin and the anesthesiologist administers **midazolam** intravenously.

Upon initial skin puncture, the patient reports sharp discomfort at the groin surgical site and additional lidocaine is injected with relief of pain.

Fifteen minutes after starting the procedure, the patient complains of numbness of her lips, ringing in her ears, and shortly thereafter loses consciousness and experiences tonic-clonic convulsions.

You are the medical student assigned to shadow the neurosurgeon and anesthesiologist that day and to answer any questions they may have for you.

### Slides 5-7 Application Exercise #1

Questions on slides #5-6, instructions on slide #7

General Instructions for Students (as shown on slide 7)

Complete questions 1 and 2 on the worksheet

Time allocation: 6 minutes

### Slides 8 and 9 - Q1

Application Exercise 1; 1a: slide #8; 1b: slide #9

1. Why was midazolam used? (What was the anesthesiologist trying to achieve by using this agent?)

Anxiolysis

Moderate sedation

Anterograde amnesia

Short-acting benzodiazepine

Additional Probing Question for Q1a: Ask the large group if anyone considered analgesia as an answer. Stress the point that midazolam does not confer analgesia – **often they have this misconception.**

1. What is the mechanism of action of midazolam? (Please complete the fill in the blank activity to explain your answer.)

**Question stem with answers:**

- Midazolam belongs to the **benzodiazepine** class of drugs. It binds to **GABA_A_** receptors.
- Endogenous ligand **GABA,** the major **(inhibitory**/excitatory) neurotransmitter in the CNS binds to the same receptor. Binding of endogenous ligand to this receptor **(opens/**closes**)** the ion channel facilitating passage of **Cl^-^** ions **(into/**out of**)** the cell. This results in **(hyperpolarization/**depolarization**)** of the nerve cell and **(**increase**/decrease)** neurotransmission by **(inhibiting/**promoting**)** the generation of an action potential.
- Midazolam binds to an **(allosteric/**active**)** site of the same receptor. Midazolam modulates the endogenous pathway by **(increasing/**decreasing**)** the **(frequency/**duration**)** of channel opening. Midazolam is only able to work in the **(presence/**absence**)** of the endogenous ligand.

### Slides 10-13 Q2

Application Exercise 1; 2a-b: slide #10; 2c: slide #11; 2d: slide #12; 2e-f: slide #13

1. Why was lidocaine used? (What was the surgeon trying to achieve by using this agent?)

To numb the local area before incision without loss of consciousness (as opposed to a general anesthetic).

Additional Probing Question for (Q2a): Students often will give the answer as: “lidocaine was used because the patient was allergic to procaine.” That is partially correct but ask them why that class of medication is used here for the patient until all reasons are given.

1. What is the mechanism of action of lidocaine? (Please complete the fill in the blank activity to explain your answer.)

**Question stem with answers:**

- Lidocaine is a local anesthetic belonging to the subclass of Ester**/Amide**-linked local anesthetics. Local anesthetics in this subclass have (one/**two**) “i”(s) in their name, whereas ester-linked local anesthetics have (**one**/two) ‘i’(s) in their name!
- **(**Ionized**/Non-ionized)** lidocaine diffuses readily through the cell membrane whereas **(Ionized/**Non-ionized**)** lidocaine binds to the cytoplasmic (intracellular) side of the **voltage** -gated **Na+**  ion channel and activates**/blocks** it. This **prevents** a transient **increase/**decrease in permeability of the nerve membrane to **Na+** ions, which is required for neuronal transmission. When propagation of action potentials is prevented, sensory impulses cannot be transmitted from the CNS**/periphery** to the **CNS/**periphery.

1. Complete the table for local anesthetics.


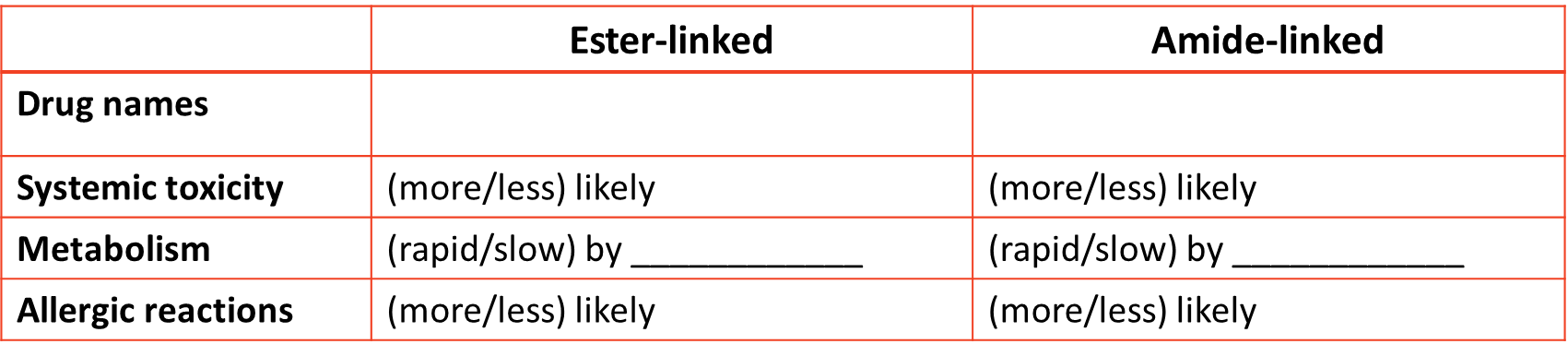


**Drug names for Q2c (not an exhaustive list):**

**Esther linked:**

Benzocaine

Procaine

Chloroprocaine

Tetracaine

Cocaine

**Amide-Linked**

Bupivacaine

Prilocaine

Lidocaine

Ropivacaine

Mepivacaine

**Systemic toxicity:**

Esther linked: Less likely

Amide-Linked: More likely

**Metabolism:**

Esther linked: **Rapid by plasma cholinesterase**

Amide-Linked: **Slow, by hepatic metabolism**

Additional Probing Questions for (Q2c): Ask why they think ester-linked are less likely to cause systemic toxicity. Ester-linked are less likely to persist in the circulation long enough to cause toxicity due to rapid metabolism. However, amide-linked may persist in the systemic circulation for a longer time because they are metabolized by the liver and their clarence from the body is slower.

**Allergic reactions:**

Esther linked: More likely (PABA derivatives)

Amide-Linked: Less likely (very rare)

1. What properties of this agent make it a specifically good choice for this patient?

Patient had an allergy to the ester-linked local anesthetic procaine. Lidocaine is amide-linked and considered acceptable for use in this scenario. It is also a short-acting local anesthetic, which is suitable for this procedure. In contrast, if you chose ropivacaine or bupivacaine which is in the longer end of spectrum, the patient may experience analgesia in the area for up to 12+ hours, which is unnecessary and potentially unsafe.

1. Why do you think the patient experienced perioral numbness, tinnitus, and a seizure?

Local anesthetic systemic toxicity (LAST) – can discuss differential diagnosis. Discuss the order of symptom propagation, which aids in diagnosis clinically.

1. What can be done to treat this condition? How could this have been prevented?

Lipid emulsion and supportive care such as additional benzodiazepine to treat seizure.

20% lipid emulsion (“Lipid rescue therapy”) and supportive care (e.g., *additional* benzodiazepine for seizure control, etc.)

Careful aspiration of the syringe before injection of local anesthetics can help reduce the risk of LAST. If the syringe aspirates blood, the tip of the needle is likely inappropriately inside of a blood vessel and should be immediately redirected as injection of the local anesthetic into the blood vessel greatly increases the risk of LAST and will not have the desired local analgesic effect.

Additional Probing Question: Ask how epinephrine would limit the systemic toxicity. Epinephrine constricts the blood vessels and decreases the blood supply to the area. Less blood supply, less absorption of lidocaine into the systemic circulation.

**Slide 14- Clinical Case – Procedure Part 2**

After appropriate treatment for LAST and additional midazolam for seizure control, the procedure resumes. After several attempts at coiling the artery, the neurosurgeon informs you that she is unable to achieve complete isolation of the aneurysm endovascularly. The procedure will need to be converted to an open craniotomy for aneurysm clipping under general anesthesia with endotracheal intubation.

For induction of general anesthesia and endotracheal intubation, the anesthesiologist plans to administer **fentanyl, propofol,** and **succinylcholine**.

### Slides 15-18- Application Exercise #2

Questions on slides #15-17, instructions on slide #18, and answers on slides #19-24)

General Instructions for Students (as shown on slide 18)

Complete questions 3, 4 and 5 on the worksheet

Time allocation: 7 minutes

### Slide 19- Q3

Application Exercise 2; 3a

1. Why are each of the following medications used when inducing general anesthesia? (What was the anesthesiologist trying to achieve by using each agent?)
   1. Fentanyl
   2. Propofol
   3. Succinylcholine

**Fentanyl**

Analgesia - Pain relief for tracheal intubation and surgical stimulation

**Propofol**

Sedation/hypnosis - Loss of consciousness, lack of awareness, prevention of recall **(no analgesia!)**

**Succinylcholine**

Muscle relaxation - “Paralysis” facilitating tracheal intubation and/or preventing movement to surgical stimuli **(no analgesia!)**

Additional Probing Question: Ask the large group if anyone considered analgesia as an answer. Stress the point that propofol and succinylcholine do not provide analgesia -**often times they have this misconception.**

### Slide 20- Q4

1. What are the potential adverse reactions of propofol that the anesthesiologist must consider in this short-term setting?

Respiratory depression/arrest!

Hypotension from vasodilation and cardiac depression

Pain at injection site

1. What are the potential adverse reactions if propofol were to be used for sedation over several days?

Propofol infusion syndrome - Muscle breakdown (rhabdomyolysis), metabolic acidosis, kidney failure, cardiac failure

Green urine- if students mention green urine, remind them that this is a side effect and not an adverse reaction.

**Note to facilitators**: Propofol infusion syndrome - happens more commonly in children.

### Slides 21-24 Q5

Application Exercise 2; 5a: slides #21-23; 5b: slide #24

1. How does the mechanism of action differ between the muscle relaxants succinylcholine and rocuronium? (Please complete the fill in the blank activity to explain your answer.)

**Question stem with answers:**

Neuromuscular junctions (NMJs) have **nicotinic/**muscarinic ACh receptors. These receptors are **ligand-**gated ion channels that open in response to binding of ACh (endogenous ligand) to facilitate influx of **Na^+^** ions into the muscle cell (Ca2+ can also be accepted as correct if given IN ADDITION to Na+). **Na^+^** ion influx dominates the exchange, and the membrane **depolarizes**, generating a motor endplate potential. Therefore, ACh binding to these receptors initiates muscle **contraction.** Endogenous ACh is quickly degraded by **acetylcholinesterase (AChE**) in the synaptic cleft, which results in rapid termination of its action and depolarization**/repolarization** of the membrane.

Succinylcholine structurally resembles the **ACh** molecule and acts as a nicotinic ACh receptor **agonist/**antagonist. Succinylcholine is **resistant** to degradation by AChE, which allows it to continue stimulating the receptor. This first causes opening of the **Na+ ion** channel associated with nicotinic receptors, which results in **depolarization/**repolarization (phase I block). This leads to a transient body wide twitching of muscles known as **fasciculations.**

Rocuronium is a competitive agonist**/antagonist** at the nicotinic ACh receptor. Rocuronium competes with the endogenous ligand **ACh** at the receptor and promotes**/prevents** binding of the endogenous ligand to its receptor. This prevents **depolarization/**repolarization of the muscle cell membrane and inhibits development of an endplate potential therefore preventing muscular contraction (muscle movement). Agents such as rocuronium are depolarizing**/non-depolarizing** muscle relaxants.

Rocuronium’s competitive action can be overcome by administration of **cholinesterase inhibitors**, such as neostigmine, which increases the concentration of the endogenous ligand ACh in the NMJ. Clinicians employ this strategy to **shorten/**prolong or “reverse” the duration of neuromuscular blockade.

**Note to Facilitator**: Non-depolarizing agents, such as rocuronium, act as competitive antagonists at the neuromuscular junction. Therefore, to reverse non-depolarizing agents, you want to increase the amount of endogenous acetylcholine that can competitively overcome and displace the competitive antagonist. Acetylcholinesterase is the enzyme that breaks down acetylcholine in the neuromuscular junction. To increase the amount of acetylcholine you may want to inhibit acetylcholinesterase, leading to less degradation and increased concentration of acetylcholine, which is one option for reversal of the effects of non-depolarizing neuromuscular blocking agents.

1. What are the potential adverse reactions of succinylcholine that the anesthesiologist must consider? What patient characteristics (not present in this patient) would have contraindicated its use?

**Adverse reactions:**

Post-operative myalgias (muscle aches)

Masseter muscle spasm

Triggering agent for malignant hyperthermia

Potassium release during depolarization 🡪 hyperkalemia

**Contraindications:**

Conditions associated with chronic decrease in ACh release cause an increase in the number of extrajunctional ACh receptors, which can result in an exaggerated release of potassium when activated (i.e. profound hyperkalemia) and possibly cardiac arrest! Examples of these conditions include burn injuries, massive trauma/crush injuries, spinal cord injury, stroke, Guillain-barre syndrome, prolonged immobilization, myopathies [e.g., Duchenne’s muscular dystrophy])

Personal or family history of malignant hyperthermia

**Note to facilitators regarding the contraindications:**

**Ask the students for a rationale why these conditions are contraindicated.**

A good way to think about it is any patient who may have up regulation of ACh receptors- anything that prevents movement (like spinal cord injuries, long term immobilization, muscular pathologies such as Duchene muscular dystrophy), etc. The body’s response to a lack of muscle contraction is to upregulate/increase extrajunctional ACh receptors in order to attempt to trigger contraction. Therefore, patients who have experienced long-term immobilization have increased expression of ACh receptors. Succinylcholine, a depolarizing agonist at the ACh receptor, normally causes some degree of potassium release from the receptor in normal patients. This potassium release can normally be tolerated by the patient. However, patients who have an upregulation of the ACh receptors will have an exaggerated amount of potassium release. This can then cause hyperkalemia, which can lead to clinically significant arrhythmias or cardiac arrest.

Succinylcholine is a triggering agent for malignant hyperthermia, which would also be a contraindication.

**Slide 25- Clinical Case - Part 3**

After administering fentanyl and propofol, the patient immediately loses consciousness and stops breathing. The anesthesiologist evaluates the patient’s response to train-of-four peripheral nerve stimulation, with the response demonstrated by figure (A) in the image below.

Succinylcholine is administered and the patient experiences visible muscle fasciculations. Upon cessation of the fasciculations, the response to train-of-four is evaluated again, represented by figure (B). The anesthesiologist successfully intubates the patient’s trachea with a breathing tube and initiates mechanical ventilation.


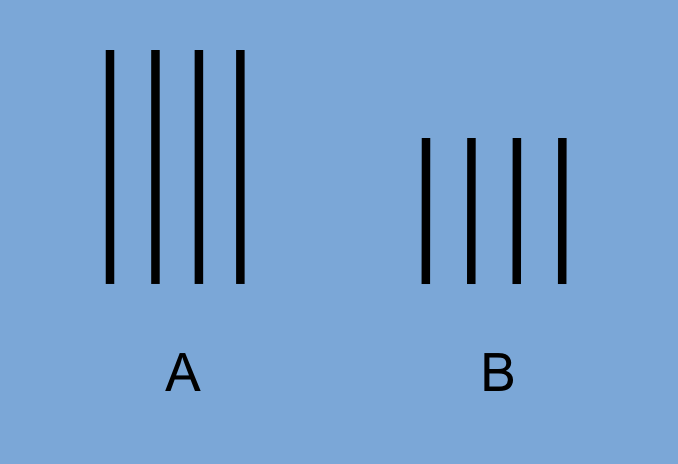


### Slides 26-29 Application Exercise #3

Questions on slides #26-28, instructions on slide #29

General Instructions for Students (as shown on slide 29)

Complete questions 6 and 7 on the worksheet

Time allocation: 7 minutes

### Slide 30 Q6

Application Exercise 3; 6a-b: slide #30

1. How is a train-of-four response evaluated? Why did the anesthesiologist wish to evaluate it?

Train-of-four: pattern of peripheral nerve electrical stimulation with a series of four twitches over 2 seconds. The first stimulation was performed to evaluate a baseline and second to confirm adequate neuromuscular blockade prior to intubation. A third stimulation should be performed later to confirm appropriate recovery from blockade.

Additional Probing Question: Before you go to part B, ask them at what time points you would check for the train-of-four response to be evaluated (if you are the anesthesiologist) for this procedure?

1) Before the administration of succinylcholine: this is to measure the baseline response. Each patient's baseline amplitude of response is different (depending on patient physiology in addition to variability in equipment placement, etc.)

2) After administering the succinylcholine: to confirm adequate muscle blockade evidenced by the decrease in amplitude before the desired procedure is performed (usually endotracheal intubation).

3) Lastly, evaluate the TOF after the effects of succinylcholine are expected to have worn off, confirming that the muscle blockade is no longer in place, evidenced by a return to baseline. The patient needs to return to their baseline muscle strength to breath on their own and avoid respiratory compromise. Additionally, you would be risking waking up the patient before they are **UN-paralyzed (resulting in significant psychologic distress).**

1. How would the train-of-four response have differed if rocuronium had been given instead of succinylcholine?


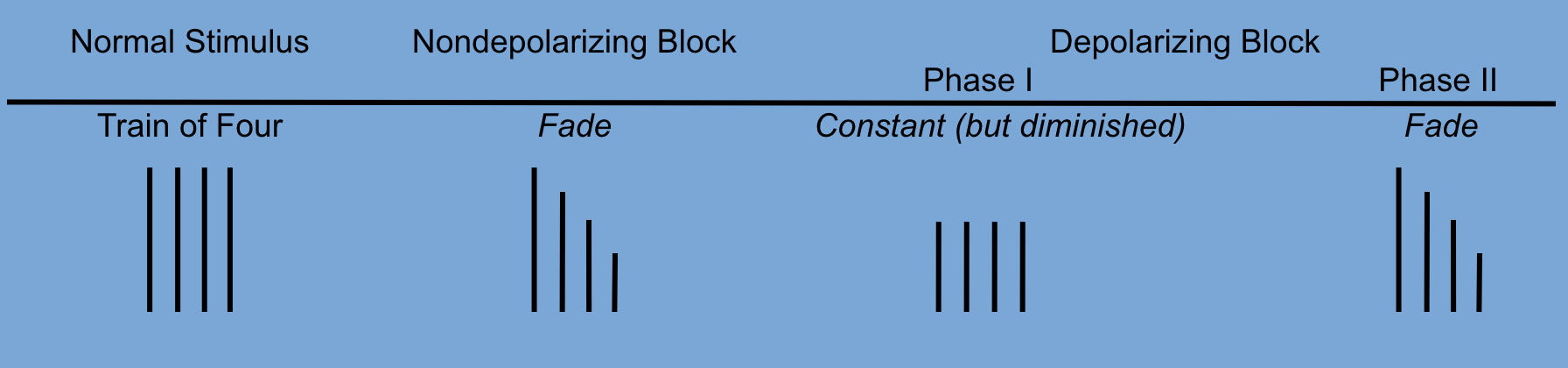


**Image explanation:**

Normal Stimulus: A normal stimulus should have four bars of equal amplitude, representing a normal response to each of the four stimuli.

Nondepolarizing block: Each subsequent stimulation should produce a response (bar) of lesser amplitude than the one coming before it. At deep levels of blockade, the final (or potentially all) bars may fade to an extent that they disappear entirely, leading to fewer than 4 (or no) bars total.

Depolarizing block (phase 1): A phase one block is the expected response following administration of succinylcholine. All stimulations should result in a response of equal amplitude but diminished from the baseline normal response.

Depolarizing block (phase 2): A phase two block demonstrates fade in amplitude of each subsequent stimulation, similarly to what is seen with non-depolarizing agents. This response is only likely to be seen when succinylcholine is administered at higher than usual doses or as a prolonged infusion. At standard doses, succinylcholine would be expected to produce a phase 1 block only and would NOT be expected to progress to a phase 2 block but rather would return to baseline after the succinylcholine is rapidly metabolized. While a phase 2 blocks is not the typically expected response and may not be commonly tested at the medical student level, we chose to include it in the diagram for the sake of completeness as it is commonly included in pharmacology textbooks and may be encountered by students in their studies.

### Slides 31 and 32- Q7

Application Exercise 3; 7a-b: slide #31; 7c: slide #32

1. Will this patient require reversal of neuromuscular blockade? Why or why not?

No. This patient received the depolarizing agent succinylcholine, which will be rapidly metabolized by pseudocholinesterases and therefore will not require reversal. In fact, cholinesterase inhibitors prolong rather than reverse a typical phase I block by a depolarizing agent (succinylcholine). No approved reversal agent exists for succinylcholine.

1. Would your answer change if the patient had received rocuronium instead of succinylcholine?

Non-depolarizing agents often require reversal to resume normal neuromuscular function unless full recovery is documented by peripheral nerve monitoring. Incomplete recovery, referred to as “residual neuromuscular blockade” or “post-procedural paralysis”, is associated with significant morbidity often in the form of respiratory distress, reintubation, and psychological distress.

1. Complete the table below for the two classes of medications used to reverse neuromuscular blockage by non-depolarizing neuromuscular blocking agents.

|  | **Cholinesterase Inhibitors** | **Selective Relaxant Binding Agent** |
| --- | --- | --- |
| **Drug names (generic)** |  |  |
| **Muscle relaxants reversed by this class of drugs** |  |  |
| **Mechanism** |  |  |
| **Adverse reactions** |  |  |

**Drug names**

**Cholinesterase Inhibitors**

Neostigmine

Pyridostigmine

Physostigmine

Endrophonium

**Selective Relaxant Binding Agent**

Sugammadex

**Muscle relaxants reversed by cholinesterase inhibitors**

Rocuronium

Vecuronium

Pancuronium

Mivacurium

Atracurium

Cisatracurium

**Muscle relaxants reversed by the selective relaxant binding agent sugammadex:**

Rocuronium

Vecuronium

**Mechanism of cholinesterase inhibitors:** Inactivates acetylcholinesterase indirectly increasing the amount of ACh available to compete with the NDMR.

**Mechanism of Sugammadex:** Binds the NDMR forming a complex rendering the NDMR unable to bind with acetylcholine receptors.

Note to the facilitator: Sugammadex is becoming increasingly popular but is only able to be used with rocuronium and vecuronium because it selectively binds to them. It forms nonreversible bonds and leads to the excretion of these medications in the urine. Therefore, sugammadex is not recommended for use in patients with renal failure.

Of note, mivacurium and pancuronium are no longer in regular clinical use in the United States currently. However, we chose to include them due to their unique and occasionally tested characteristics (mivacurium is metabolized by plasma cholinesterases and pancuronium has a vagolytic effect).

**Adverse reactions of cholinesterase inhibitors:**

Profound bradycardia, bronchospasm, increased secretions (salivation), increased peristaltic activity causing postoperative nausea, vomiting, and fecal incontinence/diarrhea.

(Think of too much acetylcholine stimulation on **muscarinic receptors** throughout the body). What we are trying to do is increase the ACh effect in the nicotinic receptors at the neuromuscular junction such that our endogenous ACh is in abundance and can compete with the NDMRs and re-establish normal muscle contraction. By doing this we risk increasing ACh not only accumulating in the neuromuscular junction where we need the effect but also elsewhere in the body. ACh works on two types of receptors (nicotinic and muscarinic). Elsewhere in the body what receptors do we have that ACh can act upon? Muscarinic receptors, and that is why we end up with diarrhea, bronchospasm, salivation, bradycardia etc.

Additional Probing Question:

So, if we are expecting these side effects when using this reversal agent, what can we do to minimize or prevent these side effect? What would you want to administer? Muscarinic antagonist such as atropine or glycopyrrolate. These are two medications widely used clinically to minimize these unwanted effects.

**Adverse reactions of sugammadex:**

Decreased efficacy of oral contraceptives (for up to 7 days) and a small risk of bradycardia or anaphylaxis.

### Slide 33- Clinical Case – Procedure Part 4

The inhaled volatile anesthetic sevoflurane is now administered to the patient via the endotracheal breathing tube. The anesthesiologist indicates to the neurosurgeon that she may proceed with surgery.

Partway through the procedure, the patient begins to experience tachycardia, increased CO_2_ exhalation, and muscle rigidity.

### Slides 34-36 Application Exercise #4

Questions on slides #34-35, instructions on slide #36

General Instructions for Students (as shown on slide 36)

Complete questions 8 and 9 on the worksheet

Time allocation: 7 minutes

### Slide 37- Q8

Application Exercise 4; 8a: slide 37

1. What is sevoflurane and what are the properties that characterize inhaled anesthetics? (Please complete the fill in the blank activity to explain your answer.)

Sevoflurane belongs to the **volatile/**gaseous class of inhaled anesthetics. The potency of an inhaled anesthetic is characterized by the unit referred to as the **minimum alveolar concentration (MAC)**, which is the concentration of anesthetic agent exhaled by the patient required to prevent movement upon surgical stimulation in 50% of patients. According to this principle, sevoflurane is **more/**less potent than nitrous oxide. The time to onset and offset of an inhaled anesthetic is determined by its **solubility** in blood, measured by the **blood-gas partition coefficient (λ)**. According to this principle, sevoflurane would be expected to have a slower**/faster** onset than isoflurane.

**Slides 38 and 39- Q9**

Application Exercise 4; 9a-b: slide #38; 9c: slide #39

1. Why do you think the patient experienced tachycardia, increased CO_2_ exhalation, and muscle rigidity?

Malignant hyperthermia is an autosomal dominant genetic condition in which patients experience hypermetabolism upon exposure to succinylcholine or volatile anesthetics. Oftentimes, these patients will have had family members who have had a complication while undergoing anesthesia. Due to variable penetrance, some patients with a mutation causing malignant hyperthermia may never experience malignant hyperthermia or they may not experience it on their first exposure but may experience it on subsequent exposures. These patients often have a mutation in the RYR1 gene, leading to increased frequency and duration of calcium channel opening. This uncontrolled release of calcium from the sarcoplasmic reticulum of skeletal muscle leads to sustained muscle contraction (muscle rigidity), which depletes ATP. The depletion of ATP increases oxygen consumption, carbon dioxide production (increased CO2 seen in this patient), and heat. This increased oxygen demand and carbon dioxide production results in tachycardia as an attempt to compensate.

1. What agent(s) may have triggered these symptoms? Why?

Succinylcholine and volatile anesthetics (gaseous anesthetics such as nitrous oxide are not triggering agents for malignant hyperthermia).

1. How should this condition be treated? What is the mechanism of this intervention?

Mortality is greater than 80% if untreated and less than 5% if treated. Stress the importance of timely management of this condition. All operating rooms where triggering agents for malignant hyperthermia (succinylcholine or volatile anesthetics) are available should also have a dedicated cart with dantrolene to be immediately available should a case occur.

**Treated with intravenous Dantrolene:**

Only approved treatment for malignant hyperthermia

Ryanodine receptor antagonist- Depresses excitation-contracting coupling in skeletal muscle by decreasing the intracellular Ca2+ concentration

Dantrolene acts directly on the ryanodine receptor that became stuck open by one of these agents (succinylcholine or volatile anesthetics) and it antagonizes that receptor.

**Note to facilitators:** Dantrolene is available as IV as well as oral tables. Intravenous is the only option to treat malignant hyperthermia. However, can consider probing the students to see if anyone can contribute the list of conditions that oral dantrolene is used to treat. It is used as an anti-spasticity agent to relax the muscles in patients with neurologic conditions that have resulted in chronic spasticity (multiple sclerosis, prior stroke, cerebral palsy, etc.)

### Slide 40- Clinical Case – Conclusion

After appropriate treatment, the patient stabilizes and the cerebral aneurysm clipping is completed. The patient is emerged from general anesthesia and is extubated. She is alert, oriented to person, place, and time and neurological exam remains intact. She is transferred to the intensive care unit for close monitoring of her vital signs and neurologic exam.

As you get ready to leave, the anesthesiologist congratulates you for the job well done in answering the questions they had for you and whishes continued success with your education.
