# Supplementary material for: Pharmacology of Sedating and Anesthetic Agents: A Case-Based Flipped Classroom Exercise for Preclinical Medical Students
Source: MedEdPORTAL. 2024 Nov 8;20:11462. doi: 10.15766/mep_2374-8265.11462 (PMC11543632; doi:10.15766/mep_2374-8265.11462)
Supplement: Supplementary file 1 — Study Guide.docxPresession Readiness Quiz.docxIn-Class Student Worksheet.docxClinical Case Slides.pptxFacilitator Guide.docxPostsession Consolidation Quiz.docxPostsession Satisfaction Survey.docx [file mep_2374-8265.11462-s001.zip › _Educational Summary Report_11462.pdf]

MedEdPORTAL® The AAMC Journal of Teaching and Learning Resources

Original Publication

Open Access

Pharmacology of Sedating and Anesthetic Agents: A Case-Based Flipped Classroom Exercise for Preclinical Medical Students

Daniella Nunez, Richard I. Suarez, JM, Melanie Molina, Gagani Athauda, MD, Rebecca L. Toonkel, MD, Jenny Fortun, PhD, Nicholas V. Mendez, MD*

*Corresponding author: nicholas.mendez@ucsf.edu

Abstract

Introduction: Sedating and anesthetic drugs are widely used in clinical practice; however, relevant teaching remains underrepresented in undergraduate medical education. We developed a 2-hour flipped classroom activity integrating foundational science topics, evidence-based medicine, and clinical reasoning on anesthetic pharmacology for preclinical medical students. **Methods:** Presession, second-year medical students reviewed a study guide and completed a readiness assessment. The flipped classroom session was facilitated in a large-group format with learners in small groups. At session end, students completed a consolidation quiz. Two case-relevant questions were included on the midterm and one on the final exam. Student satisfaction was assessed through an anonymous postsession survey. **Results:** One hundred ten students participated in the session. Mean performance on the readiness assessment was 96%. Mean performance on the postsession quiz was also 96%. Mean performance on the three midterm and final exam questions was higher than the national mean (94% vs.72%, $p < .005$). Seventy-six students (69%) completed the survey, with mean satisfaction of 4.6 ($SD = 0.7$) on a 5-point Likert scale (1 = *Strongly Disagree,* 5 = *Strongly Agree*). **Conclusion:** We developed a flipped classroom session teaching pharmacology of sedating and anesthetic drugs for preclinical medical students. Students performed well on pre- and postsession assessments and above the national mean on National Board of Medical Examiners questions, suggesting adequate knowledge acquisition. This session was found to be a highly satisfactory and effective teaching tool requiring students to integrate foundational and clinical science knowledge.

Keywords
Anesthetics, Sedation, Anesthesiology, Case-Based Learning, Critical Care Medicine, Flipped Classroom, Pain Medicine, Pharmacology & Toxicology

Educational Objectives

By the end of this session, learners will be able to:

1. Classify anesthetic drugs based on mechanism of action and explain indications, unique properties, adverse reactions, and contraindications.
2. Select appropriate anesthetic drugs based on a patient's clinical presentation and individual characteristics, as well as desired therapeutic effects and pharmacologic properties.
3. List potential side effects associated with anesthetic drugs and explain strategies to minimize, prevent, and/or treat them.
4. Contrast amide- and ester-linked local anesthetics based on their metabolism, potential for systemic toxicity, and allergic reactions.
5. Distinguish depolarizing and nondepolarizing neuromuscular blocking agents and explain methods for monitoring and reversing neuromuscular blockade.
6. Recognize the clinical presentation of malignant hyperthermia, list triggering agents, and recommend a treatment plan.

Citation:
Nunez D, Suarez RI, Molina M, et al. Pharmacology of sedating and anesthetic agents: a case-based flipped classroom exercise for preclinical medical students. *MedEdPORTAL.* 2024;20:11462.
https://doi.org/10.15766/mep_2374-8265.11462

Introduction

The practice of modern medicine and surgery has been made possible by the development of analgesics, sedating agents, and anesthetics, with an estimated 40-50 million surgical procedures

Copyright © 2024 Nunez et al. This is an open-access publication distributed under the terms of the Creative Commons Attribution license.

being performed annually in the United States alone.[1] Outside of the perioperative arena, analgesics and sedating agents are employed in a wide variety of areas including both outpatient and inpatient settings, from the care of patients with chronic pain to those in critical condition. As a result, the pharmacology of sedating and anesthetic agents is a critical component of undergraduate medical education and is a core competency for every physician regardless of chosen specialty. Additionally, the American College of Clinical Pharmacology has highlighted the severe lack of in-depth training in clinical pharmacology during medical school and has encouraged the implementation of sessions directed at teaching the clinical knowledge needed by medical students when entering their first year of residency training.[2,3]

Accordingly, the properties of some sedating and anesthetic agents are included in the *USMLE Content Outline.*[4] Despite this, formal preclinical teaching of these drugs remains inconsistent and largely underrepresented in medical education across the United States.[5] While some educational materials relevant to anesthetic pharmacology are available for resident teaching, a search of *MedEdPORTAL,* Google Scholar, and PubMed for medical education materials yielded limited results for resources geared towards preclinical medical students.[5-7] There is no consensus on how clinical anesthesiology should be taught to medical students, with most institutions teaching aspects of sedative and anesthetic pharmacology in a fragmented fashion throughout the first 2 years or during the clerkship years.[5] Some medical schools completely lack any formal required training in anesthesiology.[5,8] At institutions where it is taught, most preclinical sessions on sedating and anesthetic agents are conducted in a traditional lecture format without the opportunity to translate the knowledge into clinical application.[5]

Over the past 2 decades, in keeping with recommendations from the Carnegie Foundation for the Advancement of Teaching, medical education has transitioned away from traditional didactic lectures towards active learning modalities with an emphasis on clinical reasoning and application of knowledge.[9,10] The flipped classroom (FC) approach to learning facilitates integration of foundational knowledge into an applied team-based and case-based discussion.[9] In an FC session, students use preassigned educational materials to engage in learning outside of the classroom and acquire baseline foundational knowledge before coming to class. The in-class session is then used to facilitate discussion and problem-solving where students apply their knowledge in conjunction with guidance from the facilitator. This face-to-face active learning time is meant to explore more complex concepts and address knowledge gaps from the presession independent study.[9] Medical students in both the preclinical and clinical years have reported a strong positive perception of FC activities, indicating that these activities increase their engagement and interest in learning.[11] By encouraging students to self-direct and actively engage in their own learning, the FC session is an active learning strategy that aims to foster long-term learning and encourage the refinement of self-directed learning skills.[11,12] Additionally, FC activities have been shown to yield positive academic outcomes and improve learner satisfaction.[13]

Since our review of the literature revealed no published active learning tools integrating foundational science topics, evidence-based medicine, and clinical reasoning skills around anesthetic pharmacology, we developed an FC activity designed to facilitate active learning about this topic and address this gap in the medical education curriculum. We devised a case-based FC activity focusing on the pharmacology of sedating and anesthetic agents, including their indications and adverse effects, with an emphasis on the development of clinical reasoning and application skills. Using a clinical scenario, second-year medical students were tasked with making clinical decisions based on their knowledge of foundational science concepts relevant to sedative and anesthetic pharmacology.

Methods

This case-based FC session covering the pharmacology of sedating and anesthetic drugs was designed for second-year medical students in the 2022 academic year ($n = 110$) as part of the 6-week Nervous System and Behavior I course at the Herbert Wertheim College of Medicine at Florida International University in Miami, FL. This session was conducted as a one-time, in-person session where all 110 medical students were in attendance at the same time. Students participating in the activity had previously learned about the mechanisms, indications, and adverse effects of these drugs in a didactic lecture series during the pharmacology course 1 year prior. Students were instructed to review a faculty-prepared study guide adapted from the didactic lectures delivered during the first-year pharmacology course (Appendix A). The study guide covered the topics to be discussed during the FC session and was sourced from multiple pharmacology and clinical anesthesia textbooks.[14-16] The study guide was intended to guide student preparation for the session by providing a comprehensive outline of topics that needed to be understood prior to arriving for the in-class active learning session as well as by encouraging self-guided deeper inquiry into self-identified knowledge gaps. To ensure preparation and

Copyright © 2024 Nunez et al. This is an open-access publication distributed under the terms of the Creative Commons Attribution license.

identify remaining knowledge gaps, students were expected to complete a 10-minute five-item multiple-choice question (MCQ) readiness quiz online prior to the start of the session (Appendix B). After submission of this readiness quiz, students received immediate feedback, along with the answers and explanations, via the online quiz platform. All FC session readiness quizzes administered within the course contributed to 4% of the total overall course grade, with this specific session quiz being worth 0.57%.

This FC session was conducted over 2 hours and occurred during the second week of the 6-week neuroscience course. It was cofacilitated by two faculty members (one pharmacologist and one board-certified anesthesiologist) in January 2022. The FC session occurred in a large-group format with learners seated in self-selected groups of three to four students working together to answer guided inquiry questions. There were 36 groups (34 groups of three students and two groups of four students), with two to three groups per table distributed across 16 triangular tables. Case elements were progressively revealed through a hard copy in-class student worksheet (Appendix C) and a projected PowerPoint presentation (Appendix D). The PowerPoint slides were displayed to the large group in presenter view such that the built-in animations were activated, and the correct answers were revealed to the large group in a stepwise fashion as the clinical case progressed. After each application exercise, one numbered student group was selected by random number generator and was asked to share its answers with the larger group. Once a group number had been selected, it was removed from the list such that the group would not be called again until all group numbers had been called at least once. Facilitators reviewed the facilitator guide (Appendix E) prior to the session and were instructed to avoid didactic teaching and instead to guide the discussion with probing questions, manage time, maintain order, and ensure that each question was discussed thoroughly. At the end of the session, students completed a 10-minute five-item MCQ consolidation quiz designed to test application of knowledge gained. The consolidation quiz was administered in the classroom and did not contribute to the overall course grade. After quiz submission, students received immediate feedback, including answers and explanations to each question.

Knowledge outcomes were assessed through analysis of the readiness assessment and consolidation quiz results. The quiz questions were developed by the faculty pharmacologist and anesthesiologist who facilitated the session at the time the FC activity was created. The questions were designed to assess either content recognition (readiness assessment) or application of a concept (consolidation quiz). The presession readiness quiz (Appendix B) and postsession consolidation quiz (Appendix F) were provided with the accompanying answers and explanations. Additionally, two case-relevant MCQs selected from the National Board of Medical Examiners (NBME) database were included on the course midterm and one on the course final exam. The NBME exam questions are not provided here due to the strict copyright restrictions imposed by the NBME limiting reproduction and publishing in any form to ensure exam security and integrity across participating institutions and administrations.

Student satisfaction was evaluated through an anonymous survey (Appendix G) with four items rated on a 5-point Likert scale (1 = *Strongly Disagree,* 5 = *Strongly Agree*) and one free-response question. The survey was administered immediately after the FC session and separately from the consolidation quiz. The satisfaction survey was administered by the College of Medicine Office of Assessment and Testing, which ensured anonymity of the responses. The survey results were subsequently provided to course faculty in a standardized, deidentified format. Postsession survey completion did not contribute to the overall course grade.

Descriptive statistics (including means and standard deviations, as well as difficulty and discrimination indices) were provided by the learning management system used to administer the exam (CanvasMed) for both the readiness assessment and the consolidation quiz and by the NBME for the midterm and final exams. The midterm was administered during week 3 of the 6-week course, and the final exam was administered on the last day of the course. Descriptive statistics for satisfaction data (means and standard deviations) were calculated by E-Value in response to the survey.

This project was reviewed by the Florida International University Institutional Review Board (IRB) and received IRB exempt approval (#IRB-110523).

Results

A total of 110 students participated in the FC session, with 110 (100%) completing the readiness assessment, 105 (95%) completing the consolidation quiz, and 110 (100%) completing the NBME midterm and final exams. Overall mean performance on the readiness assessment was 96% ($SD = 0.5$), with item difficulty levels ranging from 0.9 to 1.0 and discrimination indices ranging from 0.0 to 0.9. Overall mean performance on the postsession consolidation quiz was also 96% ($SD = 0.5$), with item difficulty levels ranging from 0.9 to 1.0 and discrimination indices ranging from 0.0 to 0.8 (Table 1).

Copyright © 2024 Nunez et al. This is an open-access publication distributed under the terms of the Creative Commons Attribution license.

Table 1. Pre- and Postsession Assessment Performance Results

Question	% Correct	*SD*	Discrimination Index	Question Type
Presession readiness assessment (*n* = 110)				
1	97	0.2	0.6	Recognition
2	85	0.4	0.9	Application
3	100	0.0	0.0	Recognition
4	100	0.0	0.0	Recognition
5	96	0.2	0.6	Recognition
Total	96	0.5		
Postsession consolidation quiz (*n* = 105)				
1	97	0.2	0.5	Recognition
2	95	0.2	0.5	Application
3	94	0.2	0.6	Recognition
4	99	0.1	0.2	Application
5	93	0.3	0.8	Application
Total	96	0.5		

Mean class performance on the three case-relevant NBME questions included on the midterm and final exam was higher than the national cohort mean for those same questions (94% vs. 72%, $p < .005$). Item difficulty levels for participants ranged from 0.9 to 1.0 and showed discrimination indices ranging from 0.2 to 0.5 (Table 2).

A total of 76 students (69%) completed the satisfaction survey. Mean overall satisfaction was 4.6 ($SD = 0.7$), with 71 students (93%) agreeing or strongly agreeing that the assigned reading material adequately prepared them for the FC session, 71 students (93%) agreeing or strongly agreeing that the FC session facilitated their learning of the basic pharmacology relevant to anesthetic drugs, 71 students (93%) agreeing or strongly agreeing that the FC session facilitated their understanding of the clinical application of anesthetic drugs, and 72 students (95%) agreeing or strongly agreeing that the time spent discussing the cases with their peers contributed to their understanding of the subject matter (Table 3).

Discussion

Despite the critical role that sedating and anesthetic agents play in modern medical practice, many medical schools either completely lack preclinical teaching covering the pharmacology of these drugs or teach it through traditional didactic lectures with limited integration of applied clinical reasoning.[5] The FC session that we developed and describe herein is a first step in addressing this unmet educational need to provide structure for the teaching of sedative and anesthetic pharmacology in the preclinical curriculum through active learning. Additionally, this session has been designed to be adaptable and can be modified to fit the curricular needs of other institutions, including for use as part of third- or fourth-year clinical rotations.

Table 2. Session-Specific Midterm and Final Exam Item Performance

Item	Average per Item: Class	Average per Item: National Board of Medical Examiners	Difference	Discrimination Index
Midterm exam				
Nitrous oxide	.88	.63	.25	0.2
Lidocaine	.98	.90	.08	0.2
Final exam				
Midazolam	.96	.68	.28	0.5

Students performed well on the presession readiness assessment, suggesting that the presession study guide provided adequate preparation for the activity. Students also performed well on the postsession consolidation quiz, suggesting adequate knowledge acquisition and ability to apply the knowledge to clinical questions. Interestingly, mean performance was the same on both assessments. While postsession scores were not higher than presession scores, it is important to note that the questions were not identical and were designed to test different things (readiness vs. consolidation). Since students were provided with immediate feedback including answers and explanations after the presession quiz, the questions included on the postsession quiz intentionally covered different topics, limiting the direct comparability of the two quizzes. Additionally, the presession quiz (Appendix B) primarily featured recognition-type questions while the postsession quiz (Appendix F) comprised a larger percentage of application-type questions. This design choice to create a postsession quiz with a higher percentage of application-type questions underscored our expectation that, after engaging in the case-based session, students would be adept at applying the foundational knowledge they had acquired to solve clinical scenarios.

As demonstrated by student performance on the NBME midterm and final exam questions, the FC activity served as an effective teaching tool leading to good short-term retention at minimum. When compared to national averages, students performed well on all three questions. The low discrimination indices seen for each of the questions indicates that both higher- and lower-performing students (based on overall performance on the exam) were able to answer the questions correctly, suggesting that the session facilitated learning of the concepts for lower-performing students as compared with their performance on topics not covered through similar sessions. Despite the limited number of questions used to assess knowledge acquisition, the high performance on these questions and the quality level of student participation in class suggest that teaching this subject matter through the FC method was successful for exam preparation.

Copyright © 2024 Nunez et al. This is an open-access publication distributed under the terms of the Creative Commons Attribution license.

Table 3. Postsession Survey Results

Question	Total Responses	*M*[a]	*SD*
The assigned reading material adequately prepared me for the session.	74	4.6	0.6
The session facilitated my learning of the basic pharmacology relevant to anesthetic drugs.	75	4.6	0.7
The session facilitated my understanding of the clinical application of anesthetic drugs.	76	4.6	0.7
The time spent discussing the cases with my peers contributed to my understanding of the subject matter.	76	4.5	0.8

[a]Rated on a 5-point Likert scale (1 = *Strongly Disagree*, 5 = *Strongly Agree*).

Student satisfaction was found to be high, an important measure of learner engagement. Free-text comments received on the satisfaction survey indicated that students especially appreciated the conciseness of the study guide and the opportunity to review previously learned pharmacology concepts. However, some students remarked that they did not have adequate time to prepare for the session due to other curricular demands, and other students commented that they prefer traditional lectures followed by time to study alone.

This FC session has been designed with adaptability in mind, recognizing that the pharmacology of sedating and anesthetic agents may be taught at different times in the curricula at varying institutions. Additionally, variations in student cohort sizes and physical space limitations may require modification of the session to fit unique institutional and curricular needs. As an example from our own experience, the idea for this activity was first conceived in 2019 with the intent to be delivered in January 2021. Unexpectedly, due to the COVID-19 pandemic and associated room occupancy limits implemented as part of the response, the session had to be modified to fit a hybrid approach. The hybrid classroom approach involved approximately half of the cohort in person and half joining remotely via the Zoom virtual meeting platform. Similarly, the students in the virtual session were divided into groups of three to four and placed in virtual breakout rooms for each application exercise. The hybrid session was not run for data collection or publication purposes, so attendance and performance metrics were not collected. Our experience implementing this session in both hybrid and in-person formats suggests that this FC activity is versatile and can be easily adapted to fit a wide variety of classrooms, cohort sizes, and preferences for virtual learning.

Although our project was able to demonstrate short-term knowledge acquisition as demonstrated by high scores on the NMBE midterm and final exam questions in comparison with national averages, we recognize several limitations. First, we are unable to comment on the efficacy of the session in terms of long-term knowledge retention. Since these topics were relatively new to the second-year curriculum after implementation of this session and had not been tested via locally created or NBME questions in previous years, we do not have equivalent local data to provide a means for comparison with previous cohort years. Only 76 of the 110 students who participated in the session completed the postsession satisfaction survey. This may be because the survey was not graded, thereby decreasing the participation rate. Although the response rate of 69% was consistent with prior satisfaction surveys administered to this cohort, the possibility of nonresponse bias cannot be excluded.[17] Because the results of the satisfaction survey were provided to course faculty in a standardized, deidentified format, we have limited ability to conduct additional calculations surrounding the satisfaction survey results without access to the raw data. Furthermore, the presession readiness quiz and the postsession consolidation quiz were not identical. Faculty at other institutions may consider adjusting the pre- and postsession quizzes to fit the needs of their course, particularly if the pre- and postsession quizzes are to be used for direct comparison.

The active learning session described herein is designed for preclinical medical students learning foundational pharmacology topics specific to sedating and anesthetic agents. This session, which uses a case-based FC format requiring students to translate foundational science knowledge to clinical application, was found to be a highly satisfactory and effective teaching tool.

Appendices

A. Study Guide.docx
B. Presession Readiness Quiz.docx
C. In-Class Student Worksheet.docx
D. Clinical Case Slides.pptx
E. Facilitator Guide.docx
F. Postsession Consolidation Quiz.docx
G. Postsession Satisfaction Survey.docx

All appendices are peer reviewed as integral parts of the Original Publication.

Copyright © 2024 Nunez et al. This is an open-access publication distributed under the terms of the Creative Commons Attribution license.

Daniella Nunez: Fourth-Year Medical Student, Florida International University Herbert Wertheim College of Medicine

Richard I. Suarez, JM: Fourth-Year Medical Student, Florida International University Herbert Wertheim College of Medicine

Melanie Molina: Third-Year Medical Student, Florida International University Herbert Wertheim College of Medicine

Gagani Athauda, MD: Professor, Alice L. Walton School of Medicine; Adjunct Professor, Department of Medical Education, Florida International University Herbert Wertheim College of Medicine; ORCID: https://orcid.org/0000-0003-2920-4077

Rebecca L. Toonkel, MD: Associate Professor, Department of Medical Education, Florida International University Herbert Wertheim College of Medicine; ORCID: https://orcid.org/0000-0001-8339-2107

Jenny Fortun, PhD: Associate Professor, Department of Medical Education, Florida International University Herbert Wertheim College of Medicine; ORCID: https://orcid.org/0000-0001-7343-9434

Nicholas V. Mendez, MD: Assistant Professor, Department of Anesthesia and Perioperative Care, University of California, San Francisco, School of Medicine; Adjunct Assistant Professor, Department of Medical Education, Florida International University Herbert Wertheim College of Medicine; ORCID: https://orcid.org/0000-0002-0507-8726

Disclosures

None to report.

Funding/Support

None to report.

Ethical Approval

The Florida International University Institutional Review Board deemed further review of this project not necessary.

References

1. Dobson GP. Trauma of major surgery: a global problem that is not going away. *Int J Surg*. 2020;81:47-54. https://doi.org/10.1016/j.ijsu.2020.07.017
2. Wiernik PH; Public Policy Committee of the American College of Clinical Pharmacology. A dangerous lack of pharmacology education in medical and nursing schools: a policy statement from the American College of Clinical Pharmacology. *J Clin Pharmacol*. 2015;55(9):953-954. https://doi.org/10.1002/jcph.539
3. Donnenberg VS, Burris JF, Wiernik PH, Cohen LJ, Korth-Bradley JM. How to fix the dangerous lack of clinical pharmacology education in the medical profession: the generation of core entrustable professional activities in clinical pharmacology for entering residency. *J Clin Pharmacol*. 2016;56(10):1177-1179. https://doi.org/10.1002/jcph.748
4. *USMLE Content Outline*. Federation of State Medical Boards of the United States/National Board of Medical Examiners; 2021. Accessed October 16, 2024. http://www.usmle.org/pdfs/usmlecontentoutline.pdf
5. Curry SE. Teaching medical students clinical anesthesia. *Anesth Analg*. 2018;126(5):1687-1694. https://doi.org/10.1213/ANE.0000000000002802
6. Goldhaber-Fiebert S, Lei V, Bereknyei Merrell S, Nandagopal K. Perioperative emergency manuals in clinical clerkships: curricula on "why, how, and when to use" for teaching medical students. *MedEdPORTAL*. 2015;11:10056. https://doi.org/10.15766/mep_2374-8265.10056
7. Boggs SD, Luedi MM. Nonoperating room anesthesia education: preparing our residents for the future. *Curr Opin Anaesthesiol*. 2019;32(4):490-497. https://doi.org/10.1097/ACO.0000000000000743
8. Kurup V, Hersey D. The changing landscape of anesthesia education: is flipped classroom the answer? *Curr Opin Anaesthesiol*. 2013;26(6):726-731. https://doi.org/10.1097/ACO.0000000000000004
9. Hurtubise L, Hall E, Sheridan L, Han H. The flipped classroom in medical education: engaging students to build competency. *J Med Educ Curric Dev*. 2015;2:23895. https://doi.org/10.4137/JMECD.S23895
10. O'Brien BC, Irby DM. Enacting the Carnegie Foundation call for reform of medical school and residency. *Teach Learn Med*. 2013;25(suppl 1):S1-S8. https://doi.org/10.1080/10401334.2013.842915
11. Ramnanan CJ, Pound LD. Advances in medical education and practice: student perceptions of the flipped classroom. *Adv Med Educ Pract*. 2017;8:63-73. https://doi.org/10.2147/AMEP.S109037
12. Graffam B. Active learning in medical education: strategies for beginning implementation. *Med Teach*. 2007;29(1):38-42. https://doi.org/10.1080/01421590601176398
13. Akçayir G, Akçayir M. The flipped classroom: a review of its advantages and challenges. *Comput Educ*. 2018;126:334-345. https://doi.org/10.1016/j.compedu.2018.07.021
14. Miller RD, Pardo MC Jr. *Basics of Anesthesia*. 6th ed. Elsevier/Saunders; 2011.
15. Butterworth JF, Mackey DC, Wasnick JD. *Morgan and Mikhail's Clinical Anesthesiology*. 7th ed. McGraw Hill; 2022.
16. Clark MA, Finkel R, Rey JA, Whalen K, eds. *Lippincott's Illustrated Reviews: Pharmacology*. 5th ed. Lippincott Williams & Wilkins; 2012.
17. Story DA, Tait AR. Survey research. *Anesthesiology*. 2019;130(2):192-202. https://doi.org/10.1097/ALN.0000000000002436

Received: November 27, 2023
Accepted: July 24, 2024
Published: November 8, 2024

Copyright © 2024 Nunez et al. This is an open-access publication distributed under the terms of the Creative Commons Attribution license.
